# Validation of Aptamer–Protein Interactions by Biolayer Interferometry: A Study of 88 Candidate Molecules

**DOI:** 10.3390/ijms27198469

**Published:** 2026-09-23

**Authors:** Elizaveta S. Ershova, Natalia V. Pozdnyakova, Aleksandra Yu. Egorshina, Elena V. Yakushenko, Igor V. Dobrokhotov, Nadezhda P. Lyamina, Bella B. Gagulaeva, Alexey Moskalev

**Affiliations:** 1 Petrovsky National Research Center of Surgery, Institute of Biology of Ageing and Healthy Longevity Medicine with Clinic of Predictive Medicine, 119435 Moscow, Russiae-yakushenko@mail.ru (E.V.Y.); hum.d@yandex.ru (I.V.D.); gagulati@yandex.ru (B.B.G.); 2 SistemaBioTech, LLC, 1-ya Kuryanovskaya Str. 34, 109235 Moscow, Russia; n.pozdnyakova@sistemabiotech.ru (N.V.P.); a.egorshina@sistemabiotech.ru (A.Y.E.)

**Keywords:** DNA aptamers, biolayer interferometry, BLI, Octet, binding validation, nonspecific binding, negative result, data quality control, computational oligonucleotide design

## Abstract

An aptamer’s low mass confines the label-free response to hundredths of a nanometer, where artifacts mimic binding yet yield computable dissociation constants. To define and test a priori criteria distinguishing binding from artifact, 88 computationally designed aptamers against seven human proteins were studied by biolayer interferometry in two orthogonal configurations, with control antibodies. Six criteria, fixed in advance, are reported as a priori decision rules, not validated requirements. Control antibodies validated 6 of 8 preparations; for the other 68 pairs, the maximum response was 0.019–0.219 nm, bounding *K*_D_ at ≥8.6 µM, 120-fold above the 70 nM threshold. Two of three signals clearing it gave exponents far above the 1:1 expectation (1.04 vs. 0.31; 0.85 vs. 0.41); the third failed parameter consistency (*K*_D_ scatter ×28). Rankings on two PCSK9 preparations did not correlate (ρ = −0.26); predicted and experimental *K*_D_ differed by 3–7 orders of magnitude (ρ = 0.497, *n* = 25). No aptamer reached the predefined affinity under the conditions tested. Local fitting returns internally consistent *K*_D_ for data incompatible with a 1:1 model; we propose isotherm verification and antibody validation of every preparation as minimal requirements. No positive-control aptamer was available, limiting interpretation.

## 1. Introduction

Aptamers are oligonucleotides that acquire a tertiary structure upon folding and bind their target noncovalently. Their appeal for diagnostics stems from chemical synthesis instead of cell culture, storage stability, batch-to-batch reproducibility, and low cost [1,2,3]. Practical implementation hinges on obtaining a molecule with an affinity comparable to that of antibodies: formats such as ELASA (an enzyme-linked assay in which the aptamer serves as the detector) require a *K*_D_ on the order of tens of nanomoles per liter or lower, together with kinetics that tolerate washing steps [4,5].

The classical route to such molecules, SELEX, is labor-intensive, which is why computational approaches are being actively developed: modeling of the oligonucleotide tertiary structure, docking to the target, and ranking of candidates by predicted affinity. Such approaches promise to reduce the experimental stage to the testing of a dozen molecules, partially replacing the labor-intensive rounds of SELEX [6,7]. How well a prediction transfers to experiment can be shown only by the experimental test itself, so the outcome depends on the quality of that test.

Biolayer interferometry has become the standard for primary validation [8,9]. The method records a shift in the interference pattern proportional to the thickness of the layer at the biosensor tip, that is, to a first approximation, to the mass of bound material. This imposes a limitation on work with aptamers: the molecular mass of an aptamer is 8–10 times lower than that of an IgG, so at equal surface occupancy, an aptamer yields a response 8–10 times smaller. If an antibody under the same conditions gives 1–2 nm, complete saturation of the surface by an aptamer gives only 0.1–0.3 nm, whereas instrumental noise and baseline drift amount to about 0.01 nm and nonspecific sorption on the biosensors reaches up to a few tenths of a nanometer. The working signal range for an aptamer lies one order of magnitude above the noise level, whereas for an antibody, it lies two to three orders above [8].

A consequence of this is rarely discussed explicitly: the instrument software will compute *k*_on_, *k*_off_, and *K*_D_ for any curve, including a curve generated entirely by an artifact. The resulting number looks like a measurement and finds its way into publications and specifications. For this reason, reports of computationally designed aptamers with nanomolar affinity require independent verification.

The present work arose from a practical task: to select aptamers against seven human proteins for an ELASA-format assay. We obtained a dataset that is unusually homogeneous: 88 oligonucleotides of a single chemical type, synthesized in one laboratory, seven targets, nine protein preparations, two orthogonal experimental configurations, control antibodies in every run, and a complete set of reference biosensors. Such a dataset makes it possible to determine which procedural requirements distinguish an interpretable BLI experiment with aptamers from a non-interpretable one, rather than merely reporting the result for specific molecules.

We frame the task as follows: (1) to fix the signal-acceptance criteria before data analysis; (2) to apply them to the entire dataset; (3) to show, with examples, which data pass the criteria and which are rejected and why; and (4) to assess which quantitative statements a negative result permits.

## 2. Results

### 2.1. Synthesis Quality and Panel Structure

All 88 compounds passed quality control (Table 1). Purity was 85–92% (median 89%), yield 81–93% (median 88%), and product mass 0.32–0.78 mg. We found no difference in purity between the non-biotinylated and biotinylated batches; however, the biotinylated batches showed a somewhat wider spread of yield (81–93% versus 85–92%).

A practically important conclusion follows: the quality of the oligonucleotide material did not limit the experiment. Differences in the behavior of the molecules on the biosensor cannot be explained by differences in purity, because the purity spread did not exceed 7 percentage points, whereas the responses differed qualitatively, ranging from zero to negative values.

The origin of the panel bears on how these observations should be read. The 88 sequences are the complete set of top-ranked candidates delivered by the developer of the computational method for the seven targets: 10 per target in the non-biotinylated format, except for MMP2 (9), together with 19 5′-biotinylated candidates against MMP2 (9) and PCSK9 (10) prepared for configuration II. The authors carried out no further down-selection beyond screening for synthesis feasibility. The predicted (in silico) *K*_D_ values supplied with the sequences span 54.5–218 pM for IL6, the nanomolar-to-submicromolar range for MMP2, MMP9, MMP10, PCSK9 and sRAGE, and 752–2819 µM for ESM1 (Section 4.1); the full list of sequences with their predicted *K*_D_ values and the corresponding experimental responses is provided in Supplementary Information S1 (Tables S1 and S2). The design pipeline that generated them (structure prediction, docking, scoring, and training data) is proprietary to the developer and was not disclosed to the authors. The panel can therefore be judged by its output, but the experimental outcome reported below constrains that output as a whole and cannot be attributed to any particular stage of the prediction (Study Limitations, item 9).

### 2.2. Validation of the Analytical System with Control Antibodies (Criterion C1)

The control antibodies produced high-affinity binding for six of the eight protein preparations (Table 2). The range of responses of 0.28–2.49 nm at antibody concentrations of 5–200 nM and the computed *K*_D_ from 3.2 × 10^−11^ to 3.2 × 10^−8^ M (for some curves below the method’s limit of accurate determination, <10^−12^ M) show that the analytical system as a whole (instrument, biosensor chemistry, buffer, experimental scheme, and processing) is capable of detecting and quantitatively characterizing high-affinity interactions. The scale of the difference between aptamers and antibodies is directly visible in the sensorgrams for IL6 (Figure 1).

The practical sensitivity of the system is set by the MMP9 series: the control antibody at 5 nM gave a response of 0.069–0.071 nm. The aptamers in the same series at 10 µM, a 2000-fold higher concentration, gave a maximum response of 0.094 nm.

In accordance with criterion C1, we excluded from the binding analysis all data for the MMP10 target (10 aptamers), the PCSK9 (Elabscience) preparation, and the ESM1 target (10 aptamers). Of the 88 compounds, 68 thus remained in interpretable experiments: 49 non-biotinylated (IL6, MMP2, MMP9, PCSK9, sRAGE) and 19 biotinylated.

We regard the fraction of non-validatable systems—two preparations out of eight—as a result in its own right. In planning such work, one should assume that about one quarter of the purchased protein preparations, or protein–detector antibody pairs, will prove unsuitable, and should incorporate at least two independent control reagents per target into the design.

### 2.3. Noise Level and Detection Threshold

Sensorgrams for analyte-free buffer solutions showed baseline fluctuations of 0.01 nm and above when the biosensor was transferred between wells. In the MMP9 and MMP10 series, we observed signal drift on all biosensors, including the control, which required subtraction of the reference-biosensor signal for the entire series. In configuration II, subtraction gave negative response values down to −0.33 nm; nonspecific sorption of the protein on the aptamer-free biosensor exceeded sorption on the aptamer-bearing biosensor.

A negative response after correction carries information about the system: the nonspecific component of the signal is comparable in magnitude to the expected specific component, and in uncorrected data, it would be taken for binding. In our dataset, 18 of 27 measurements in the MMP2/SAX2 series gave a negative response after correction.

### 2.4. Aptamer Responses and Estimation of the Lower Bound of K_D_

In the validated systems, the maximum response shown by any aptamer ranged from 0.019 nm (MMP2, configuration I) to 0.219 nm (sRAGE, configuration I), at concentrations exceeding the predicted *K*_D_ by 10^2^–10^5^-fold (Table 3). For comparison, the control antibodies in the same series, at concentrations 100–2000-fold lower, gave responses one to two orders of magnitude larger. A summary across all systems is shown in Figure 2.

The values given define an upper bound of affinity rather than measure it. The correct formulation of the result is: “an interaction with *K*_D_ ≤ 10^−5^ M was not detected under these conditions.” The formulation “the aptamers do not bind” is incorrect; it contains no indication of the method’s sensitivity and does not admit falsification. Throughout this paper, the result is accordingly stated as an absence of detectable affinity under the conditions tested, which is what these data establish, rather than as a property of the molecules in general.

### 2.5. Orthogonal Configuration and Candidates That Passed the Threshold Criterion

Configuration II removes two objections to configuration I: shielding of the binding site upon immobilization of the protein through its histidine tag, and insufficient analyte mass. In configuration II, the analyte is a protein of 70–90 kDa, which moves the experiment into a regime favorable for sensitivity. The immobilization step here also serves as a sensitivity check: all 19 biotinylated aptamers from a 25 nM solution gave a response of about 0.5 nm; that is, the instrument reliably records the accumulation of aptamer-sized molecules on the biosensor surface.

In the MMP2/SAX2 series, the maximum response was 0.072 nm (aptamer 161_13 at 253.8 nM), with the curve rising during the dissociation phase, which precludes its processing, while at lower protein concentrations, the signal for this same aptamer was absent. Thus, none of the nine biotinylated aptamers against MMP2 passed criteria C2 and C5 (Figure 3).

In the PCSK9/SAX2 series, three aptamers (161_1, 161_2, and 161_5) gave responses above the threshold (up to 0.56 nm) with a monotonic dependence on protein concentration and a formal *K*_D_ in the range 1.5 × 10^−7^–6.7 × 10^−6^ M. Within this series, these are the only pairs that passed criteria C2 and C3, and we examine them in detail alongside the best-responding pair of each of the other validated systems (Figure 4 and Table 4).

**Table 4:** Testing of candidates that passed the threshold criterion for compatibility with a 1:1 model. Formal *K*_D_ values are local-fit estimates obtained separately at each concentration. The standard errors returned by the analysis software for *K*_D_, ***k*****_on_** and ***k*****_off_** are tabulated for every curve in Supplementary Information S3; the corresponding approximate 95% confidence intervals are ±1.96 × SE. No confidence interval is given for the exponent n, which is derived from only two or three concentrations without replicate measurements at each and is therefore reported as a point estimate.

Aptamer	Target, Config.	*C* Range	Formal *K*_D_, M	*K*_D_ Scatter Across *C*	*k*_on_ Scatter	*n* Observed	*n* Expected for 1:1
161_2	PCSK9, II	111–1000 nM	1.5–3.2 × 10^−7^	×2.2	×3.0	0.85	0.41
161_1	PCSK9, II	333–1000 nM	2.4–2.7 × 10^−7^	×1.2	×1.0	1.04	0.31
161_5	PCSK9, II	333–1000 nM	2.4 × 10^−7^–6.7 × 10^−6^	×27.6	×10.5	0.60	0.85
140_54	sRAGE, I	1–10 µM	3.9 × 10^−7^–1.2 × 10^−4^	×319	×1.8	0.87	0.13
140_1	IL6, I	1–10 µM	2.3 × 10^−8^–4.4 × 10^−7^	×19	×18	−0.12	0.14
132_1	PCSK9 (in-house), I	1–10 µM	6.0 × 10^−6^	—	—	0.27	0.64

Aptamer 161_1 gave the most convincing picture: the constants computed independently at the two concentrations agree within 20%, and *k*_on_ agrees almost exactly. Such a result is usually taken as evidence that the model is correct. However, the test of the shape of the concentration dependence (criterion C5) refutes it (Figure 5): the response grows in proportion to concentration (*n* = 1.04) over the entire studied range, whereas for an interaction with *K*_D_ ≈ 254 nM at concentrations of 333–1000 nM, that is, already above *K*_D_, pronounced saturation is expected (*n* = 0.31). The observed linearity means the absence of a limited number of binding sites, which is incompatible with an affinity interaction of 1:1 stoichiometry and is characteristic of nonspecific accumulation of material on the surface.

**Figure 5:**
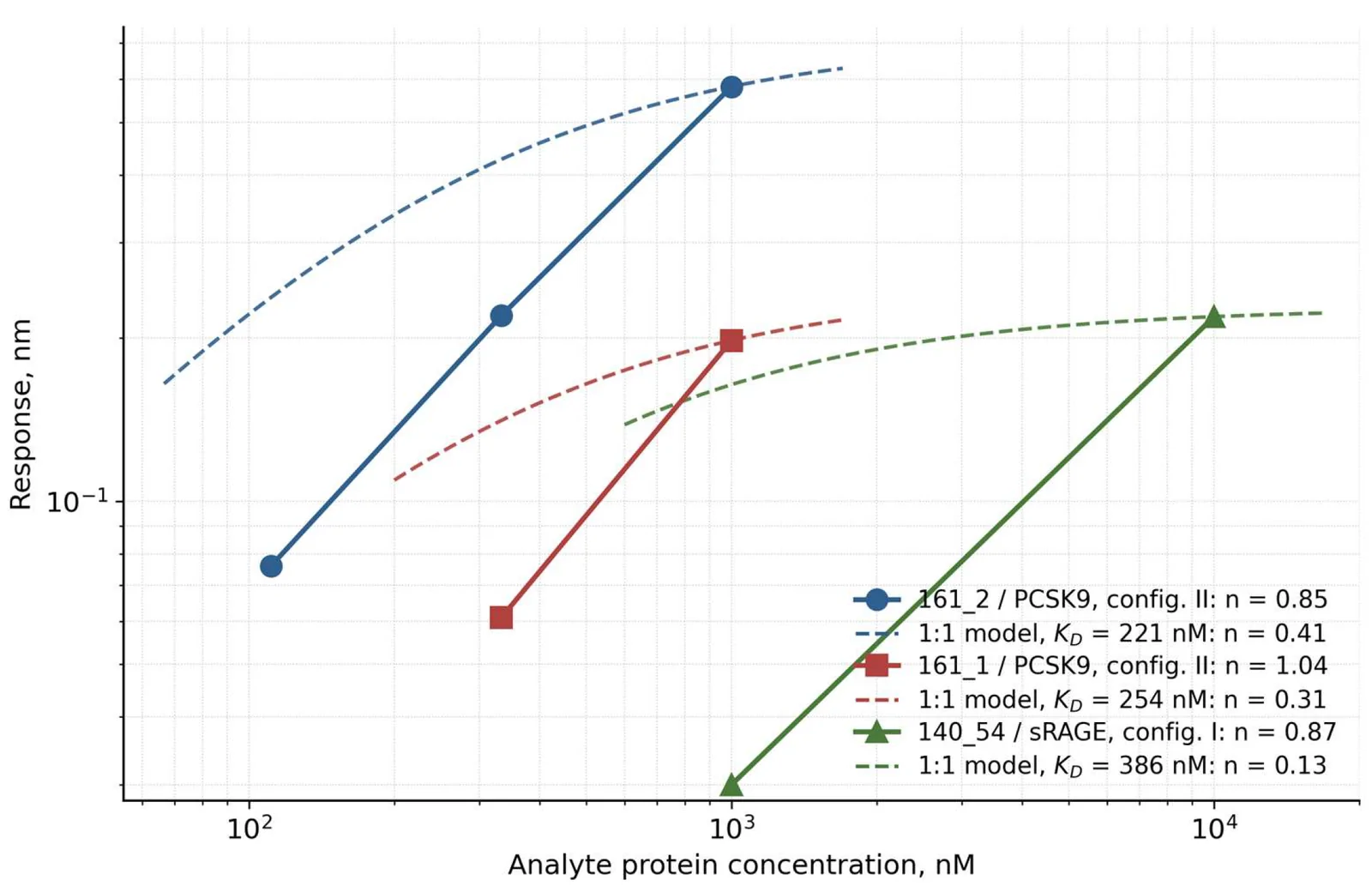
Response versus analyte concentration in double-logarithmic coordinates. Solid lines represent the observed values; dashed lines the 1:1-model isotherm for the constant obtained by kinetic fitting, normalized to the top point of each series. The exponent n is indicated; the observed values are close to unity, corresponding to the absence of saturation.

Aptamer 161_2 behaves similarly (*n* = 0.85 versus the expected 0.41), as does 140_54 against sRAGE (*n* = 0.87 versus 0.13; the response increased 7.4-fold for a tenfold increase in concentration, whereas a model with *K*_D_ = 386 nM allows an increase of only about one-third, since at 1 µM the occupancy would already be 72%). Aptamer 161_5 is a different case. Its observed exponent (0.60) lies below, not above, the value expected for the constant obtained by fitting (0.85), so the isotherm test alone would not reject it. The constants themselves, however, are not stable enough for that comparison to carry weight: across the two concentrations, *K*_D_ varies by a factor of 27.6 and k_on by 10.5 (Table 4). This pair is therefore rejected by criterion C4, on the inadequacy of the model to the data, before the shape of the concentration dependence is reached.

Aptamer 140_1 against IL6 showed an inverse dependence: the response at 1 µM (0.056 nm) exceeds that at 10 µM (0.043 nm), i.e., the concentration dependence is negative (*n* = −0.12). At the same time, the software returned a *K*_D_ of 23 nM for it from the curve at 10 µM, a value that formally satisfies the assay requirement. Numbers extracted from noise curves find their way into publications when criteria C3–C5 are not applied.

The data from the two configurations also disagree with each other. Aptamer 161_2 and the non-biotinylated aptamer 132_2 are the same sequence. In configuration II, it gave a formal *K*_D_ of 2.0 × 10^−7^ M, and in configuration I against the same protein preparation, 3.2 × 10^−6^ M, which is 16-fold worse. For 161_1/132_1, the discrepancy was 25-fold. For a specific interaction, the dissociation constants determined in two orthogonal configurations may be expected to differ by 2–5-fold owing to steric effects and avidity, but by no more than an order of magnitude. A discrepancy of 16–25-fold exceeds this range and indicates that at least one of the two signals is nonspecific in nature or arises from an immobilization artifact.

### 2.6. Dependence of the Signal on the Target Preparation (Criterion C6)

Ten non-biotinylated aptamers against PCSK9 were studied under identical conditions against two independent preparations of the target: the in-house preparation (histidine tag only) and a commercial preparation with an additional biotinylated AVI tag. The ranking of candidates by response magnitude was not reproduced across the two preparations (Table 5 and Figure 6): the Spearman rank correlation coefficient was −0.26 (*n* = 10); using the exact permutation distribution for Spearman’s ρ at this sample size rather than the t approximation, the two-tailed *p*-value is ≈0.47, and the 95% confidence interval for ρ (Fisher z-transformation) is approximately [−0.77, 0.44].

The extreme positions are inverted: the leader on one preparation ended up last on the other, and vice versa. In addition, the AVI-tagged preparation systematically gave higher responses: seven of the ten aptamers exceeded 0.07 nm, versus one on the preparation without the AVI tag.

The most parsimonious explanation, which we cannot confirm with the available data, is that the signal is driven not by the target but by the additional modification of the preparation. Binding of oligonucleotides to the tags with which a protein is expressed has been described in the literature [10]. This remains a hypothesis rather than a demonstrated fact; testing it would require a control experiment with a protein carrying only an AVI tag, or with the tag itself. An orthogonal measurement of the same ten aptamers against both preparations, by surface plasmon resonance, microscale thermophoresis, or isothermal titration calorimetry, would settle the question, and we name it as a priority for follow-up work (Study Limitations, item 2). For practical purposes, the fact itself is sufficient: the ranking of candidates changed completely upon replacement of the target preparation, so selection based on such a signal is meaningless.

### 2.7. Summary of the Application of the Criteria

Of the 88 synthesized molecules, 20 were eliminated at the C1 stage together with the non-validatable target preparations. Of the remaining 68, criterion C2 (a reference-subtracted response of 0.1 nm or above, or within the 0.05–0.1 nm band that C2 admits with an explicit caveat) was passed by 6 aptamer–protein pairs. Of these, criterion C3 (adequacy of errors) was passed by 5, criterion C4 (consistency of parameters across concentrations) by 3, and criterion C5 (shape of the concentration dependence) by none. Criterion C6 is applicable to the ten aptamers against PCSK9 and was not passed.

The outcome: under the conditions tested, none of the 88 molecules exhibited binding that we could classify as a specific affinity interaction.

## 3. Discussion

### 3.1. What Is and Is Not Demonstrated

The data support three statements. Under conditions reproducing those of the computational modeling, for 68 aptamer–protein pairs in validated analytical systems, we did not detect an interaction with *K*_D_ at the level of 10^−5^ M or lower. None of the six pairs that passed the response-magnitude threshold is compatible with a 1:1 binding model when the shape of the concentration dependence is tested. The quality of the synthetic material and the performance of the analytical system are excluded as explanations for the negative result: the former is confirmed by quality control of all 88 batches, and the latter by antibody controls in every series.

We do not consider it proven that the molecules studied do not bind their targets under any conditions. Our experiment was performed in a single buffer system, at a single temperature, with a single folding regime, and with proteins from a limited set of sources. Aptamers are sensitive to ionic composition, and in particular, the Mg^2+^ concentration of 1 mM, taken from the modeling specification, is below that typical of aptamer protocols (5 mM) [3,11,12,13]. In addition, the absence of detergent in the running buffer, dictated by the same consideration of correspondence with the model, increased the contribution of nonspecific binding [8,14].

The positive control in our series was an antibody rather than an aptamer with independently confirmed affinity. This bounds what the sensitivity controls can establish. The instrument records aptamer-sized molecules: immobilization of biotinylated aptamers (about 16 kDa) from a 25 nM solution gave a response of about 0.5 nm on all SAX2 biosensors. This confirms that the absence of a signal in the interaction with proteins is not due to insufficient sensitivity of the method to aptamer-sized molecules. In configuration II, the analyte was a protein of 70–90 kDa, so for the 19 pairs studied in this configuration, considerations of sensitivity to a low-mass analyte do not apply. The limitation concerns another link in the chain: no aptamer–protein pair with a *K*_D_ known from the literature was passed through the full chain of folding regime, buffer ionic composition, BLI scheme, and data processing. We therefore cannot experimentally exclude the possibility that the chosen folding regime or ionic composition suppresses binding for the entire class of molecules at once. Without a DNA aptamer of independently confirmed affinity carried through the same folding regime, buffer, immobilization chemistry, and processing pipeline used here, we cannot experimentally distinguish assay-level insensitivity to this class of interaction from a genuine absence of binding by the computationally designed candidates. Including a published aptamer with confirmed affinity (for example, from [15,16,17]) closes this gap, and we consider such a control mandatory in studies of this design. The ideal positive control for validating BLI experiments with aptamers is an aptamer with independently confirmed affinity rather than an antibody, because antibodies and aptamers differ in their binding mechanisms and in their sensitivity to conditions. The absence of such a control does not alter the primary observation (no molecule passed the criteria), but it makes the interpretation of that observation conditional: the negative result holds for these molecules under this assay chain and these conditions, and is not a general verdict on the suitability of biolayer interferometry for aptamers or on computational aptamer design as such. There is also a practical reason why such a control is not a trivial addition. An external aptamer of confirmed affinity carries its own optimized buffer, ionic composition and folding regime; run under the conditions of the present panel, which the developer of the computational method specified as those under which the 88 candidates were modeled, a negative outcome for that control would be ambiguous between an unsuitable condition set for that particular molecule and genuine insensitivity of the assay. A positive control is informative here only if its own conditions are titrated in parallel, which places it in a dedicated follow-up study rather than in an addendum to the present one.

As a complementary, quantitative view of the relationship between the computational predictions and the experimental data, we computed the Spearman rank correlation between the predicted (in silico) and experimental *K*_D_ for the 25 aptamers across the panel for which an experimental *K*_D_ could be formally computed at all (local fit 1:1 full, response ≥ 0.05 nm; the representative point per aptamer was taken at the concentration giving the strongest response). This gives ρ = 0.497 (*p* = 0.0115, *n* = 25): a weak but statistically significant positive association, indicating that the predictions broadly capture the correct rank order of affinity but explain only a modest fraction of it, while absolute values diverge by 3–7 orders of magnitude across nearly the entire panel. This association should be read with the same caution as the individual constants it is built from: the 25 experimental values are local-fit outputs, and all but the three PCSK9 candidates of Section 2.5 come from curves that did not pass criteria C2–C5, so what correlates with the prediction is a formally computable number rather than a demonstrated affinity. Eight of the 25 belong to the ESM1 panel, which was excluded from the binding analysis and anchors the weak-affinity end of the ranking; removing it leaves ρ = 0.524 (*p* = 0.031, *n* = 17), so the association does not rest on those points. Within the largest single-target subset, the twelve PCSK9 aptamers, there is no significant association (ρ = 0.26, *p* = 0.41). The correlation therefore reflects differences between targets rather than an ability to rank candidates within a target, which is the discrimination a screening application actually requires. Sequences, predicted *K*_D_ values, and this correlation dataset are provided in Supplementary Information S1 (Tables S1 and S2).

### 3.2. Why Local Fitting Is Misleading

The principal methodological result of the work is that internal consistency of the kinetic parameters is insufficient to declare an interaction specific. Aptamer 161_1 gave *K*_D_ values agreeing within 20% across the two concentrations, and *k*_on_ values agreeing almost exactly, a picture that most publications would consider convincing. Nevertheless, the equilibrium behavior of the system contradicts this model: the response is proportional to concentration over the entire range, including the region above the putative *K*_D_, i.e., there is no saturation.

The reason for the discrepancy lies in the design of the procedure itself [18,19,20]. Local fitting adjusts the shape of an individual curve, and the shape of an association curve in the non-equilibrium regime is determined mainly by the product *k*_on_·*C* and the duration of the step. A process that is linear in concentration and lacks saturation (for example, nonspecific sorption or slow accumulation on the surface) produces curves that the local 1:1 model fits successfully, returning plausible constants. The two cases can be distinguished only by comparing amplitudes at different concentrations, that is, by testing the isotherm rather than the kinetics.

Criterion C5 should always be applied when a global fit over the full concentration series is unavailable. It requires a minimum of two concentrations differing by at least threefold and demands no material beyond the measurements already being made. This is the regime of the present study. The purpose here was to identify candidates meeting the *K*_D_ ≤ 70 nM threshold, not to characterize the kinetics of each of the 88 molecules; for that purpose, two to three concentrations spanning at least a threefold range are adequate, and criterion C5 was formulated to compensate for the absence of a global fit in exactly this regime. A full multi-point global fit is the appropriate next step once a candidate has passed the screen. Running multi-point kinetics on all 88 candidates before any had confirmed specific binding would have consumed several times the material and instrument time without improving the screening decision.

### 3.3. Requirements for the Specification of Candidate Molecules

Computational methods for aptamer design usually output a single parameter, the predicted *K*_D_. Our experience shows that this is insufficient even when the prediction is correct. For an ELASA-format assay, both kinetic constants matter separately: too small a *k*_on_ lengthens the incubation step so much that the system ceases to compete with ELISA, while too large a *k*_off_ leads to loss of the complex during washing [21,22]. The control antibodies in our dataset provide an empirical benchmark for target values: *k*_on_ of 10^4^–10^6^ M^−1^·s^−1^ and *k*_off_ of 10^−5^–10^−3^ s^−1^, consistent with published requirements for immunoassays [22]. Aptamers with different kinetics are usable, but only for analytes whose concentration substantially exceeds that of the aptamer itself in the system [23], which excludes the main class of interesting targets: cytokines, growth factors, and other low-abundance proteins.

The second specification requirement concerns the range of applicability. In our panel, the predicted *K*_D_ for aptamers against ESM1 was 752–2819 µM. Such values exceed the upper limit of the range measurable on the instrument (1 mM) and are devoid of practical meaning: detection would require aptamer concentrations of a few millimoles per liter. Formally, the molecules were delivered and tested; in fact, they could not be tested in principle. The specification of a candidate should include a check on the physical feasibility of the experiment: the concentration required for validation (usually 10 × *K*_D_) must be achievable given solubility, the available amount of material, and the level of nonspecific binding.

### 3.4. Practical Recommendations

The minimal protocol for testing candidate aptamers by BLI is summarized in Table 6.

Items 1, 3, 4, 6, 7, 9, and 10 reflect established biolayer-interferometry practice recommended by the instrument manufacturer and by published aptamer BLI protocols [24,25] and were fixed before data analysis. Items 2 and 11 are likewise established practice rather than innovations of this study; they were simply not performed here. Only items 5 and 8 are put forward by this study: item 5 is a threshold calibrated on the noise floor of this instrument and analyte class (Section 4.8), and item 8 is a test standard in kinetic analysis that is, in our reading of the aptamer BLI literature, rarely applied to the short concentration series typical of screening.

Table 6 is offered as a set of a priori decision rules whose diagnostic value this dataset illustrates, not as externally validated acceptance criteria. Items 5 and 8 were the decisive ones here, but their numerical form is tied to the noise floor of this instrument and this class of analyte; converting them into general requirements calls for quantitative, instrument-independent calibration, for example, by spiking experiments with analytes of known *K*_D_ across several biolayer-interferometry platforms. We regard this as a priority for dedicated methodological work. Three experiments are, on the evidence of this study, the highest-value additions to a design of this kind, and all three lie outside the scope of the present one: a positive-control aptamer of independently confirmed affinity carried through the same chain with its own conditions titrated in parallel (item 2); a scrambled-sequence oligonucleotide of matched length and GC content on the biosensor (item 11); and a small buffer matrix varying Mg^2+^, K^+^ and detergent for representative candidates. The last of these needs a qualification specific to this study. The single buffer used here was chosen deliberately to reproduce the conditions under which the candidates were modeled, so that the experiment would test the prediction rather than the molecules in general; a response obtained in a modified buffer could not be compared with the prediction it was meant to test. The cost of that choice is that our negative result does not generalize to other ionic conditions, and a buffer matrix is the natural way to separate the contribution of the aptamers from that of the assay environment once the prediction has been tested on its own terms.

## 4. Materials and Methods

### 4.1. Panel of Candidate Molecules

The panel comprised 88 oligonucleotides: 69 non-biotinylated (10 against IL6, 9 against MMP2, 10 against MMP9, 10 against MMP10, 10 against ESM1, 10 against PCSK9, and 10 against sRAGE) and 19 5′-biotinylated (9 against MMP2 and 10 against PCSK9). The sequences and predicted *K*_D_ values were provided by the developer of the computational method; the molecules are chimeric DNA/RNA oligonucleotides carrying 2′-F-modified residues, approximately 40–60 nucleotides in length. The predicted *K*_D_ values lay in the picomolar range for aptamers against IL6 (54.5–218 pM), in the nanomolar-to-submicromolar range for aptamers against MMP2, MMP9, MMP10, PCSK9, and sRAGE, and in the range of 752–2819 µM for aptamers against ESM1. For all sequences, we performed in silico analysis of the secondary structure using mfold software (version 3.6) under ionic conditions matching the experiment (100 mM Na^+^, 1 mM Mg^2+^, 20 mM K^+^, 50 mM Tris-HCl, pH 7.4). The predicted structures of most candidates contained hairpin elements; no G-quadruplex motifs were predicted for any of the sequences. A possible influence of quadruplex folding on binding under our conditions is therefore unlikely, although the correctness of folding was not verified instrumentally. The design algorithm itself (structure prediction, docking/scoring, and any training data) is proprietary to the developer and was not disclosed to the authors beyond the final candidate sequences and their predicted *K*_D_ values (see Study Limitations, item 9). The sequences themselves, with their predicted *K*_D_ values and the corresponding experimental responses, are provided in Supplementary Information S1 (Tables S1 and S2).

### 4.2. Solid-Phase Synthesis and Quality Control

Synthesis was carried out on an automated Polygen synthesizer (Polygen GmbH, Langen, Germany) using the standard phosphoramidite cycle with 0.1 M solutions of the dA, dT, dC, dG, fA, fU, fC, and fG amidites in acetonitrile, 0.25 M dicyanoimidazole as an activator, 0.05 M iodine in pyridine/water (9:1) as an oxidizer, and xanthane hydride for the thiophosphate linkages [26]. Coupling completeness was monitored by the signal of the built-in trityl monitor. Cleavage from the solid support was performed in aqueous ammonia at 65 °C for 2 h, followed by purification on reversed-phase GlenPak cartridges (Glen Research Corporation, Sterling, VA, USA) according to the manufacturer’s protocol. The amount of product was calculated from the Beer–Lambert–Bouguer law based on UV absorbance (NanoDrop, Thermo Fisher Scientific, Waltham, MA, USA).

### 4.3. Target Proteins and Control Antibodies

We used nine protein preparations: IL6 (Cloud-Clone, Wuhan, China; APA079Hu61), MMP2 (AntibodySystem, Paris, France; EHC32301, C-His), MMP9 (Cloud-Clone, Wuhan, China; APA553Hu61), MMP10 (Sino Biological, Beijing, China; 10249-H07H), RAGE extracellular domain (Sino Biological, 11629-H08H), and PCSK9 from three independent sources, namely a commercial preparation (Elabscience, Houston, TX, USA; PDMH100413), a recombinant PCSK9 protein with a C-terminal 6xHis-Tag (Sistema-BioTech, Moscow, Russia; sbt2047175), and a commercial preparation with an additional biotinylated AVI tag (Sino Biological, 29698-H27H-B), as well as ESM1.

Each run included a control antibody against the same target: anti-IL6 (clone SAA0375, FHC15810), anti-MMP2 (RHC32301), anti-MMP9 (clone R3D69, RHD06804), anti-MMP10 (PHC39701), anti-RAGE (VHH01903), anti-PCSK9 (RMJ24001), and the therapeutic antibody evolocumab (Repatha). No control antibody was used for the ESM1 target.

### 4.4. Folding and Buffer Conditions

The developer of the computational method specified the conditions under which the modeling had been performed: pH 7.4, Na^+^ 100 mM, Mg^2+^ 1 mM, K^+^ 20 mM. We reproduced them in a buffer of 50 mM Tris-HCl, pH 7.4, 100 mM NaCl, 1 mM MgCl_2_, and 20 mM KCl, and used this buffer both for folding and as the running buffer in BLI. No detergent was added, so as not to introduce a component absent from the model. Folding was performed at 10 µM aptamer: heating for 10 min at 95 °C followed by cooling at approximately 1 °C/min. The first MMP2 experiment was performed in an alternative system (PBS, pH 7.4, 5 mM MgCl_2_, 0.05% Tween 20), reproducing the conditions of a published study on the development of an ELASA assay [15].

### 4.5. Configuration I: Protein on the Biosensor, Aptamer in Solution

Experiments were performed on an Octet R8 (Sartorius) using Ni-NTA biosensors (18-5101). Biosensors were prehydrated for 10 min in running buffer. The target protein was immobilized via its histidine tag from a 2.5 µg/mL solution to a signal of 0.5 nm (for the in-house PCSK9 preparation, 5 µg/mL, continuous immobilization for 450 s, signal ~1 nm; for RAGE, without a threshold condition, signal up to 3 nm). The temperature was 30 °C, and the shaking speed 1000 rpm. Regeneration was performed with 10 mM glycine-HCl, pH 2.4, followed by restoration of the nickel coating with 10 mM NiSO_4_. Each aptamer was analyzed in a separate well at 10 and 1 µM (for some aptamers against ESM1, 10 and 3.33 µM). Immobilization stability was monitored with a reference biosensor bearing protein that was not immersed in the analyte solution.

### 4.6. Configuration II: Aptamer on the Biosensor, Protein in Solution

Biotinylated aptamers were immobilized on streptavidin SAX2 biosensors (18-5136) from a 25 nM solution to a response of about 0.5 nm; the concentration and target response were chosen following [24]. Biotin was attached to the 5′ end of each aptamer through a standard C6 amino linker, which places the biotin sufficiently far from the putative binding domain to minimize steric hindrance during interaction with the target protein. The protein was added in solution: MMP2 at 253.8, 84.6, and 28.2 nM, and the in-house PCSK9 preparation at 1000, 333.3, and 111.1 nM. No regeneration was applied. Each experiment included reference biosensors without immobilized aptamer, immersed in the same protein solutions.

### 4.7. Processing of Sensorgrams

Processing was performed in Octet BLI Analysis 12. In configuration I, the signal of a reference biosensor bearing immobilized protein but not in contact with the analyte was subtracted (drift correction). In configuration II, the averaged signal of reference biosensors without aptamer that had been in contact with the protein was subtracted (correction for nonspecific binding). In all cases, baseline alignment, inter-step correction to the dissociation phase, and Savitzky–Golay smoothing were applied. Fitting was carried out with a 1:1 model with full dissociation in local-fit mode (separately for each concentration) because the incomplete concentration series did not allow for a global fit. The series were limited to two or three concentrations by the screening design of the study (Section 3.2); criterion C5 (Section 4.8) was formulated specifically to compensate for the absence of a global fit in this regime.

### 4.8. A Priori Signal-Acceptance Criteria

The criteria were fixed before data analysis, drawing on the equipment manufacturer’s recommendations [25] and published aptamer protocols [24]:

**C1 (system validation).** Aptamer data for a target are interpreted only if a control antibody against the same target in the same experiment gave a concentration-dependent response with the features of high-affinity binding. Otherwise, the aptamer result is not interpreted at all: it is impossible to distinguish whether the protein preparation is inactive, the antibody is inactive, or binding is absent.

**C2 (response threshold).** A reference-subtracted response below 0.05 nm is not subject to constant calculation; 0.05–0.1 nm permits calculation only with an explicit caveat; reliable calculation requires 0.1–0.2 nm or above.

**C3 (fitting error).** A fit is rejected if the standard error of any of the parameters *K*_D_, *k*_on_, or *k*_off_ is comparable to, or exceeds, the parameter itself.

**C4 (concentration consistency of parameters).** Under local fitting, the *K*_D_, *k*_on_, and *k*_off_ values computed at different concentrations of the same pair must agree within a factor of 2–3. Systematic scatter is a sign that the model does not describe the data.

**C5 (shape of the concentration dependence).** The observed exponent *n* in the relationship *R* ∝ *C*^n^ must match the value implied by a 1:1 model with the obtained *K*_D_. A value of *n* close to unity across a concentration range that includes and exceeds *K*_D_ indicates the absence of saturation and points to nonspecific accumulation.

**C6 (independence from the target preparation).** When two or more independent preparations of the same target are available, the ranking of candidates by response must be reproducible. The absence of correlation indicates that the signal is determined by properties of the preparation rather than of the target.

The numerical thresholds used above (the 0.05/0.1–0.2 nm response bands in C2, the twofold-to-threefold parameter-agreement band in C4, and the requirement of at least a threefold concentration spacing) are heuristics specific to this instrument/analyte combination, set from the empirically observed baseline drift (≈0.01 nm, Section 2.3) with a safety margin, rather than universally validated, instrument-independent requirements. A quantitative, cross-instrument validation of these thresholds is a priority for future methodological work. The criteria themselves divide along the same line. C1, C3, C4 and C6 restate established biolayer-interferometry practice recommended by the instrument manufacturer and by published aptamer BLI protocols [24,25]; they were fixed before data analysis and were not adjusted to this dataset. C2 and C5, together with the numerical bands above, are study-specific decision rules, with C5 having been formulated for the minimal-data regime of this screen, and it is these rather than the established items that proved decisive in separating apparent from genuine binding here. They are accordingly presented as heuristics whose diagnostic value this dataset illustrates, not as validated, instrument-independent acceptance criteria.

### 4.9. Estimation of the Lower Bound of K_D_

To interpret the negative result quantitatively, we estimated the maximum theoretical response as *R*_max_ = *R*_load_ × (*M*_aptamer_/*M*_protein_) × *f*, where *R*_load_ is the response at the immobilization step, *M* denotes molecular masses, and *f* is an empirical correction factor. The molecular mass of the aptamer was taken as 16 kDa. The factor *f* = 2 was introduced as a conservative correction: control antibodies gave a response twice that predicted by this ratio (e.g., 2.19 nm for anti-MMP2 versus a calculated 1.04 nm), which we attribute to the bivalency of IgG and to packing density. Surface occupancy was calculated as θ = *R*_obs_/*R*_max_, and the lower bound of the constant as *K*_D_ ≥ *C*(1 − θ)/θ. The use of the maximum observed response (which knowingly includes a nonspecific component) and of a doubled *R*_max_ makes the estimate conservative, i.e., an underestimate.

### 4.10. Statistical Analysis

The consistency of candidate ranking across the two PCSK9 preparations was assessed with the Spearman rank correlation coefficient, with significance assessed via the exact permutation distribution of Spearman’s ρ for *n* = 10 (rather than the t-distribution approximation), with a 95% confidence interval for ρ obtained by the Fisher z-transformation. The exponent of the concentration dependence was calculated as *n* = ln(*R*_2_/*R*_1_)/ln(*C*_2_/*C*_1_) from the extreme points of the studied range. The value expected for a 1:1 model was calculated by the same formula for θ = *C*/(*C* + *K*_D_), with the *K*_D_ obtained from fitting. The relationship between predicted and experimental affinity was assessed with the same rank correlation over the 25 aptamers of the panel for which an experimental *K*_D_ could be computed at all (local 1:1 full fit, response ≥ 0.05 nm; one representative point per aptamer, taken at the concentration giving the strongest response), with significance derived from the t approximation, which is adequate at *n* = 25, and with the ESM1 subset and the PCSK9 subset analyzed separately as sensitivity checks. No confidence interval is reported for the exponent n: it is obtained from two or three concentrations without replicate measurements at each, so its sampling distribution cannot be estimated from these data. Standard errors for *K*_D_, k_on and k_off returned by the analysis software are tabulated for every curve in Supplementary Information S3.

## 5. Conclusions

We synthesized and tested 88 candidate aptamers against seven human proteins. The material was of good quality (purity 85–92%), and the analytical system was functional, as confirmed by antibody controls with *K*_D_ down to 10^−12^ M. Nevertheless, none of the molecules exhibited binding that satisfied the a priori acceptance criteria under the conditions tested; a conservative estimate limits the affinity to *K*_D_ ≥ 8.6 µM, at least 120-fold above the required 70 nM threshold.

We regard the methodological content of the work as more significant than the results for the specific molecules. We showed that two of the eight target-protein preparations tested with a control antibody proved unsuitable and, without an antibody control, would have yielded non-interpretable data; that replacement of the target preparation completely changes the ranking of candidates (ρ = −0.26), with the preparation carrying an additional tag systematically giving higher signals; and that kinetic fitting in local-fit mode returns consistent and plausible constants for data whose equilibrium behavior is incompatible with a 1:1 model. The last point constitutes the main practical result: the quality of the fit of an individual curve is insufficient, and verification of the shape of the amplitude’s concentration dependence is required.

We propose the controls listed in Table 6 as a reporting checklist for affinity data on computationally designed aptamers. They are a priori decision rules whose diagnostic value this dataset illustrates; the items marked as heuristics still require quantitative, instrument-independent validation before they can be treated as general acceptance criteria. Two gaps bound the negative result itself: neither an aptamer of independently confirmed affinity nor a scrambled-sequence oligonucleotide was carried through the same assay chain. The result therefore stands as a statement about these molecules under these conditions, not about biolayer interferometry or computational aptamer design in general, and closing those two gaps is the first task of the follow-up work that these data call for.

### 5.1. Study Limitations

1. A positive-control aptamer with independently confirmed affinity would have required titrating conditions specific to that aptamer; the conditions used here (including buffer and ionic composition) were supplied by the computational-design platform (Xelari, platform.xelari.com) together with the candidate sequences as those best suited to this panel, and titrating them for an external control was outside the scope of this work. The positive control actually used was an antibody; an aptamer with independently confirmed affinity did not pass through our chain (folding, buffer, assay scheme), so the influence of the folding regime and ionic composition on the binding of the entire class of molecules is not experimentally excluded.
2. An orthogonal method of affinity measurement (surface plasmon resonance, microscale thermophoresis, isothermal titration calorimetry) was not used. This bears directly on Section 2.6 and Figure 6: that the AVI-tagged preparation, rather than the target, drives the responses, and the rank inversion is the most parsimonious reading of those data, but it is not demonstrated. An orthogonal measurement of the ten PCSK9 aptamers against both preparations is the named priority for resolving it.
3. The identity of the synthesized compounds was confirmed by chromatographic purity and trityl-monitoring data, but not by mass spectrometry; the correctness of folding was not verified instrumentally.
4. We studied a single buffer system (except for the first MMP2 experiment in PBSMT), without screening of ionic composition and temperature. In particular, we did not examine the effect of an elevated K^+^ concentration (e.g., 100 mM), which could stabilize G-quadruplex structures if they were present; however, as noted in Section 4.1, in silico predictions revealed no quadruplex motifs in our sequences.
5. The concentration series were incomplete (2–3 points), which precluded a global fit. This reflects the screening purpose of the study, for which two to three concentrations spanning at least a threefold range are adequate: criterion C5 (Section 4.8) was designed specifically to distinguish saturable, specific binding from non-saturable, nonspecific accumulation from this minimal number of points. A full multi-point global fit remains an appropriate next step once a candidate has confirmed specific binding at the screening stage.
6. The candidate panel is not a set of 88 independent structural solutions: scaffold identifiers recur across targets and within individual panels.
7. The functional activity of the protein preparations was confirmed by antibody binding, i.e., the preservation of specific epitopes was demonstrated rather than that of the entire native structure.
8. We did not use a scrambled-sequence control oligonucleotide (of the same length and GC content) to assess nonspecific binding of protein to the oligonucleotide backbone. We therefore cannot quantitatively separate the specific and nonspecific components of the signal. All conclusions are based on comparisons with reference sensors (without ligand) and on verification of the shape of the concentration dependence, which allows rejection of non-saturable processes but does not give an absolute estimate of the contribution of nonspecific binding to the oligonucleotide.
9. The computational design pipeline that produced the panel is the intellectual property of the developer and was not disclosed to the authors, who received the final sequences and their predicted *K*_D_ values only. The experimental outcome therefore constrains the output of that pipeline as a whole but cannot be attributed to any particular stage within it, and we are not in a position to say which step of the prediction fails.

### 5.2. Data Excluded from Analysis

We excluded from the binding analysis: (1) all data for the MMP10 target, because the control antibody did not bind the protein preparation; (2) data for the PCSK9 (Elabscience) preparation, which bound neither of the two control antibodies; (3) data for the ESM1 target, for which no control antibody was used and, in addition, the predicted *K*_D_ (752–2819 µM) lay beyond the range of measurable values; (4) all *K*_D_, *k*_on_, and *k*_off_ values computed from curves with a response below 0.05 nm, which are present in the raw dataset but cannot serve as a basis for conclusions; (5) values with a parameter error exceeding the parameter itself; (6) data from the series with enhanced RAGE biosensor coverage (up to 3 nm), where we observed high heterogeneity of signal between biosensors, so these data were used only to estimate the upper bound of affinity and not for the calculation of constants.

## Figures and Tables

**Figure 1:**
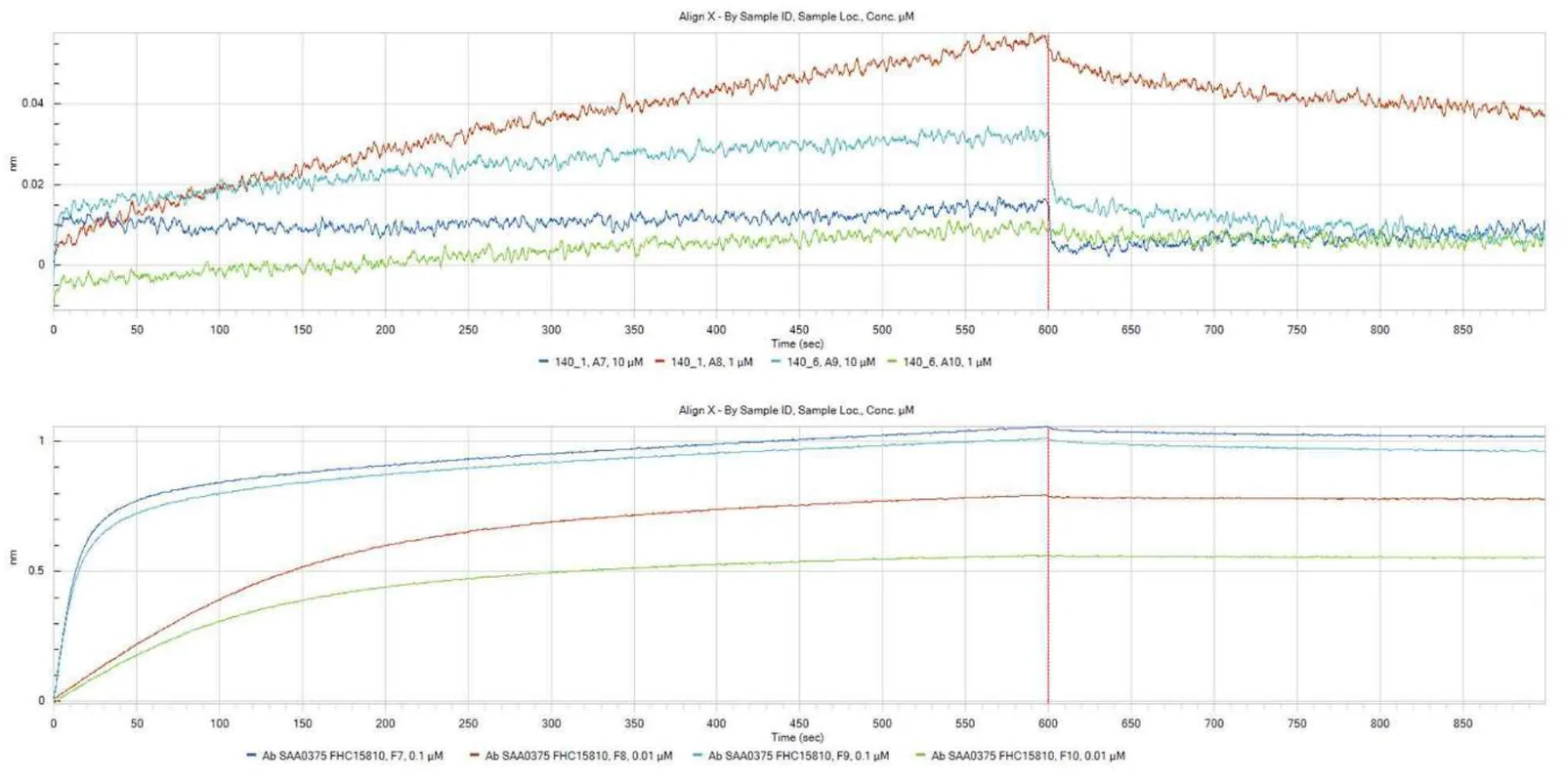
Sensorgrams for IL6 immobilized on a Ni-NTA biosensor. (**Top**) Aptamers 140_1 and 140_6 at 10 and 1 µM, ordinate scale 0–0.05 nm; (**bottom**) control antibody SAA0375 at 0.1 and 0.01 µM, scale 0–1 nm. The vertical dashed line separates the association and dissociation phases.

**Figure 2:**
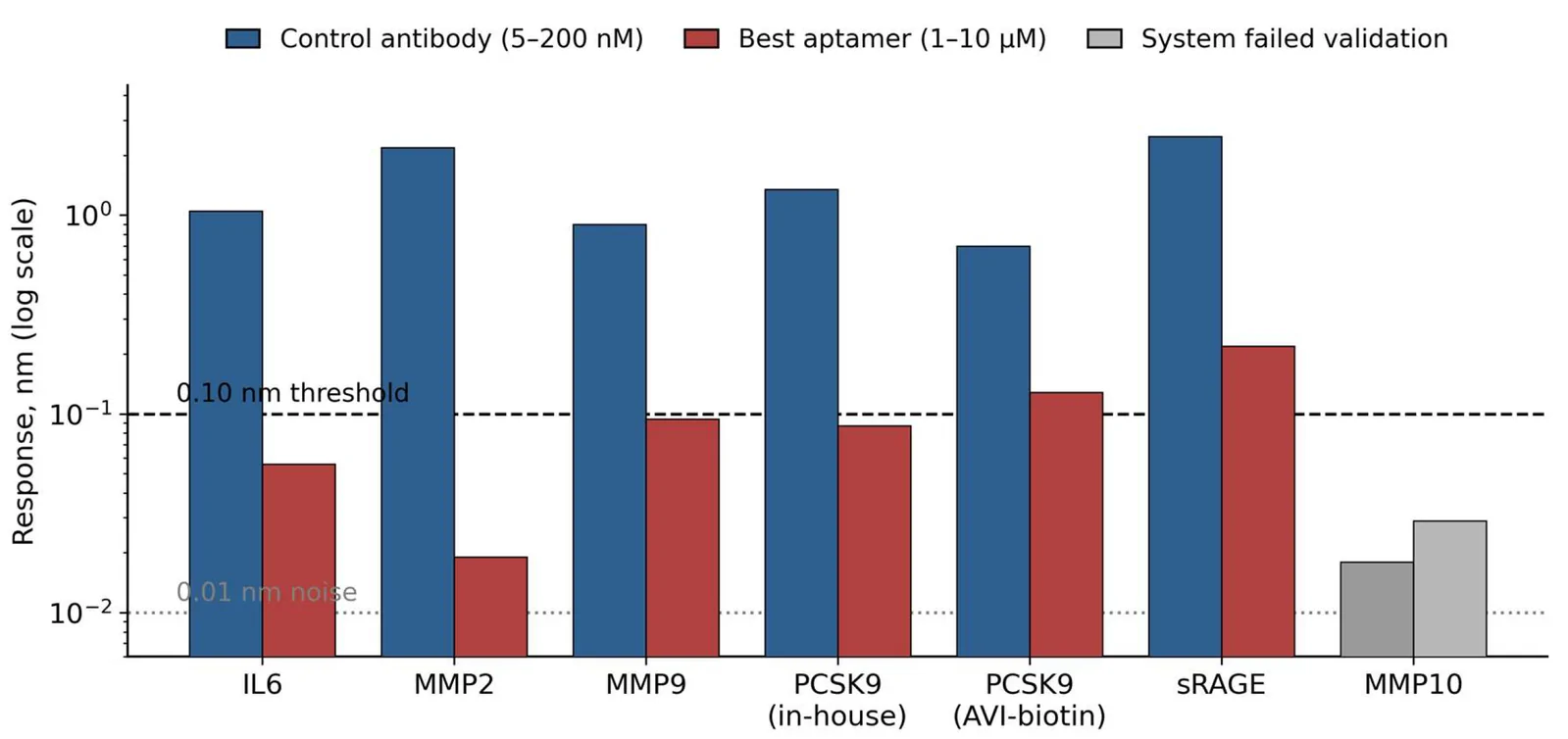
Maximum response of the best aptamer and the response of the control antibody for each target on a logarithmic scale. The dashed line marks the 0.10 nm threshold, and the dotted line the instrumental noise level of 0.01 nm. The MMP10 system, which failed antibody validation, is shown in gray.

**Figure 3:**
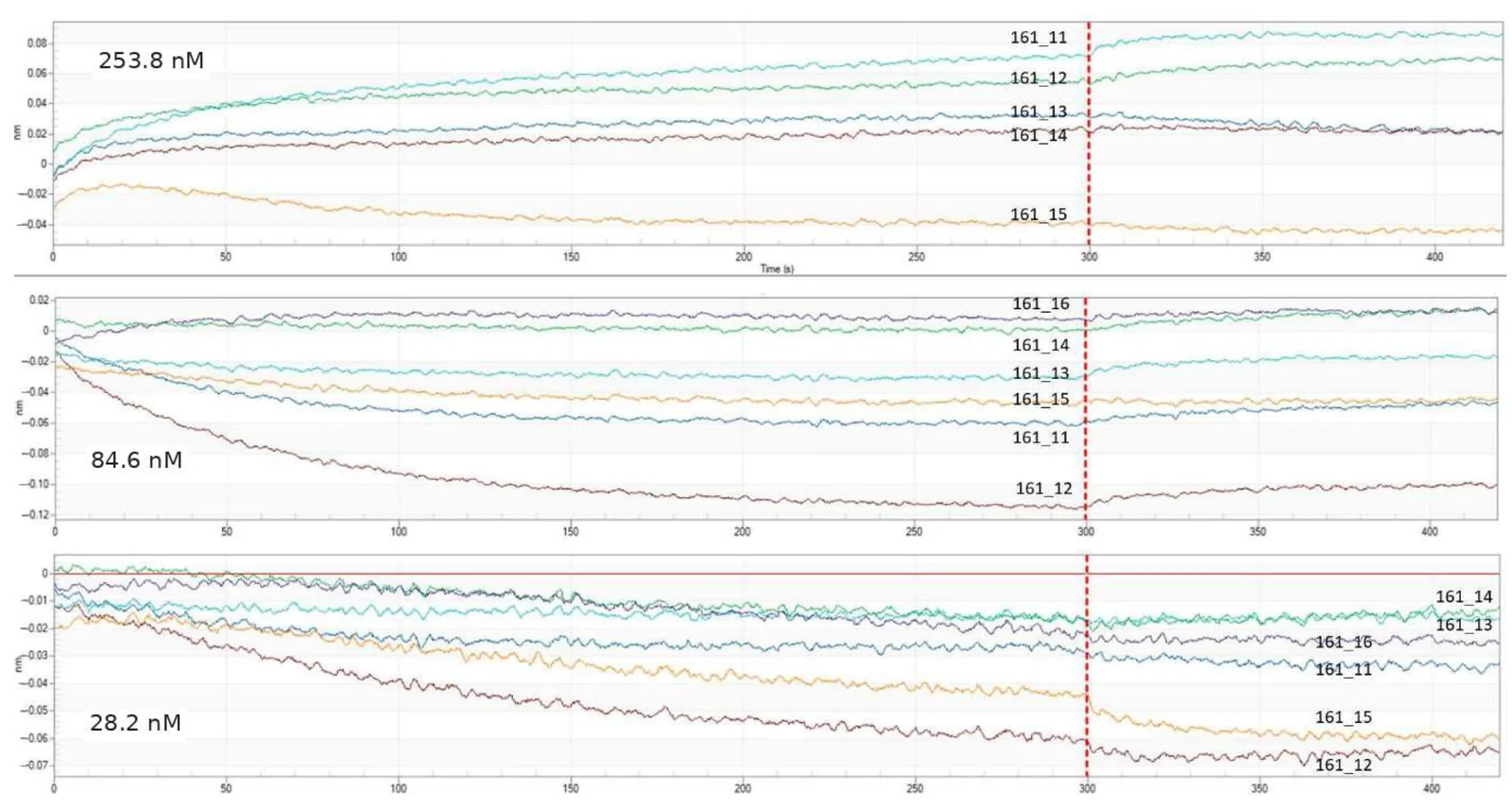
Configuration II, biotinylated aptamers 161_11−161_16 on SAX2 biosensors, MMP2 in solution at 253.8, 84.6, and 28.2 nM (**top**–**bottom**). Data are shown after subtraction of the signal from aptamer-free reference biosensors. At the two lower protein concentrations, the response for most aptamers is negative, i.e., sorption of protein on the aptamer-free biosensor exceeds sorption on the aptamer-bearing biosensor.

**Figure 4:**
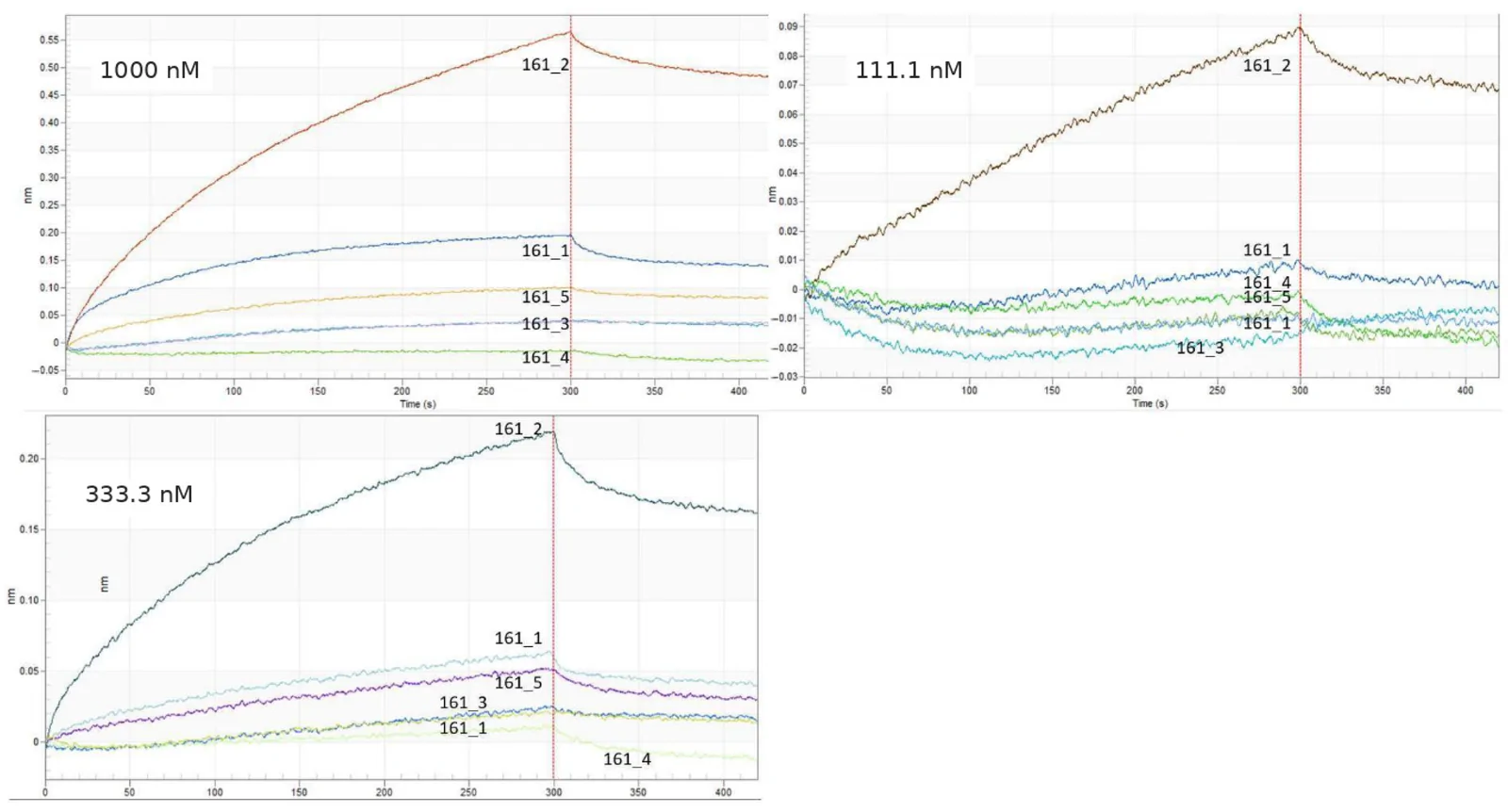
Configuration II, aptamers 161_1–161_5 on SAX2 biosensors, in-house PCSK9 preparation in solution at 1000, 333.3, and 111.1 nM. The curve for 161_2 does not reach a plateau within 300 s of association at any concentration.

**Figure 6:**
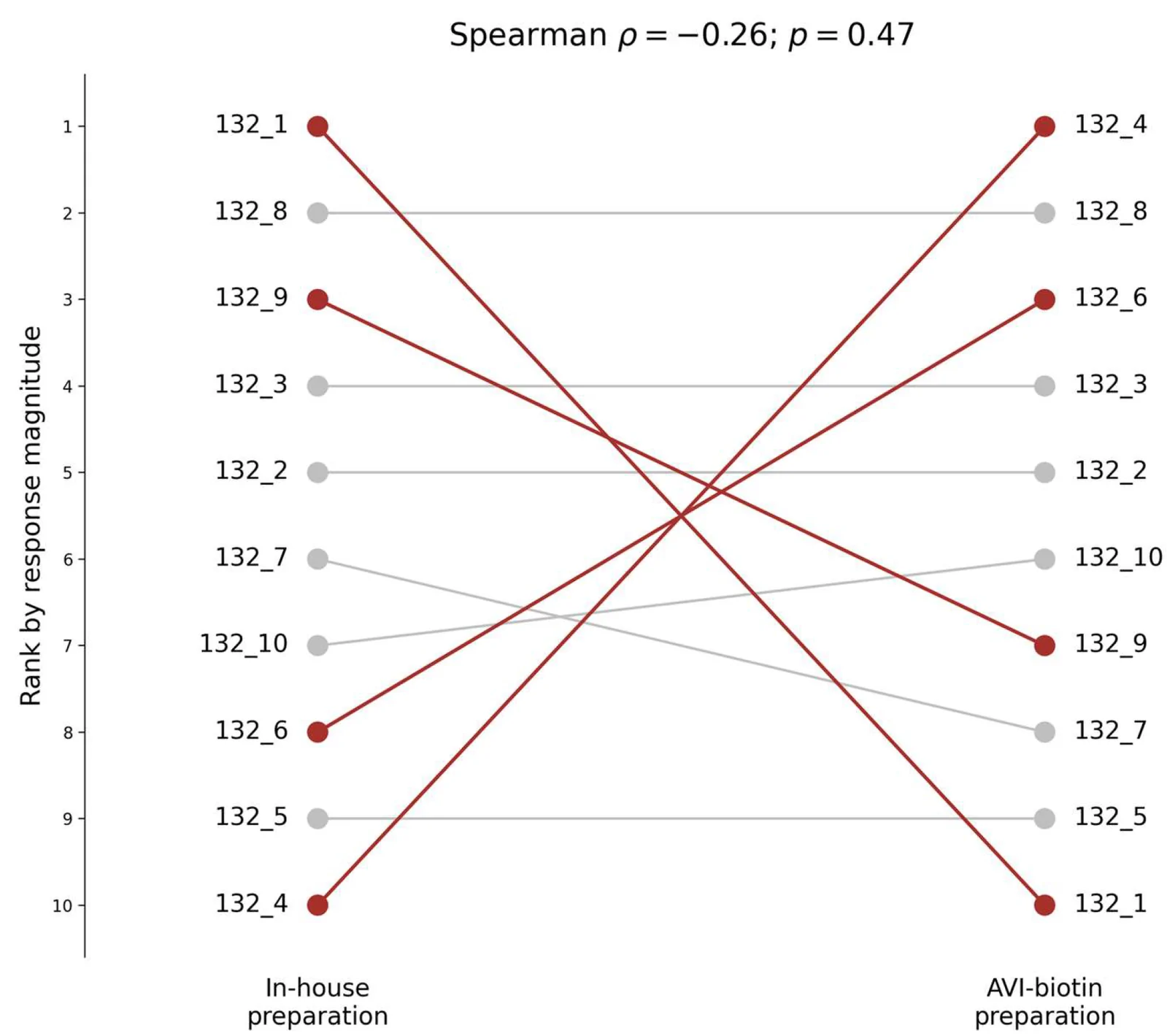
Ranks of ten aptamers against PCSK9 by response magnitude on two independent target preparations. Molecules shifting by four or more positions are highlighted in red.

**Table 1:** Synthesis quality control by target.

Target	Format	n	Mass, mg	Yield, % (Min–Max)	Purity, % (Min–Max, Median)
IL6	Non-biotinylated	10	0.362–0.625	85–89	85–90, 87
MMP2	Non-biotinylated	9	0.320–0.580	86–92	86–92, 88
MMP9	Non-biotinylated	10	0.340–0.562	86–91	85–91, 87
MMP10	Non-biotinylated	10	0.325–0.627	85–92	86–92, 88.5
ESM1	Non-biotinylated	10	0.321–0.606	85–91	85–92, 90
PCSK9	Non-biotinylated	10	0.422–0.778	85–92	86–92, 91
sRAGE	Non-biotinylated	10	0.324–0.626	85–91	87–92, 89.5
MMP2	5′-biotin	9	0.351–0.520	81–93	86–92, 88
PCSK9	5′-biotin	10	0.347–0.623	86–92	85–92, 89.5
Total		88	0.320–0.778	81–93 (med. 88)	85–92 (med. 89)

The structure of the panel warrants comment. Candidate identifiers recur across targets: identifier 5762 is assigned to both an aptamer against IL6 and one against sRAGE; 5757 to aptamers against MMP9 and ESM1; 1447 to aptamers against MMP9 and sRAGE; and 3855 to aptamers against IL6 and sRAGE. Within the MMP10 panel, identifier 3022 is assigned to two different compounds, and the sRAGE panel contains variants 5151 and 5151_2. This indicates that the candidates were generated from a common library of scaffolds with a variable region and that the number of independent structural solutions in the panel is fewer than 88. This limits interpretation: the panel does not constitute 88 independent tests of the hypothesis.

**Table 2:** Results of target-protein validation with control antibodies.

Target Preparation	Control Antibody	Concentration, nM	Response, nm	*K*_D_, M	Validation Outcome
IL6 (Cloud-Clone)	SAA0375	10–100	0.56–1.05	<1 × 10^−12^–3.1 × 10^−11^	Passed
MMP2 (AntibodySystem)	RHC32301	100	1.82–2.19	2.8 × 10^−9^–4.2 × 10^−9^	Passed
MMP9 (Cloud-Clone)	R3D69	5–50	0.069–0.90	2.0 × 10^−10^–3.2 × 10^−8^	Passed
PCSK9 (in-house)	evolocumab; RMJ24001	200	1.03–1.35	<1 × 10^−12^	Passed
PCSK9 (AVI-biotin)	evolocumab; RMJ24001	20–200	0.32–0.70	1.3 × 10^−9^–1.2 × 10^−8^	Passed
RAGE (Sino Biological)	VHH01903	10–100	1.42–2.49	<1 × 10^−12^–6.1 × 10^−10^	Passed
MMP10 (Sino Biological)	PHC39701	100–200	≤0.018	Not determined	Failed
PCSK9 (Elabscience)	evolocumab; RMJ24001	200	Absent	Not determined	Failed
ESM1	Not used	—	—	—	Not performed

Two preparations failed validation. For MMP10, neither the aptamers nor the control antibody, declared by the manufacturer as suitable for ELISA, immunohistochemistry, and Western blotting, gave a response above 0.018 nm despite correct immobilization of the protein on the biosensor. Such a result does not allow one to establish whether the protein preparation is inactive, the antibody is inactive, or aptamer binding is absent; the three hypotheses are indistinguishable with a single antibody control. For the PCSK9 (Elabscience) preparation, no binding was observed with either of the two independent antibodies, including the therapeutic one, which is direct evidence that the preparation did not retain the native conformation of its epitopes. For ESM1, a control antibody was not included in the scheme, so the system for this target is not validated at all.

**Table 3:** Maximum aptamer responses in validated systems and the conservative estimate of the lower bound of *K*_D_.

Target	Config.	Analyte *C*	Max Response, nm	Estimated *R*_max_, nm	θ	Lower Bound of *K*_D_	Fold Above 70 nM Threshold
IL6	I	10 µM	0.043	0.76	0.056	≥1.7 × 10^−4^ M	≈2400
MMP2	I	1 µM	0.019	0.22	0.086	≥1.1 × 10^−5^ M	≈150
MMP9	I	10 µM	0.094	0.17	0.54	≥8.6 × 10^−6^ M	≈120
PCSK9 (in-house)	I	10 µM	0.087	0.43	0.20	≥4.0 × 10^−5^ M	≈570
sRAGE	I	10 µM	0.219	2.67	0.082	≥1.1 × 10^−4^ M	≈1600

The estimates in Table 3 are deliberately conservative: we took the largest observed response, including one that is knowingly nonspecific, and doubled the theoretical *R*_max_. Even under these assumptions, for no validated target can the constant be below 8.6 µM, so the requirement *K*_D_ ≤ 70 nM is not met by a margin of at least two orders of magnitude, and the discrepancy with the predicted values reaches five orders of magnitude.

**Table 5:** Responses of the same aptamers against PCSK9 (10 µM) on two target preparations.

Aptamer	Response, In-House Preparation, nm	Rank	Response, AVI-Biotin Preparation, nm	Rank
132_1	0.0865	1	0.0105	10
132_8	0.0632	2	0.1106	2
132_9	0.0597	3	0.0725	7
132_3	0.0581	4	0.0927	4
132_2	0.0510	5	0.0839	5
132_7	0.0452	6	0.0436	8
132_10	0.0432	7	0.0809	6
132_6	0.0323	8	0.0994	3
132_5	0.0227	9	0.0310	9
132_4	0.0105	10	0.1280	1

**Table 6:** Minimal set of controls and criteria for BLI testing of aptamers.

No.	Control or Criterion	What It Eliminates	Established Practice or Heuristic
1	Control antibody (preferably two independent) against the same target in the same experiment	Inactive protein preparation; inactive antibody; immobilization error	Established
2	Positive-control aptamer with published affinity	Suppression of binding by the folding regime, buffer ionic composition, or assay scheme for the entire class of molecules	Established; not performed here
3	Reference biosensor: with ligand but without analyte (configuration I); without ligand but with analyte (configuration II)	Baseline drift; nonspecific surface sorption	Established
4	Analyte-free buffer sensorgram for noise assessment	Mistaking baseline fluctuations for signal	Established
5	Response threshold of 0.1 nm (0.05 nm with a caveat)	Curves lying in the noise region	Heuristic (this study)
6	Rejection of fits whose parameter error is comparable to the parameter	Pseudo-precise constants	Established
7	Agreement of *K*_D_, *k*_on_, and *k*_off_ across concentrations within a factor of 2–3	Inadequacy of the model to the data	Established
8	Verification of the exponent of the response–concentration dependence	Non-saturable processes mimicking binding	Heuristic (this study)
9	Two independent target preparations; reproducibility of ranking	Binding to a tag, carrier, or impurity	Established
10	Two orthogonal immobilization configurations	Artifacts of orientation and site shielding	Established
11	Scrambled-sequence control on the biosensor (configuration II)	Sorption of protein on an oligonucleotide of any sequence	Established; not performed here

Items 2 and 11 were not performed in our study, and it is precisely these that form the main limitations of the work. Item 9 was performed by chance, owing to the need to replace an unsuitable protein preparation, and it yielded one of the most informative results, which leads us to recommend it as mandatory rather than optional.

## Data Availability

Complete sets of sensorgrams and the full table of response values, fitted *K*_D_, k_on, and k_off for every tested pair and concentration, are provided as Supplementary Information S2 (Figures S1–S12) and Supplementary Information S3 (Tables S3–S12) with this submission, together with Supplementary Information S1 (Tables S1 and S2) (aptamer sequences and predicted *K*_D_ values) and Supplementary Information S4 (Figure S13) (raw vs. reference-subtracted sensorgrams), superseding the previous upon-request-only arrangement. The candidate sequences are released in full in Supplementary Information S1 (Tables S1 and S2). The computational design algorithm that generated them remains the intellectual property of the developer of the computational method and was not disclosed to the authors.

## Supplementary Materials

The following supporting information can be downloaded at https://www.mdpi.com/article/10.3390/ijms27198469/s1.
