# Supplementary material for: Validation of Aptamer–Protein Interactions by Biolayer Interferometry: A Study of 88 Candidate Molecules
Source: Int J Mol Sci. 2026 Sep 23;27(19):8469. doi: 10.3390/ijms27198469 (PMC13664908; doi:10.3390/ijms27198469)
Supplement: Supplementary file 1 [file ijms-27-08469-s001.zip › ijms-4568549-supplementary.pdf]

Supplementary Materials

Validation of Aptamer–Protein Interactions by Biolayer Interferometry: A Study of 88 Candidate Molecules

Elizaveta S. Ershova, Natalia V. Pozdnyakova, Aleksandra Yu. Egorshina, Elena V., Yakushenko, Igor V. Dobrokhotov, Nadezhda P. Lyamina, Bella B. Gagulaeva and Alexey Moskalev

This file combines, under one cover, the four items of supporting information referred to in the main text: Supplementary Information S1 (aptamer sequences and predicted K_D), Supplementary Information S2 (sensorgrams obtained during BLI analysis), Supplementary Information S3 (quantitative characteristics of aptamer–target protein interactions from BLI analysis), and Supplementary Information S4 (raw vs. reference-subtracted sensorgrams). Each item below is kept as its own section for traceability to the original files; within each section, every figure and table is captioned according to the MDPI convention (“Figure S1: title”, “Table S1: title”), numbered continuously through the whole document.

Contents

Section	Numbering used	Content
Supplementary Information S1	Table S1, Table S2	Aptamer sequences and predicted (in silico) vs. experimental K_D
Supplementary Information S2	Figure S1–S12	Sensorgrams obtained during BLI analysis
Supplementary Information S3	Table S3–S12	Quantitative characteristics of aptamer–target protein interactions from BLI analysis
Supplementary Information S4	Figure S13	Raw (pre-reference-subtraction) and reference-subtracted sensorgrams for representative pairs

Supplementary Information S1

Aptamer sequences and predicted K_D

Table S1: Summary table compiled from all Xelari summary.pdf files received: SBT number, target, sequence, predicted (in silico) K_D, and experimental BLI data (Table S3–S12: NTA sensors for non-biotinylated, SAX2 for biotinylated aptamers). The response shown is at the concentration giving the strongest signal; the experimental K_D is given only if it could be formally computed at all (local fit 1:1 full, response ≥ 0.05 nm); otherwise – 'n/a'.

SBT No.	Target	Sequence (5'->3')	Predicted K_D (in silico), M	Sensor	Best response, nm (conditions)	Experimental K_D, M	Note
140_40	ESM1	CCCGAGGCGGTTGGCCCACCAGGGTGGGCGGTTCCCACGGG	7.52E-04	NTA	0.0809 nm @ 3.33 µM	2.56E-06	
140_41	ESM1	CAGTCGAGTGCTCTGCAATCAGTTTACTGACGACTG	8.24E-04	NTA	0.0846 nm @ 3.33 µM	8.44E-06	
140_42	ESM1	CCGACGCCGGCATCTAGAGCAGATCCTAGCGATGCCATTCGTCGG	1.26E-03	NTA	0.0924 nm @ 3.33 µM	5.00E-06	
140_43	ESM1	CTCCGCGGGAGCCTGAAGGCAGAGACCCCCGCACACGGAG	1.63E-03	NTA	0.0797 nm @ 10 µM	5.01E-05	
140_44	ESM1	GGGTGAAAACGTGACCAGCAAGAAACTGGTCCCAACGAACCC	2.15E-03	NTA	0.0206 nm @ 3.33 µM	n/a	response < 0.05 nm
140_45	ESM1	GTGCAAGGCCACGGTAGGGGCTGACGAGCATCGAACCGGTACGTGCAC	2.19E-03	NTA	0.1236 nm @ 10 µM	1.03E-05	
140_46	ESM1	GGTCTGGGCAAGCGTCGCCAGATGCAAACTGGCTGTTCACTGCCACC	2.39E-03	NTA	0.0547 nm @ 10 µM	1.99E-03	
140_47	ESM1	TAGAAGCCCGGCCTTTGATAAGGCCGGGAGCTACGGGAACCAC	2.42E-03	NTA	0.1201 nm @ 10 µM	1.43E-05	
140_48	ESM1	GGGCTCGGAGCGGTCGGAAAGGAGACCCTCCGGCCC	2.57E-03	NTA	-0.0475 nm @ 10 µM	n/a	response < 0.05 nm
140_49	ESM1	CCCGACATTACGCCAGGAGGCATCGTGGTCTACCGGG	2.82E-03	NTA	0.0746 nm @ 10 µM	5.72E-04	
140_1	IL6	GAGTCACGGTCGGGCGTGCGGCCCGCGCGCGTACTC	5.45E-11	NTA	0.0561 nm @ 1 µM	5.97E-07	
140_2	IL6	GTGCGGGGAGATCGCAAAGGGAGAGCGCTCACGCAC	8.66E-11	NTA	0.0263 nm @ 1 µM	n/a	response < 0.05 nm
140_3	IL6	TTGCCTGTGAGTTGGGAGCACCCTAACGCAAGGCAA	9.64E-11	NTA	0.0339 nm @ 1 µM	n/a	response < 0.05 nm
140_4	IL6	CCACCGCGGTGGTAGGAGATGCACGTTAAGACGGTGG	1.02E-10	NTA	0.0479 nm @ 1 µM	n/a	response < 0.05 nm
140_5	IL6	GTCTTGCTACTGCCGAGAGGACGGCACACCCCCAAGAC	1.40E-10	NTA	0.02 nm @ 1 µM	n/a	response < 0.05 nm
140_6	IL6	TACCACCGTAAGGTGCATAAGCAACCTTAAGGCGGTA	1.46E-10	NTA	0.0321 nm @ 10 µM	n/a	response < 0.05 nm
140_7	IL6	TAGTCCCGCCGTACCCAAGAAAGGTCGGCCCATCCTA	1.53E-10	NTA	0.0194 nm @ 10 µM	n/a	response < 0.05 nm
140_8	IL6	CCACAGGCCGTCGCGTAAGACGCTCGCTACGCCTGTTGG	1.76E-10	NTA	0.0428 nm @ 10 µM	n/a	response < 0.05 nm
140_9	IL6	GCCGCGAGAAGAGCCTTAGAGCTGAAAGGCGGACAGCGGC	1.83E-10	NTA	0.0312 nm @ 10 µM	n/a	response < 0.05 nm
140_10	IL6	GTGCAAACTGGGATGGCAATCCCGAAACAGTCGCAC	2.18E-10	NTA	0.0136 nm @ 1 µM	n/a	response < 0.05 nm
140_30	MMP10	GCCCGACGCATGCGCCTGTCACACCAGGCTTCAACTGCGTCATTGGC	3.12E-11		no BLI data	n/a	
140_31	MMP10	CCGTATGGTGGAACGGGCGGGCGAAAACACCCCGG	7.72E-11	NTA	0.0147 nm @ 1 µM	n/a	response < 0.05 nm
140_32	MMP10	CCGTTCTAGGACGGCGTAAGGCCGAATCCTCCACGG	9.95E-11	NTA	0.0176 nm @ 1 µM	n/a	response < 0.05 nm
140_33	MMP10	AGGGAGCAAAGCAATAGTGCAGGTGCCGGCCCCCATCCGGGAATCCCT	1.71E-10	NTA	0.0135 nm @ 1 µM	n/a	response < 0.05 nm
140_34	MMP10	AGCTTGTGGCAACTGAGGAACCAGTGAGCCCCAAGCT	1.91E-10	NTA	0.0197 nm @ 1 µM	n/a	response < 0.05 nm
140_35	MMP10	GCTGCCGTACGACCACAAACCGACGTCGTCTGCCCATCAGC	2.40E-10		no BLI data	n/a	
140_36	MMP10	CGATCTGGCGACCAGGGAGCCTGGACACGCCCGCTCG	5.49E-10	NTA	0.0289 nm @ 10 µM	n/a	response < 0.05 nm
140_37	MMP10	TCGCGGGTAAGCACGTTCGGACAAAGGTCCGAACTCCCGCGA	8.81E-10	NTA	0.0193 nm @ 10 µM	n/a	response < 0.05 nm
140_38	MMP10	TCTCTCGGAAGCCGACCCTGGTGGCGGCCCACCCCAGCTTCCGTAAGA	2.41E-09	NTA	0.0269 nm @ 10 µM	n/a	response < 0.05 nm
140_39	MMP10	CCGTATGGTGGAACGGGGAACCGAAAACACCCCGG	3.44E-09	NTA	0.0164 nm @ 10 µM	n/a	response < 0.05 nm

SBT No.	Target	Sequence (5'->3')	Predicted K_D (in silico), M	Sensor	Best response, nm (conditions)	Experimental K_D, M	Note
140_11	MMP2	TGTTCAGCCTGGCCGACTGGGGAGTGACGGCCCGATGCGAACA	7.60E-11	NTA	0.0132 nm @ 1000 nM	n/a	response < 0.05 nm
140_12	MMP2	CGGCTGGCAACTGTGGGGGGTAATGGTACCCCCAGTCCTGCCG	1.15E-10	NTA	0.0114 nm @ 100 nM	n/a	response < 0.05 nm
140_13	MMP2	GAAACGACCCCAGCGCCTCCCGCGCATCGGGCTTGACAGCACAAGTAGTTTC	4.18E-10	NTA	0.0185 nm @ 1000 nM	n/a	response < 0.05 nm
140_14	MMP2	CCGAGGTCTAACAGAGTCCGTGCGACAACGGACTCAGAATGTCGG	5.96E-10		no BLI data	n/a	
140_15	MMP2	CGTCTCCGGGGAACGAGGAGGATCGTCCCGTGACG	1.18E-09	NTA	0.019 nm @ 1000 nM	n/a	response < 0.05 nm
140_16	MMP2	CCGAAGGAAGGTCTCGACTACGCTCGAGGACCATACTCCTACGG	5.59E-08	NTA	0.018 nm @ 1000 nM	n/a	response < 0.05 nm
140_17	MMP2	GGCACGACAGCCTCTCGGACGGGAACTCCAGTAAGGCGAGTCTTGCC	6.26E-08	NTA	0.0105 nm @ 1000 nM	n/a	response < 0.05 nm
140_18	MMP2	CACGCTTGGCAGGCTAGGGCGCCCGCCCGACCAGCGTG	8.06E-08	NTA	0.0087 nm @ 1000 nM	n/a	response < 0.05 nm
140_19	MMP2	GAGTGCTTAGGTTGCAGTGCGTGAACTCGCACAGCACCTAAAAACCCACTC	1.21E-07	NTA	0.0147 nm @ 1000 nM	n/a	response < 0.05 nm
161_11	MMP2	TGTTCAGCCTGGCCGACTGGGGAGTGACGGCCCGATGCGAACA	7.60E-11	SAX2	0.0324 nm @ 253.8 nM	n/a	response < 0.05 nm
161_12	MMP2	CGGCTGGCAACTGTGGGGGGTAATGGTACCCCCAGTCCTGCCG	1.15E-10	SAX2	0.0225 nm @ 253.8 nM	n/a	response < 0.05 nm
161_13	MMP2	GAAACGACCCCAGCGCCTCCCGCGCATCGGGCTTGACAGCACAAGTAGTTTC	4.18E-10	SAX2	0.0716 nm @ 253.8 nM	n/a	response >= 0.05 nm, but K_D was not calculated (see explanation in article/report)
161_14	MMP2	CCGAGGTCTAACAGAGTCCGTGCGACAACGGACTCAGAATGTCGG	5.96E-10	SAX2	0.054 nm @ 253.8 nM	n/a	response >= 0.05 nm, but K_D was not calculated (see explanation in article/report)
161_15	MMP2	CGTCTCCGGGGAACGAGGAGGATCGTCCCGTGACG	1.18E-09	SAX2	-0.0394 nm @ 253.8 nM	n/a	response < 0.05 nm
161_16	MMP2	CCGAAGGAAGGTCTCGACTACGCTCGAGGACCATACTCCTACGG	5.59E-08	SAX2	0.0073 nm @ 84.6 nM	n/a	response < 0.05 nm
161_17	MMP2	GGCACGACAGCCTCTCGGACGGGAACTCCAGTAAGGCGAGTCTTGCC	6.26E-08	SAX2	-0.0457 nm @ 253.8 nM	n/a	response < 0.05 nm
161_18	MMP2	CACGCTTGGCAGGCTAGGGCGCCCGCCCGACCAGCGTG	8.06E-08	SAX2	-0.0418 nm @ 253.8 nM	n/a	response < 0.05 nm
161_19	MMP2	GAGTGCTTAGGTTGCAGTGCGTGAACTCGCACAGCACCTAAAAACCCACTC	1.21E-07	SAX2	0.0082 nm @ 253.8 nM	n/a	response < 0.05 nm
140_20	MMP9	GGCAGGCACGCGTGAGATACATCAAGATGACCCCTTCGTCTCTGCC	3.27E-11	NTA	0.0455 nm @ 10 µM	n/a	response < 0.05 nm
140_21	MMP9	AGCGCTCGTACGGTAGTACTCGGCGTACCACTACGCGTGGGGGCT	5.91E-11	NTA	0.0714 nm @ 10 µM	2.35E-08	
140_22	MMP9	TAGCTGTCGTGCTCGCAAAGGTGCGCACGAACCGAAGCTA	6.32E-11	NTA	0.0937 nm @ 10 µM	3.61E-06	
140_23	MMP9	CCCGGAGCGCGTTGGCCCACGAGGGTGGGCGGTCCCACGGG	1.28E-10	NTA	0.0635 nm @ 10 µM	2.71E-08	
140_24	MMP9	CGGGCACCAGTAGATGCCAACTTCGCAATGGGTACCG	1.58E-10	NTA	0.0242 nm @ 10 µM	n/a	response < 0.05 nm
140_25	MMP9	AACACCTCGGTGCGGGTCAACACTCGAGTTAGGACCCCCCGAGGTCGGGTT	1.80E-10	NTA	-0.0035 nm @ 10 µM	n/a	response < 0.05 nm
140_26	MMP9	CGGGGGCGTTTCTAGGTGGGGTAGGGCCACCATGTGTACGCCCCG	4.59E-10	NTA	-0.0097 nm @ 10 µM	n/a	response < 0.05 nm
140_27	MMP9	GCTTGGAGCGATACGGTAAGTTCCCCAGGAACTAGCTCGAATCGGCTCCCAGC	5.43E-10	NTA	-0.0175 nm @ 10 µM	n/a	response < 0.05 nm
140_28	MMP9	GAAGTGGACAACTCAAGACCGGGAGGTCGAGGATGGCCACTTC	1.95E-09	NTA	-0.0142 nm @ 10 µM	n/a	response < 0.05 nm
140_29	MMP9	CGTCTTTGAGGAGGCGCCGATTTTCCCAAGAAAACGAGCGGCACCTAAGAAGACG	3.02E-09	NTA	0.0074 nm @ 10 µM	n/a	response < 0.05 nm
132_1	PCSK9	GGGCTCTCTACTGAGCGAATAATAGACGCGTCAGTCAGCCC	2.97E-08	NTA	0.0865 nm @ 1.00E+04 nM	5.98E-06	
132_2	PCSK9	ACACTTCCGCTCTAGTTCCAGGCTCAGTTACGCCGCACAACTAGAGCGTGTGTGT	5.07E-08	NTA	0.0839 nm @ 1.00E+04 nM	1.26E-03	
132_3	PCSK9	AGCAGGTAATCTCAGCGTCAACGCCAGATTACGGTGCT	6.10E-08	NTA	0.0927 nm @ 1.00E+04 nM	2.97E-03	
132_4	PCSK9	CGGGCCATCGCAGGCCAGGAAGGCTGCGATGGACCG	7.79E-08	NTA	0.128 nm @ 1.00E+04 nM	2.35E-03	
132_5	PCSK9	TGACCGGGAGCTCGCCCCGCCAACGGGGCCCGTGCA	1.01E-07	NTA	0.031 nm @ 1.00E+04 nM	n/a	response < 0.05 nm

SBT No.	Target	Sequence (5'->3')	Predicted K_D (in silico), M	Sensor	Best response, nm (conditions)	Experimental K_D, M	Note
132_6	PCSK9	CTAACGAAGTCCGATGGGTAGCCCAGGGGTGACGGGCGCTAG	2.55E-07	NTA	0.0994 nm @ 1.00E+04 nM	3.80E-04	
132_7	PCSK9	TGCATCGCCATCGAGTGCGTGGTGTTGTGACCACGCACCATGGCTTTAGCA	2.58E-07	NTA	0.0452 nm @ 1.00E+04 nM	n/a	response < 0.05 nm
132_8	PCSK9	GACACAGAGCGCTGACGAAACGTCTCCGCTGCCGGTGTCCTGCT	2.81E-07	NTA	0.1106 nm @ 1.00E+04 nM	3.57E-04	
132_9	PCSK9	CGCGAGCTGTAGGTTGAAAGGGGAATCAGACCTTAACGCGCG	3.13E-07	NTA	0.0725 nm @ 1.00E+04 nM	2.08E-03	
132_10	PCSK9	GCGCTCAAGTGGTATTCCACTGGGAAGTGGCACATAGCCCGC	5.06E-07	NTA	0.0809 nm @ 1.00E+04 nM	2.96E-04	
161_1	PCSK9	GGGCTCTCTACTGAGCGAATAATAGACGCGTCAGTCAGCCC	2.97E-08	SAX2	0.1947 nm @ 1000 nM	2.35E-07	
161_2	PCSK9	ACACTTCCGCTCTAGTTCCAGGCTCAGTTACGCCGCACAACTAGAGCGTGTG TGT	5.07E-08	SAX2	0.5601 nm @ 1000 nM	2.02E-07	
161_3	PCSK9	AGCAGGTAATCTCAGCGTCAACGCCAGATTACGGTGCT	6.10E-08	SAX2	0.0386 nm @ 1000 nM	n/a	response < 0.05 nm
161_4	PCSK9	CGGGCCATCGCAGGCCAGGAAGGCTGCGATGGACCG	7.79E-08	NTA	0.0105 nm @ 333.3 nM	n/a	response < 0.05 nm
161_5	PCSK9	TGACCGGGAGCTCGCCCCGCCAACGGGGCCCGTGCA	1.01E-07	SAX2	0.0996 nm @ 1000 nM	2.41E-07	
161_6	PCSK9	CTAACGAAGTCCGATGGGTAGCCCAGGGGTGACGGGCGCTAG	2.55E-07	NTA	-0.0294 nm @ 111.1 nM	n/a	response < 0.05 nm
161_7	PCSK9	TGCATCGCCATCGAGTGCGTGGTGTTGTGACCACGCACCATGGCTTTAGCA	2.58E-07	SAX2	0.0582 nm @ 333.3 nM	4.10E-07	
161_8	PCSK9	GACACAGAGCGCTGACGAAACGTCTCCGCTGCCGGTGTCCTGCT	2.81E-07	NTA	0.0323 nm @ 333.3 nM	n/a	response < 0.05 nm
161_9	PCSK9	CGCGAGCTGTAGGTTGAAAGGGGAATCAGACCTTAACGCGCG	3.13E-07	NTA	0.0381 nm @ 333.3 nM	n/a	response < 0.05 nm
161_10	PCSK9	GCGCTCAAGTGGTATTCCACTGGGAAGTGGCACATAGCCCGC	5.06E-07	SAX2	0.0409 nm @ 333.3 nM	n/a	response < 0.05 nm
140_50	sRAGE	TAGTCCCGCCGTACCCGCTAGCGGTCGGCCCATCCTA	9.06E-10	NTA	-0.0552 nm @ 1.00E+04 nM	n/a	response < 0.05 nm
140_51	sRAGE	TGGGGTGCGAGGCATACGCTCGTTTTGGCACCCCA	2.53E-09	NTA	-0.0047 nm @ 1.00E+04 nM	n/a	response < 0.05 nm
140_52	sRAGE	GCTACGTTCGATATCCAGCACGGTAAAAGGCGTGATCGATACGATAATAGC	4.26E-09	NTA	-0.0903 nm @ 1.00E+04 nM	n/a	response < 0.05 nm
140_53	sRAGE	TGCCCTGACGCGATGTGACAGACAACGGCGGAGCCGCTGAAGTCCAAGGG CA	4.99E-09	NTA	-0.0036 nm @ 1.00E+04 nM	n/a	response < 0.05 nm
140_54	sRAGE	TGCCCTGACGCGATGTGGGTGACAACGCTGAAACGCTGAAGTCCAAGGGC A	5.19E-09	NTA	0.2194 nm @ 1.00E+04 nM	3.86E-07	
140_55	sRAGE	TTGCCTGTGAGTTGGGCCGGCCCTAACGCAAGGCA	6.13E-09	NTA	-0.0656 nm @ 1000 nM	n/a	response < 0.05 nm
140_56	sRAGE	GTGGGGGCGATGCAGCTTTCACTGCCATTTCGACGCCAGCCCCAC	6.68E-09	NTA	-0.0807 nm @ 1000 nM	n/a	response < 0.05 nm
140_57	sRAGE	CGCCGTGAAAGTCCCGTGCCGGTGCAATGAGGAGCCTACAGGCG	7.04E-09	NTA	-0.1156 nm @ 1000 nM	n/a	response < 0.05 nm
140_58	sRAGE	GTGGCTGCCCTATCCGACATGGGAATCCCCAGCGCCGGATACCAC	8.73E-09	NTA	-0.1026 nm @ 1000 nM	n/a	response < 0.05 nm
140_59	sRAGE	TAGCTGTCGTGCTCGAACTCTGCGCACGAACCGAAGCTA	2.76E-08	NTA	-0.0325 nm @ 1.00E+05 nM	n/a	response < 0.05 nm

Table S2: Correlation of predicted (in silico, Xelari) and experimental (BLI, strongest signal) K_D for 25 aptamers for which an experimental K_D could be computed at all (out of 88 in the panel; for the rest the response was below the 0.05 nm threshold and K_D was not calculated – they are excluded from this subset, which introduces a systematic selection bias toward stronger signal).

Spearman ρ = 0.497, p = 0.0115, n = 25

SBT No.	Target	Sequence (5'->3')	Predicted K_D, M	Experimental K_D, M
140_40	ESM1	CCCGAGGCGGTTGGCCCACCAGGGTGGGCGGTTCCCACGGG	7.52E-04	2.56E-06
140_41	ESM1	CAGTCGAGTGCTCTGCAATCAGTTTACTGACGACTG	8.24E-04	8.44E-06
140_42	ESM1	CCGACGCCGGCATCTAGAGCAGATCCTAGCGATGCCATTCGTCGG	1.26E-03	5.00E-06
140_43	ESM1	CTCCGCGGGAGCCTGAAGGCAGAGACCCCCGCACACGGAG	1.63E-03	5.01E-05
140_45	ESM1	GTGCAAGGCCACGGTAGGGGCTGACGAGCATCGAACCGGTACGTGCAC	2.19E-03	1.03E-05
140_46	ESM1	GGTCTGGGCAAGCGTCGCCAGATGCAAACTGGCTGTTCACTGCCACC	2.39E-03	1.99E-03
140_47	ESM1	TAGAAGCCCGGCCTTTGATAAGGCCGGGAGCTACGGGAACCAC	2.42E-03	1.43E-05
140_49	ESM1	CCCGACATTACGCCAGGAGGCATCGTGGTCTACCGGG	2.82E-03	5.72E-04
140_1	IL6	GAGTCACGGTCGGGCGTGCGGCCCGCGCGCGTACTC	5.45E-11	5.97E-07
140_21	MMP9	AGCGCTCGTACGGTAGTACTCGGCGTACCACTACGCGTGGGGGCT	5.91E-11	2.35E-08
140_22	MMP9	TAGCTGTCGTGCTCGCAAAGGTGCGCACGAACCGAAGCTA	6.32E-11	3.61E-06
140_23	MMP9	CCCGGAGCGCGTTGGCCCACGAGGGTGGGCGGTCCCACGGG	1.28E-10	2.71E-08
132_1	PCSK9	GGGCTCTCTACTGAGCGAATAATAGACGCGTCAGTCAGCCC	2.97E-08	5.98E-06
161_1	PCSK9	GGGCTCTCTACTGAGCGAATAATAGACGCGTCAGTCAGCCC	2.97E-08	2.35E-07
132_2	PCSK9	ACACTTCCGCTCTAGTTCCAGGCTCAGTTACGCCGCACAACTAGAGCGTGTGTGT	5.07E-08	1.26E-03
161_2	PCSK9	ACACTTCCGCTCTAGTTCCAGGCTCAGTTACGCCGCACAACTAGAGCGTGTGTGT	5.07E-08	2.02E-07
132_3	PCSK9	AGCAGGTAATCTCAGCGTCAACGCCAGATTACGGTGCT	6.10E-08	2.97E-03
132_4	PCSK9	CGGGCCATCGCAGGCCAGGAAGGCTGCGATGGACCG	7.79E-08	2.35E-03
161_5	PCSK9	TGACCGGGAGCTCGCCCCGCCAACGGGGCCCGTGCA	1.01E-07	2.41E-07
132_6	PCSK9	CTAACGAAGTCCGATGGGTAGCCCAGGGGTGACGGGCGCTAG	2.55E-07	3.80E-04
161_7	PCSK9	TGCATCGCCATCGAGTGCGTGGTGTTGTGACCACGCACCATGGCTTTAGCA	2.58E-07	4.10E-07
132_8	PCSK9	GACACAGAGCGCTGACGAAACGTCTCCGCTGCCGGTGTCCTGCT	2.81E-07	3.57E-04
132_9	PCSK9	CGCGAGCTGTAGGTTGAAAGGGGAATCAGACCTTAACGCGCG	3.13E-07	2.08E-03
132_10	PCSK9	GCGCTCAAGTGGTATTCCACTGGGAAGTGGCACATAGCCCGC	5.06E-07	2.96E-04
140_54	sRAGE	TGCCCTGACGCGATGTGGGTGACAACGCTGAAACGCTGAAGTCCAAGGGCA	5.19E-09	3.86E-07

Supplementary Information S2

Sensorgrams obtained during BLI analysis

Performing BLI analysis on NTA sensors

This section presents the sensorgrams obtained when analyzing the interaction of target proteins immobilized on NTA sensors with analytes in solution (Figures S1–S8) and, further below, on SAX2 sensors (Figures S9–S12). Sensorgrams are given for all tested aptamer–protein pairs and at all tested concentrations. Detailed information on the assay method, aptamer solutions, and control antibodies is given in the main text of the report and in Supplement M.

Note that the sensorgrams are shown at different scales; the instrumental response in nm is indicated on the Y axis. For some sensorgrams the analyte concentration is given in exponential format, 1E4 nM corresponds to 10,000 nM.

1. Analysis on NTA sensors, affinity determination of aptamers against IL6.

The sensorgrams in Figure S1 are shown after subtraction of the reference-sensor signal (a sensor loaded with the target protein but not exposed to aptamers or other analytes) and depict 3 stages of the experiment: baseline establishment, analyte association with the ligand on the sensor, and subsequent analyte dissociation. Stages are separated from one another by vertical dashed lines. The aptamer code or antibody name, well number on the plate during the run, and the tested analyte concentration are indicated.

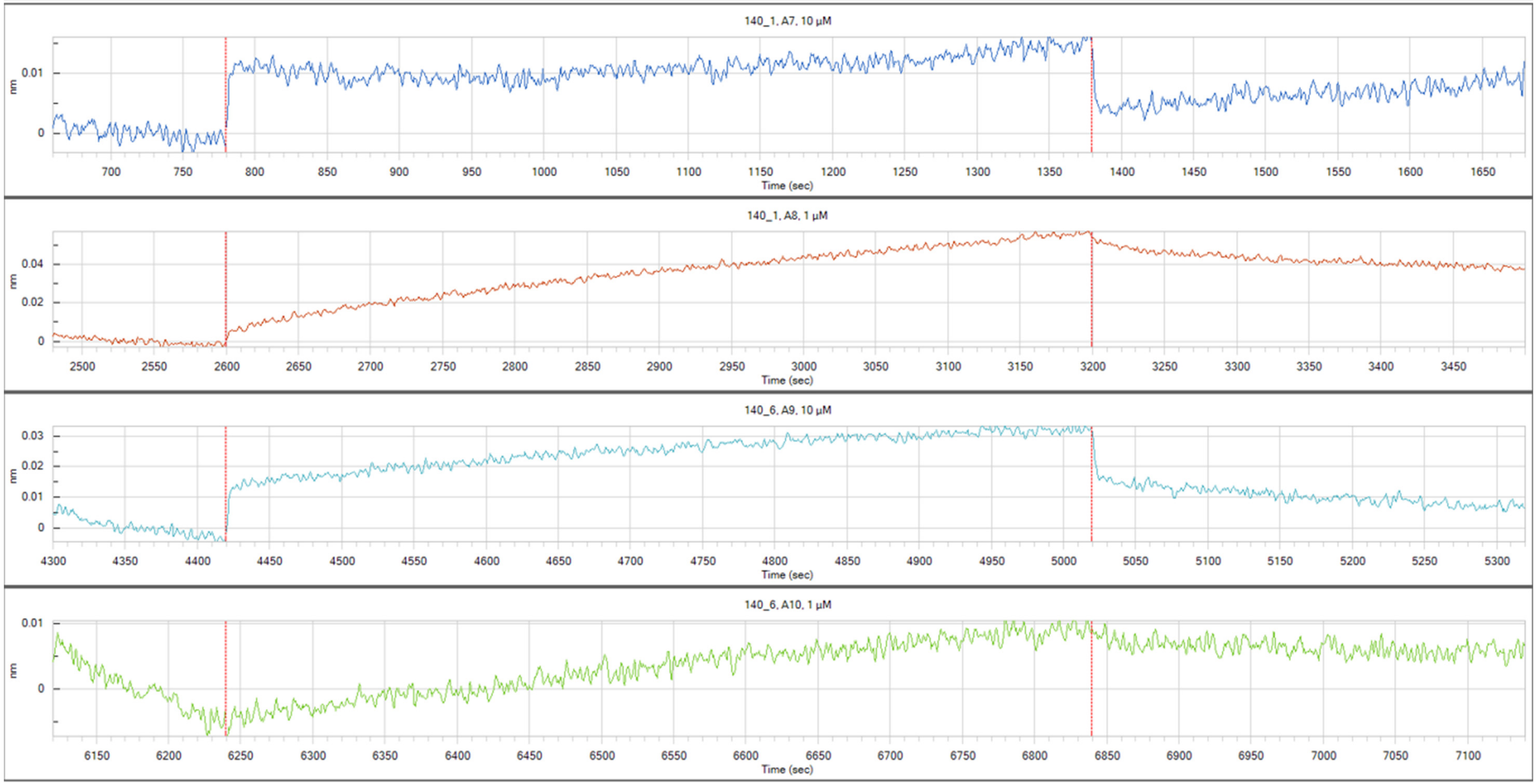
140_1, A7, 10 µM
nm
0.01
0
700 750 800 850 900 950 1000 1050 1100 1150 1200 1250 1300 1350 1400 1450 1500 1550 1600 1650
Time (sec)
140_1, A8, 1 µM
nm
0.04
0.02
0
2500 2550 2600 2650 2700 2750 2800 2850 2900 2950 3000 3050 3100 3150 3200 3250 3300 3350 3400 3450
Time (sec)
140_6, A9, 10 µM
nm
0.03
0.02
0.01
0
4300 4350 4400 4450 4500 4550 4600 4650 4700 4750 4800 4850 4900 4950 5000 5050 5100 5150 5200 5250 5300
Time (sec)
140_6, A10, 1 µM
nm
0.01
0
6150 6200 6250 6300 6350 6400 6450 6500 6550 6600 6650 6700 6750 6800 6850 6900 6950 7000 7050 7100
Time (sec)

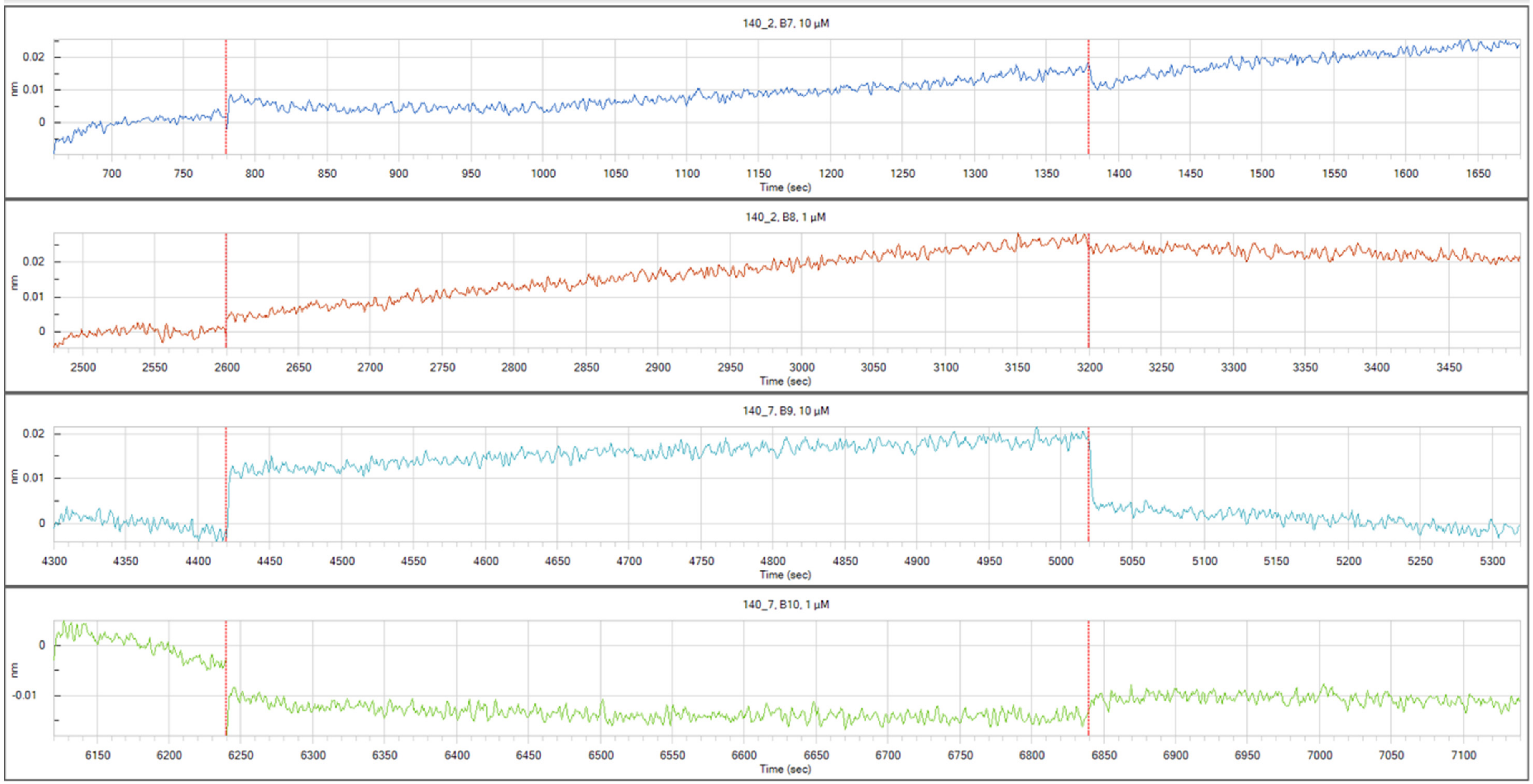
140_2, B7, 10 µM
140_2, B8, 1 µM
140_7, B9, 10 µM
140_7, B10, 1 µM
Time (sec)
nm

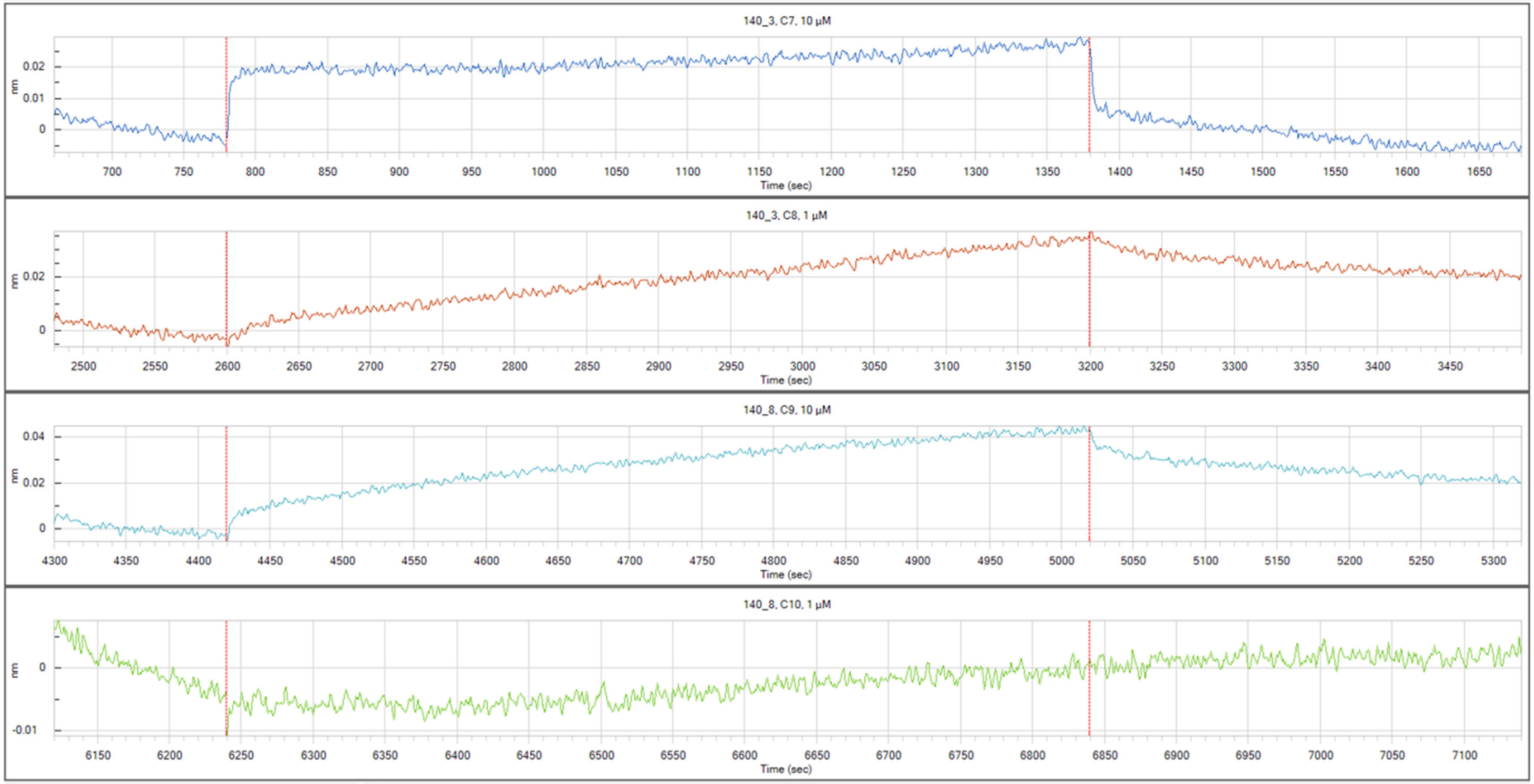
140_3, C7, 10 µM
nm
0.02
0.01
0
700 750 800 850 900 950 1000 1050 1100 1150 1200 1250 1300 1350 1400 1450 1500 1550 1600 1650
Time (sec)
140_3, C8, 1 µM
nm
0.02
0
2500 2550 2600 2650 2700 2750 2800 2850 2900 2950 3000 3050 3100 3150 3200 3250 3300 3350 3400 3450
Time (sec)
140_8, C9, 10 µM
nm
0.04
0.02
0
4300 4350 4400 4450 4500 4550 4600 4650 4700 4750 4800 4850 4900 4950 5000 5050 5100 5150 5200 5250 5300
Time (sec)
140_8, C10, 1 µM
nm
0
-0.01
6150 6200 6250 6300 6350 6400 6450 6500 6550 6600 6650 6700 6750 6800 6850 6900 6950 7000 7050 7100
Time (sec)

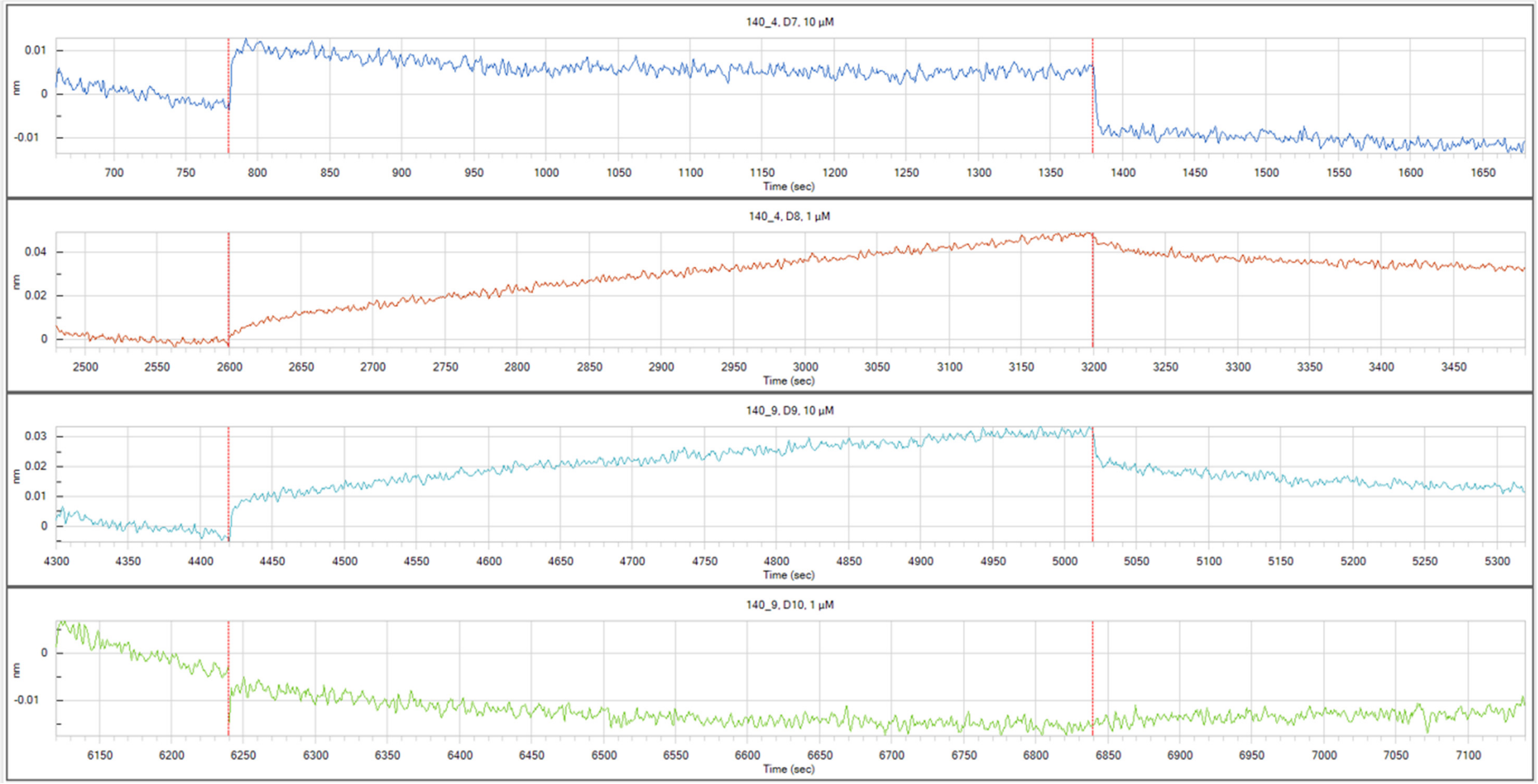
140_4, D7, 10 μM
140_4, D8, 1 μM
140_9, D9, 10 μM
140_9, D10, 1 μM
nm
Time (sec)

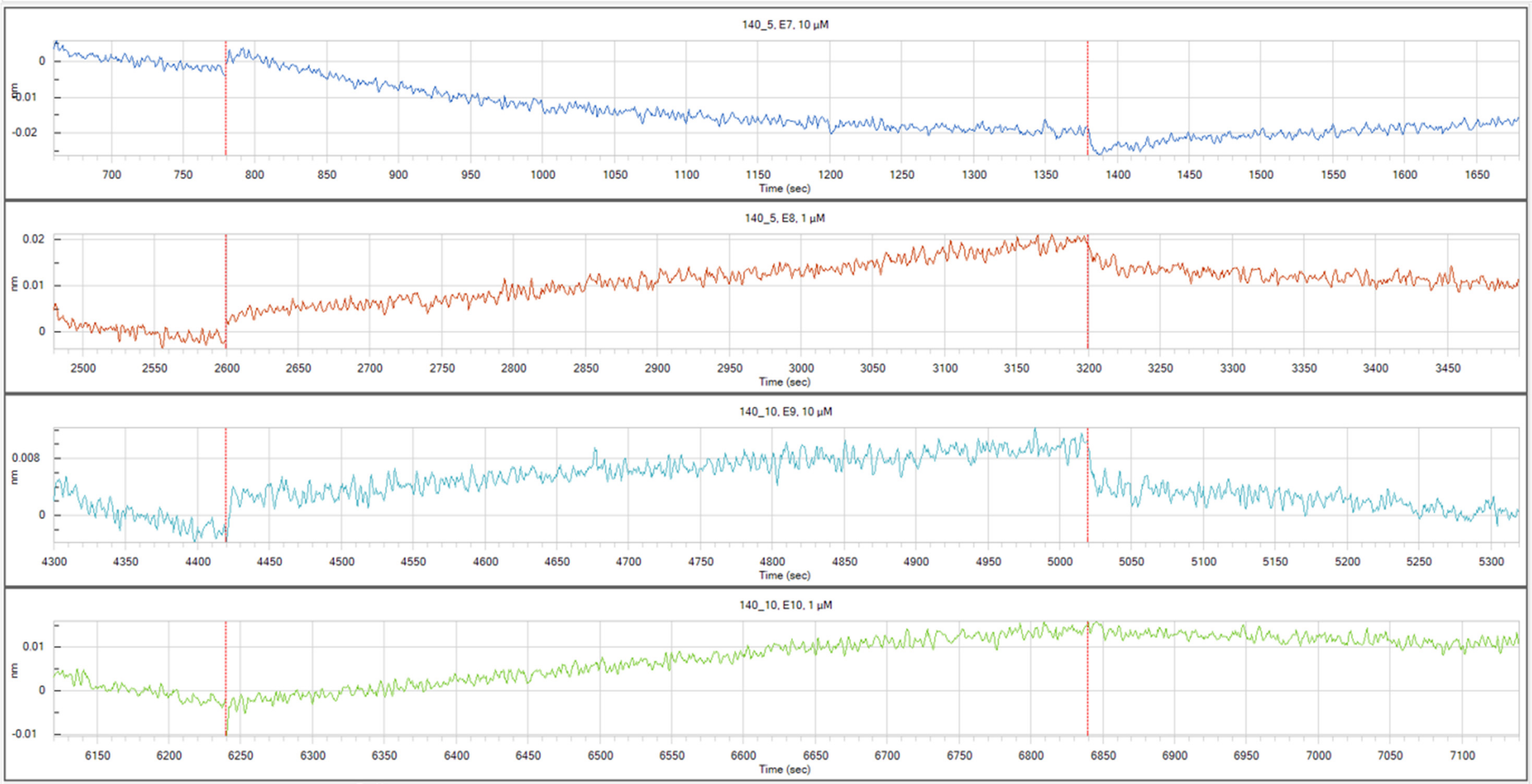

140_5, E7, 10 µM
140_5, E8, 1 µM
140_10, E9, 10 µM
140_10, E10, 1 µM
nm
Time (sec)

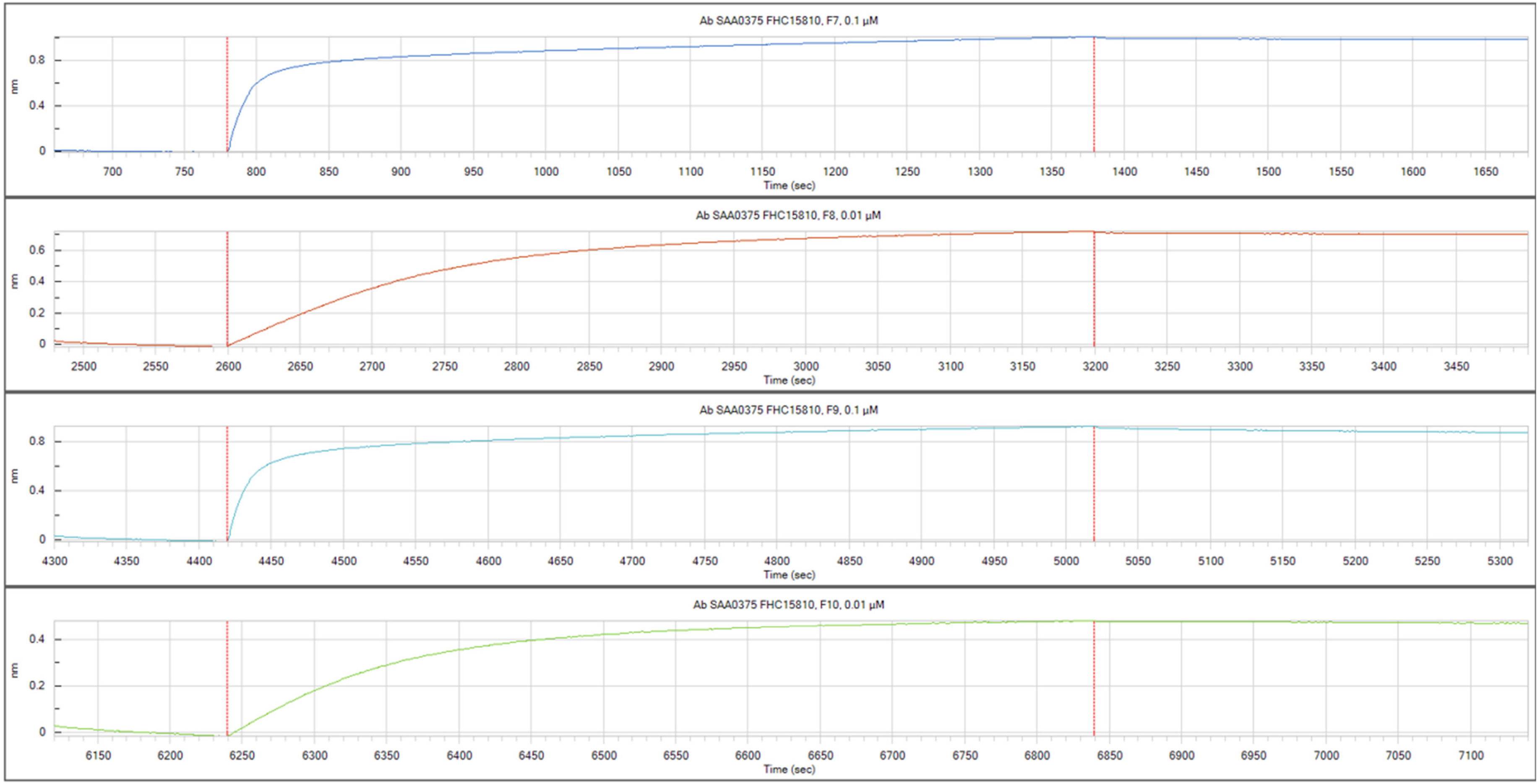

Figure S1: Sensorgrams obtained from analysis on NTA sensors for affinity determination of aptamers against IL6

2. Analysis on NTA sensors, affinity determination of aptamers against MMP2.

The sensorgrams in Figure S2 are shown after subtraction of the reference-sensor signal and depict 3 stages of the experiment: baseline establishment, analyte association, and subsequent dissociation, separated by vertical dashed lines. The aptamer code or antibody name, well number, and tested analyte concentration are indicated. Sensorgrams are shown at different scales; the response in nm is indicated on the Y axis. For comparison, sensorgrams obtained for the control antibody are shown, and, to illustrate the noise level in the study, sensorgrams for an analyte-free buffer solution are also shown (PBSMT).

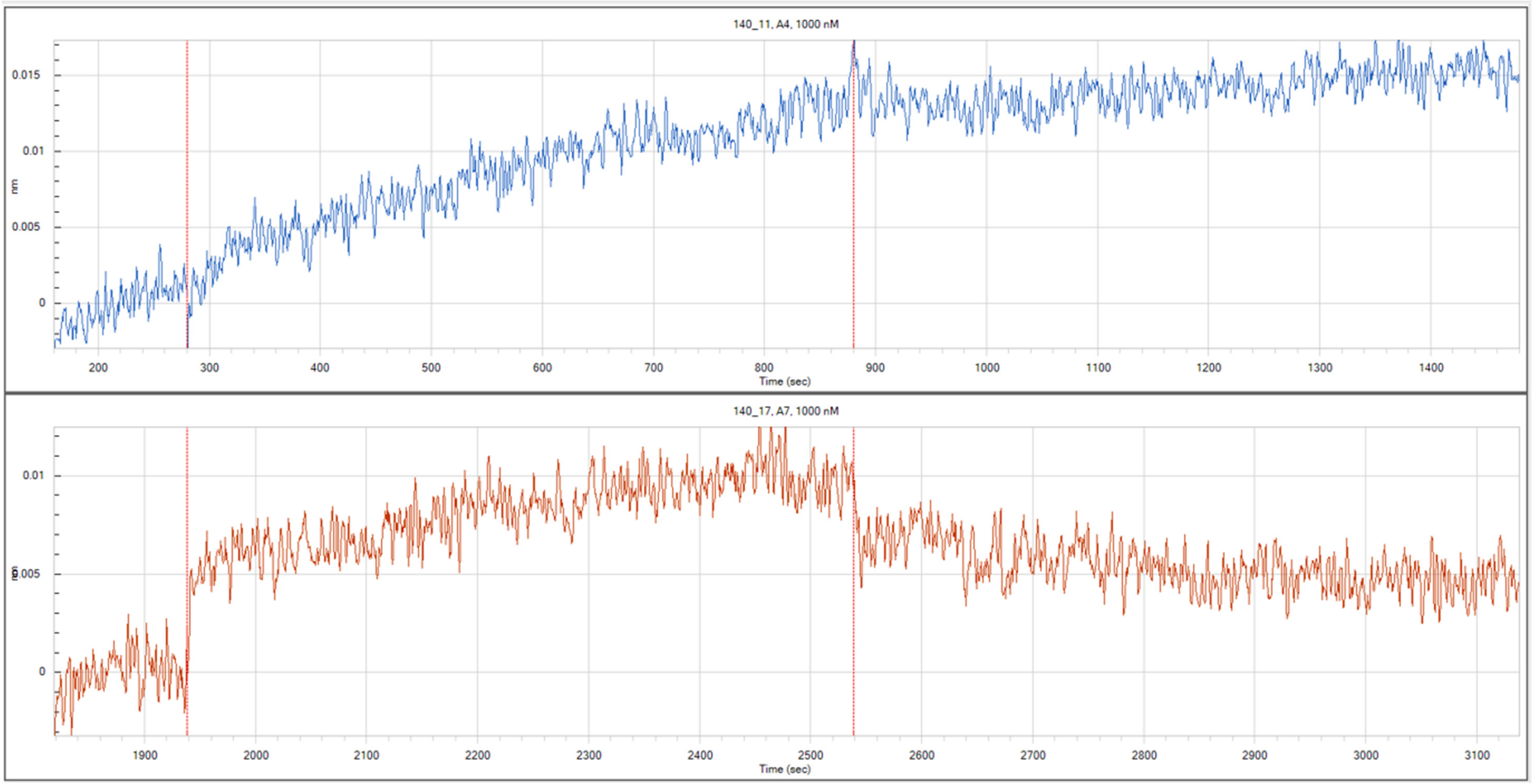

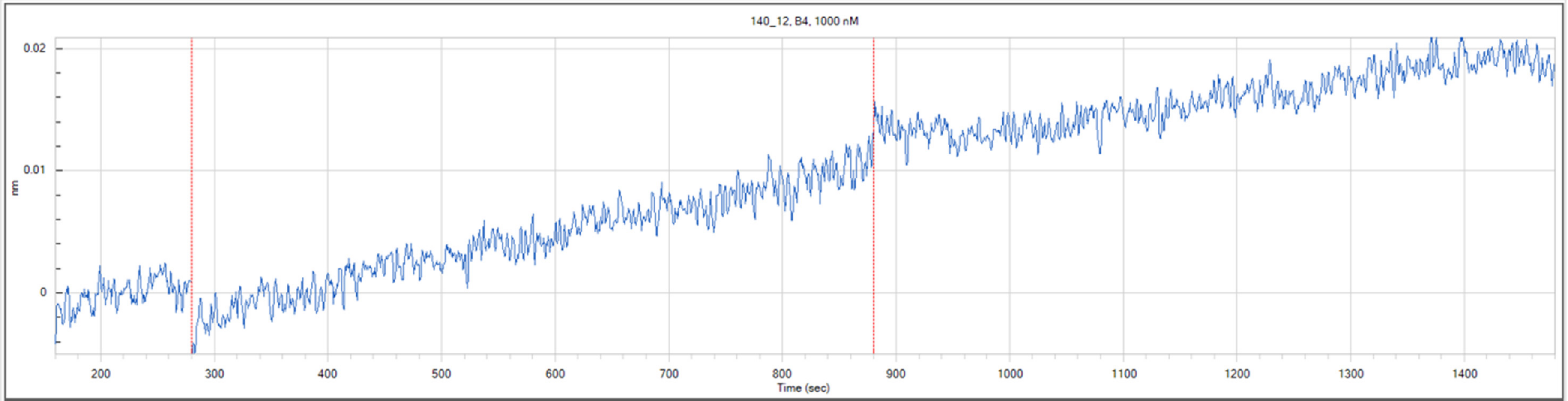
140_12, B4, 1000 nM
0.02
0.01
0
nm
200
300
400
500
600
700
800
900
1000
1100
1200
1300
1400
Time (sec)

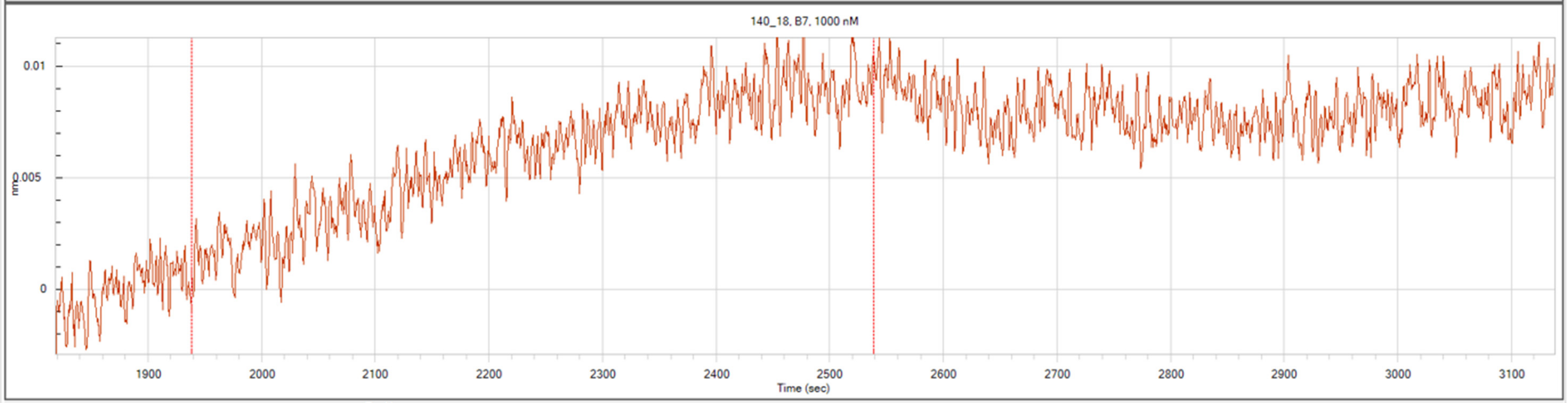
140_18, B7, 1000 nM
0.01
0.005
0
nm
1900
2000
2100
2200
2300
2400
2500
2600
2700
2800
2900
3000
3100
Time (sec)

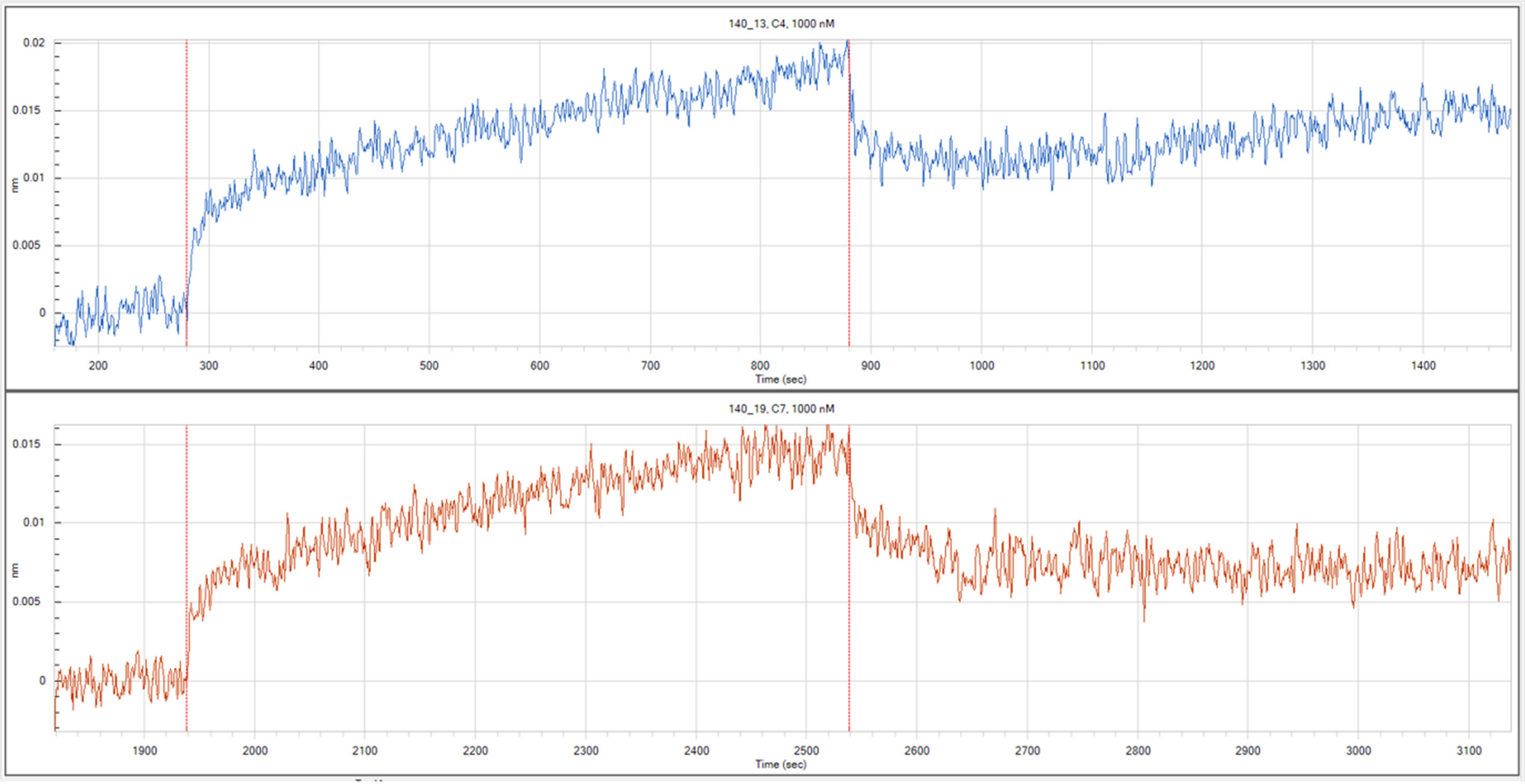
140_13, C4, 1000 nM
0.02
0.015
0.01
0.005
0
nm
200
300
400
500
600
700
800
900
1000
1100
1200
1300
1400
Time (sec)
140_19, C7, 1000 nM
0.015
0.01
0.005
0
nm
1900
2000
2100
2200
2300
2400
2500
2600
2700
2800
2900
3000
3100
Time (sec)

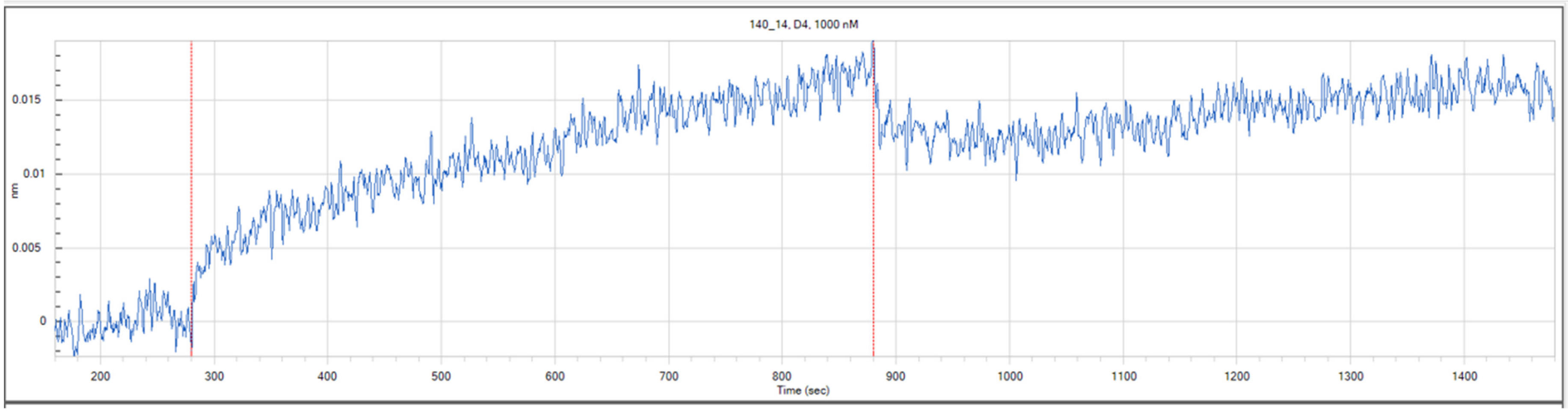

140_14, D4, 1000 nM
0.015
0.01
0.005
0
nm
200
300
400
500
600
700
800
900
1000
1100
1200
1300
1400
Time (sec)

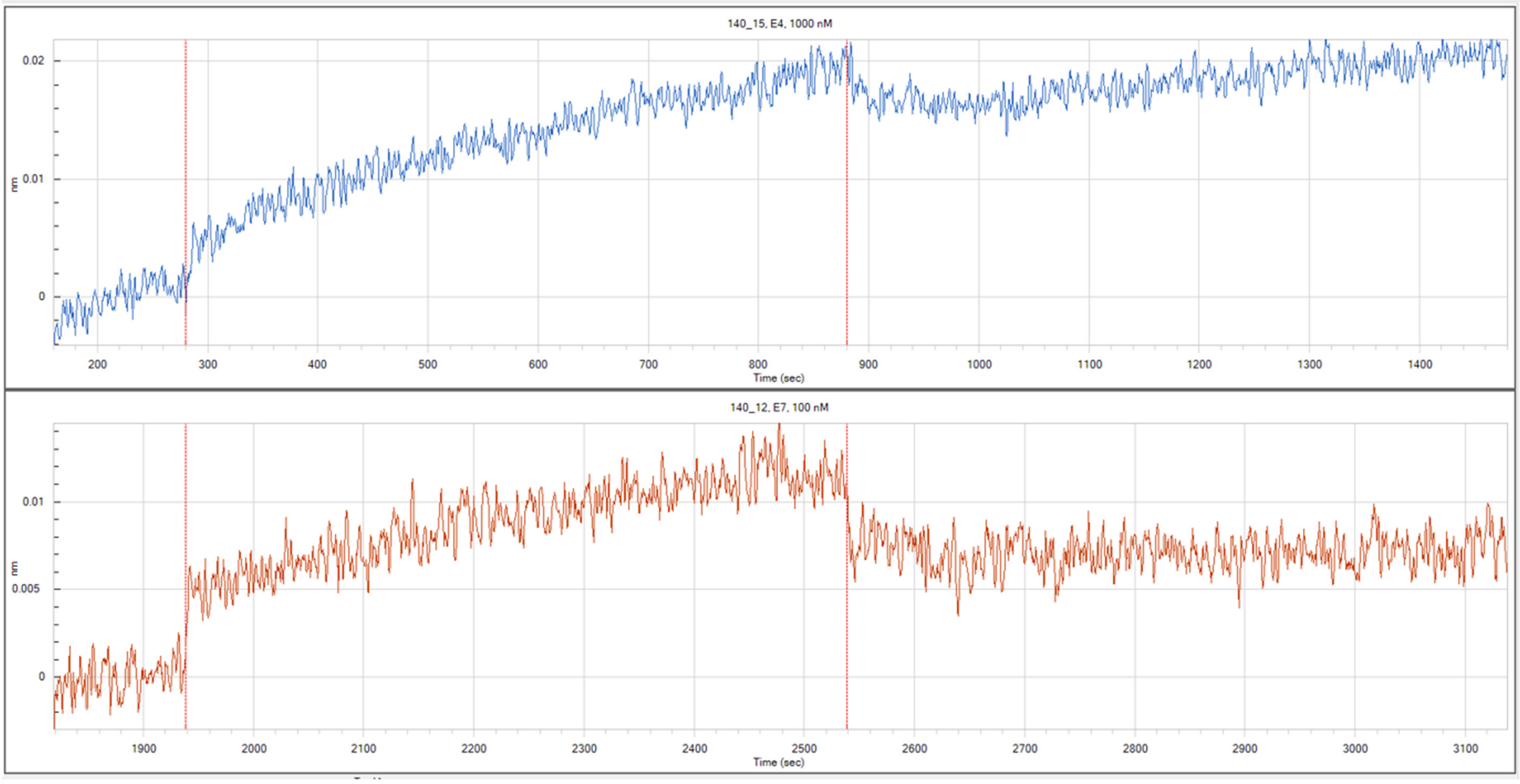
140_15, E4, 1000 nM
0.02
0.01
0
nm
200
300
400
500
600
700
800
900
1000
1100
1200
1300
1400
Time (sec)
140_12, E7, 100 nM
0.01
0.005
0
nm
1900
2000
2100
2200
2300
2400
2500
2600
2700
2800
2900
3000
3100
Time (sec)

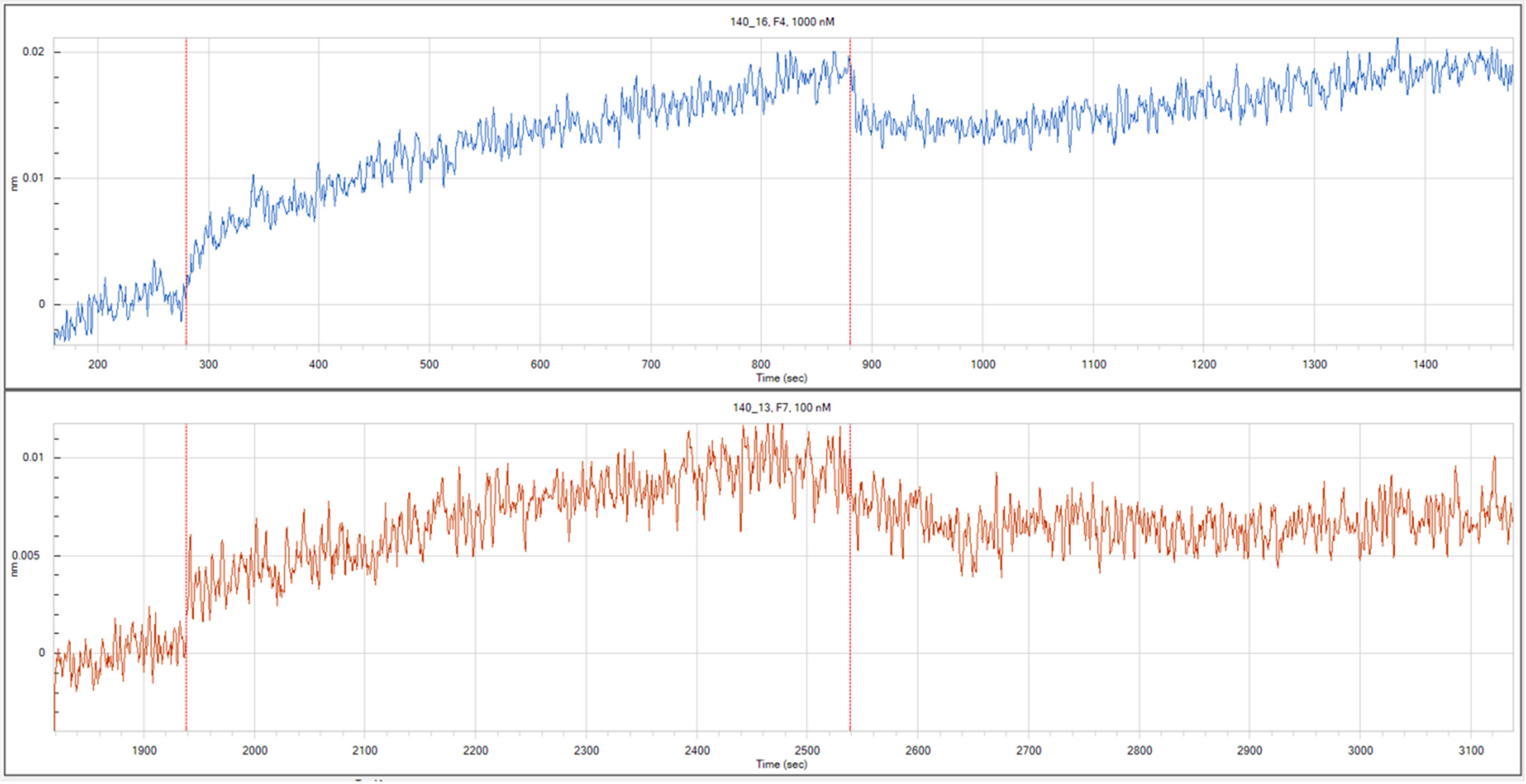
140_16, F4, 1000 nM
nm
0.02
0.01
0
200
300
400
500
600
700
800
900
1000
1100
1200
1300
1400
Time (sec)
140_13, F7, 100 nM
nm
0.01
0.005
0
1900
2000
2100
2200
2300
2400
2500
2600
2700
2800
2900
3000
3100
Time (sec)

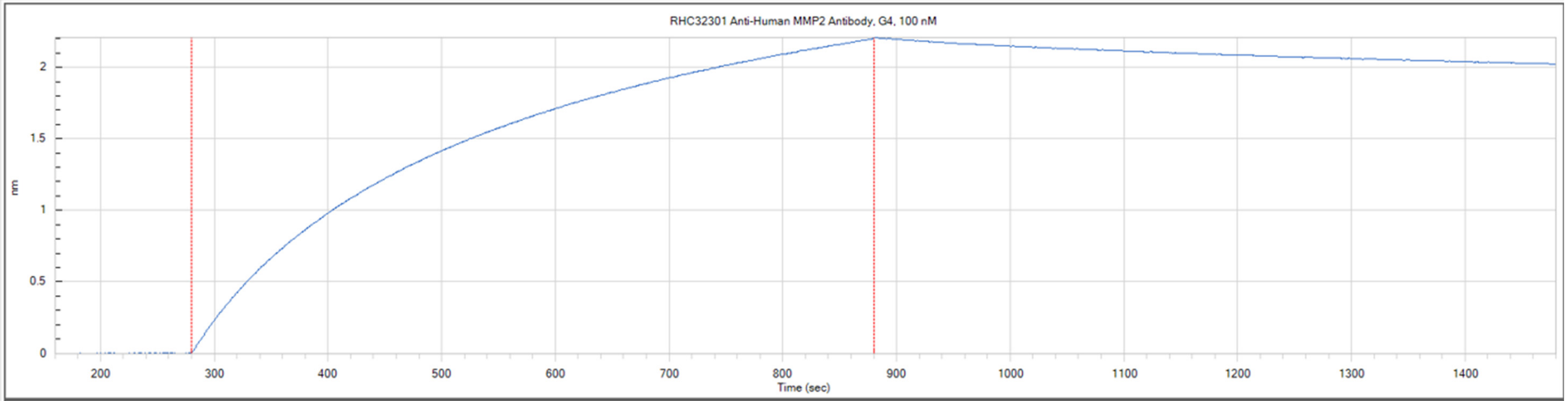
RHC32301 Anti-Human MMP2 Antibody, G4, 100 nM
nm
Time (sec)
0
0.5
1
1.5
2
200
300
400
500
600
700
800
900
1000
1100
1200
1300
1400

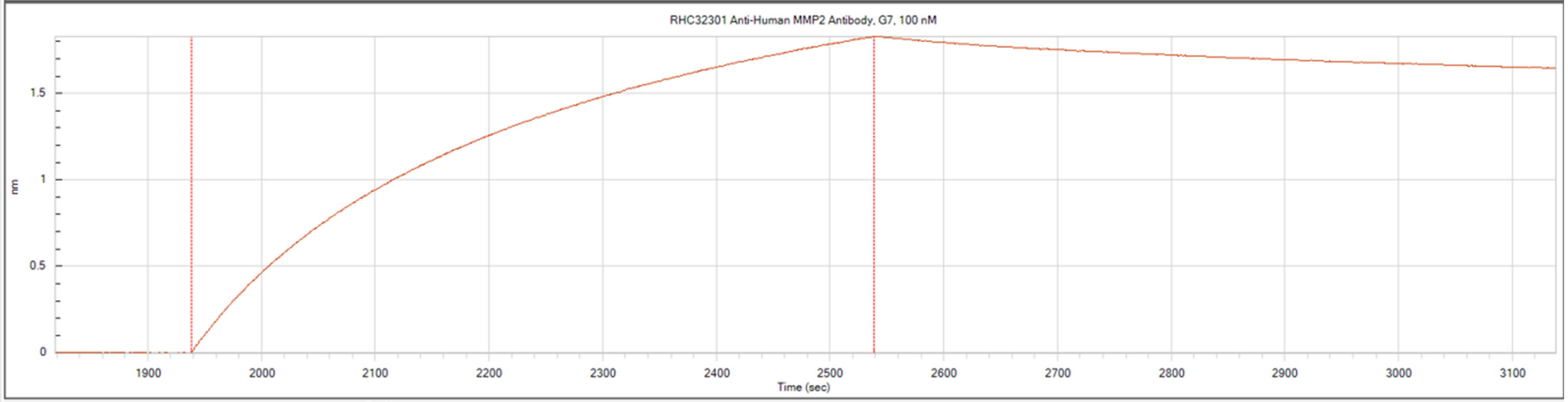
RHC32301 Anti-Human MMP2 Antibody, G7, 100 nM
nm
Time (sec)
0
0.5
1
1.5
1900
2000
2100
2200
2300
2400
2500
2600
2700
2800
2900
3000
3100

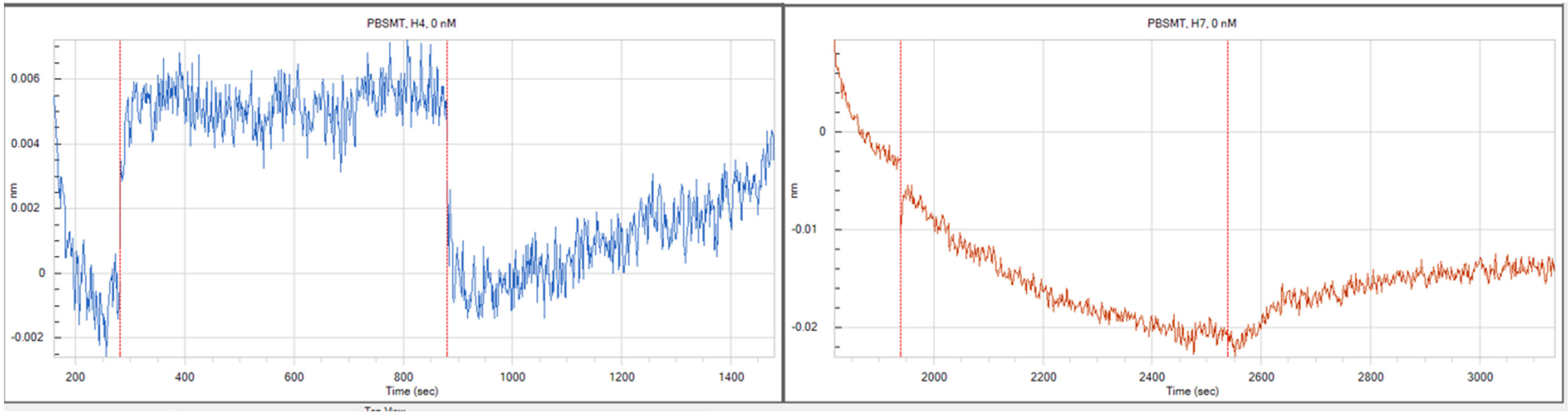

Figure S2: Sensorgrams obtained from analysis on NTA sensors, affinity determination of aptamers against MMP2

3. Analysis on NTA sensors, affinity determination of aptamers against PCSK9 (preparation #9, SBT).

The sensorgrams in Figure S3 are shown after subtraction of the reference-sensor signal and depict 3 stages of the experiment: baseline establishment, analyte association, and subsequent dissociation, separated by vertical dashed lines. The aptamer code or antibody name, well number, and tested analyte concentration are indicated. Sensorgrams are shown at different scales; the response in nm is indicated on the Y axis. For comparison, sensorgrams obtained for the control antibodies are shown, and, to illustrate the noise level in the study, a sensorgram for an analyte-free buffer solution is also shown (Xelari buf).

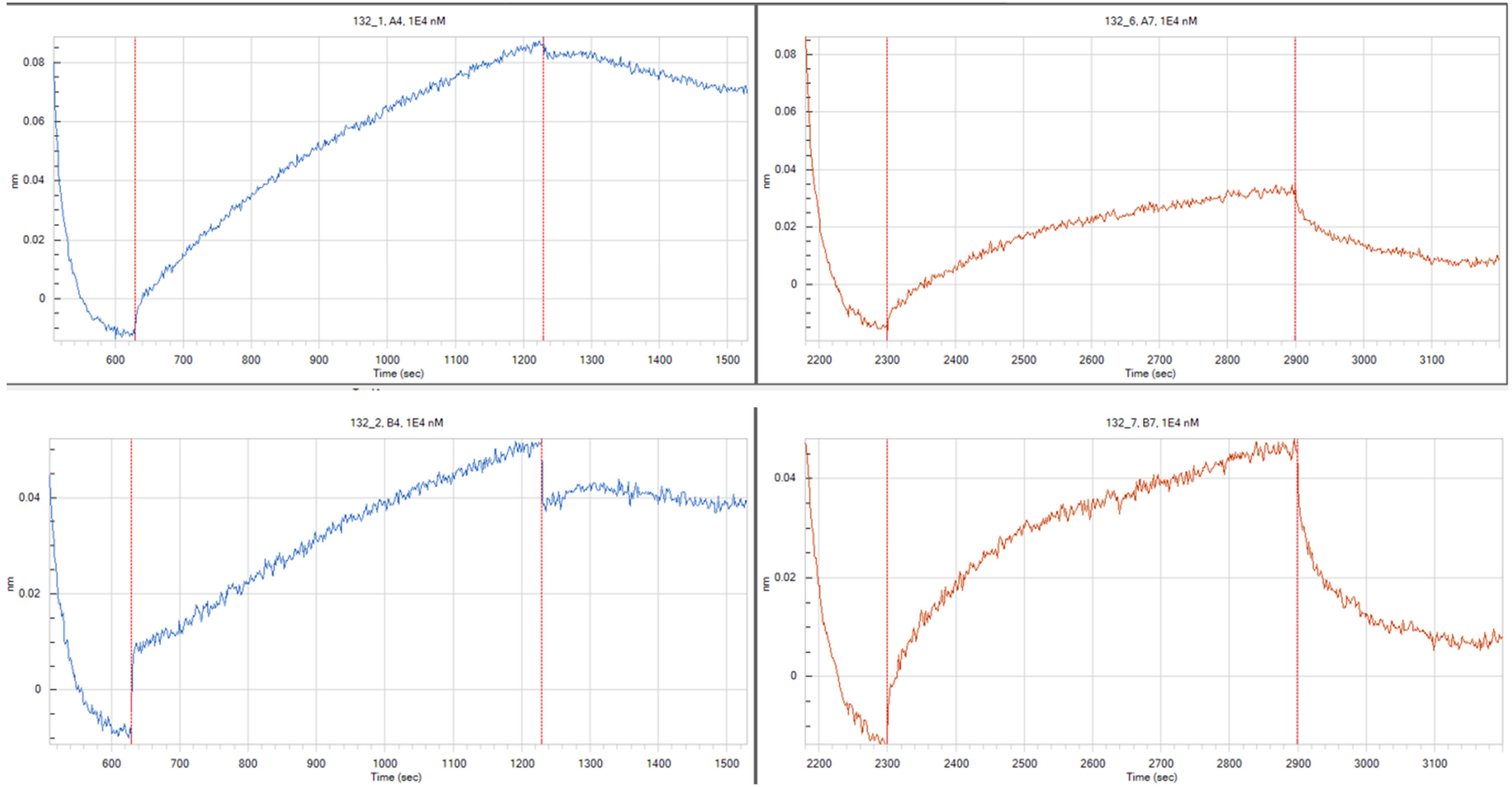

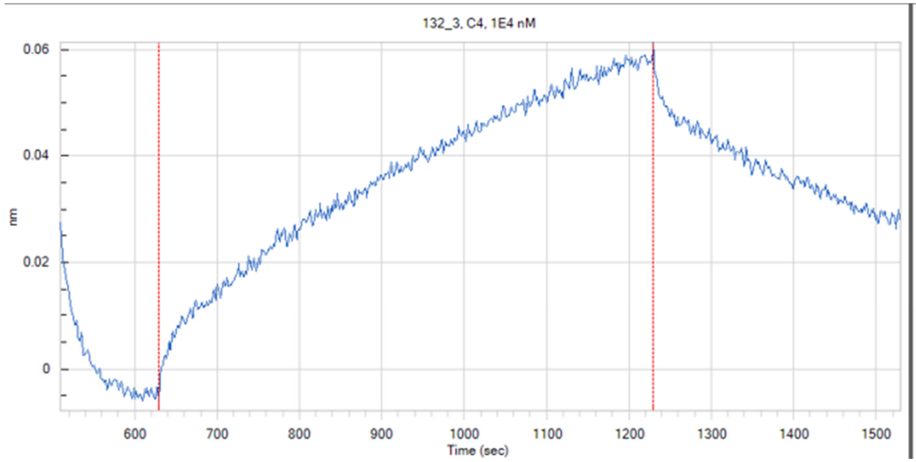
132_3, C4, 1E4 nM
0.06
0.04
0.02
0
nm
600
700
800
900
1000
1100
1200
1300
1400
1500
Time (sec)

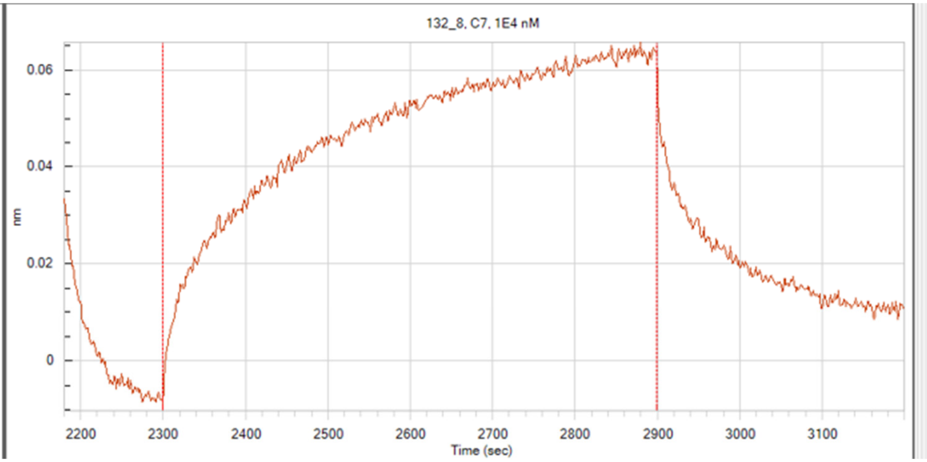
132_8, C7, 1E4 nM
0.06
0.04
0.02
0
nm
2200
2300
2400
2500
2600
2700
2800
2900
3000
3100
Time (sec)

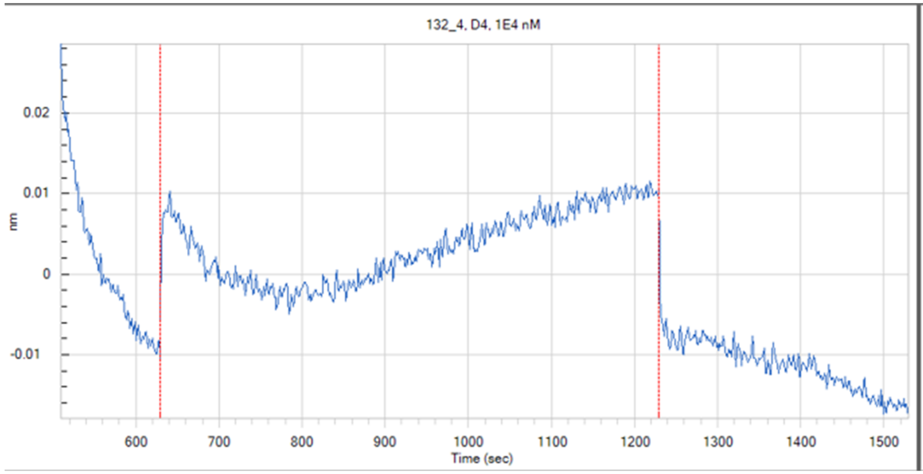
132_4, D4, 1E4 nM
0.02
0.01
0
-0.01
nm
600
700
800
900
1000
1100
1200
1300
1400
1500
Time (sec)

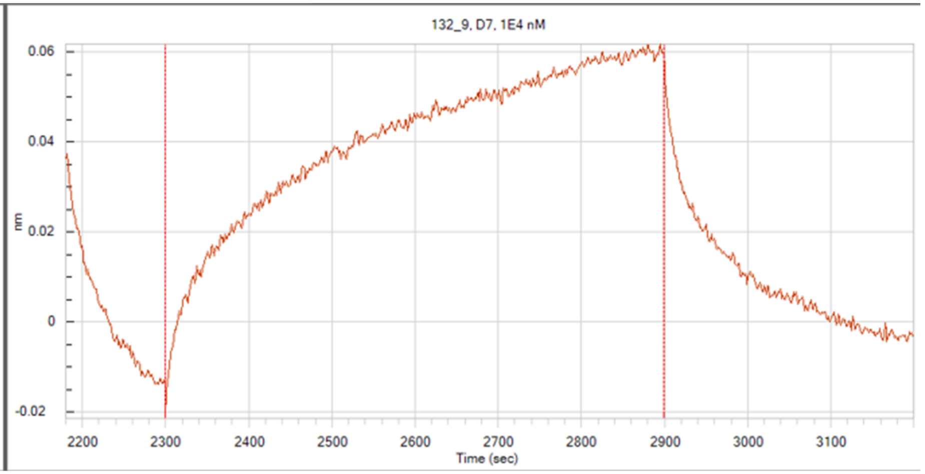
132_9, D7, 1E4 nM
0.06
0.04
0.02
0
-0.02
nm
2200
2300
2400
2500
2600
2700
2800
2900
3000
3100
Time (sec)

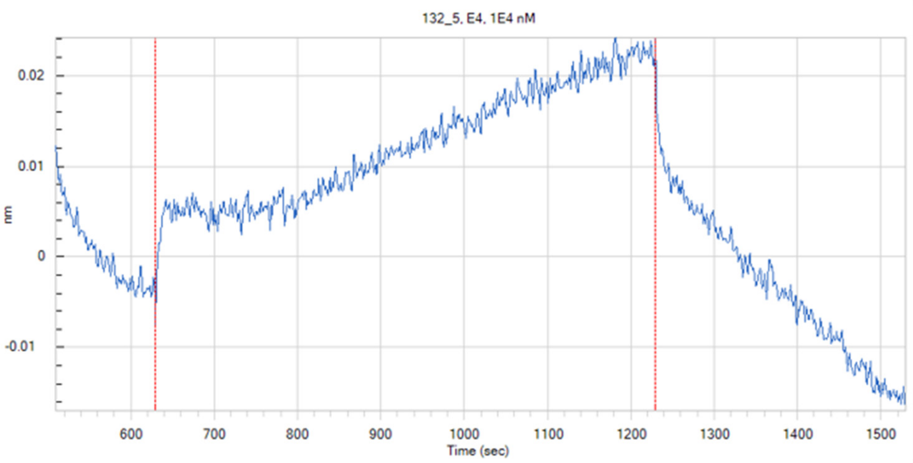
132_5, E4, 1E4 nM
0.02
0.01
0
-0.01
nm
600
700
800
900
1000
1100
1200
1300
1400
1500
Time (sec)

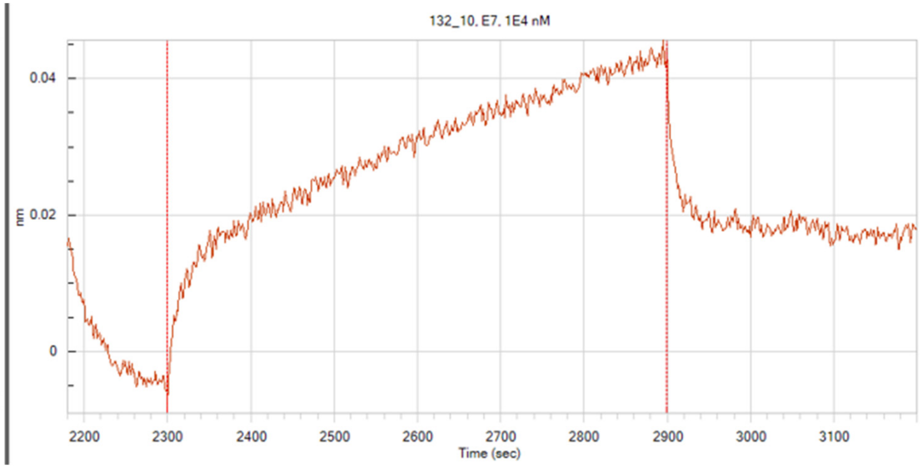
132_10, E7, 1E4 nM
0.04
0.02
0
nm
2200
2300
2400
2500
2600
2700
2800
2900
3000
3100
Time (sec)

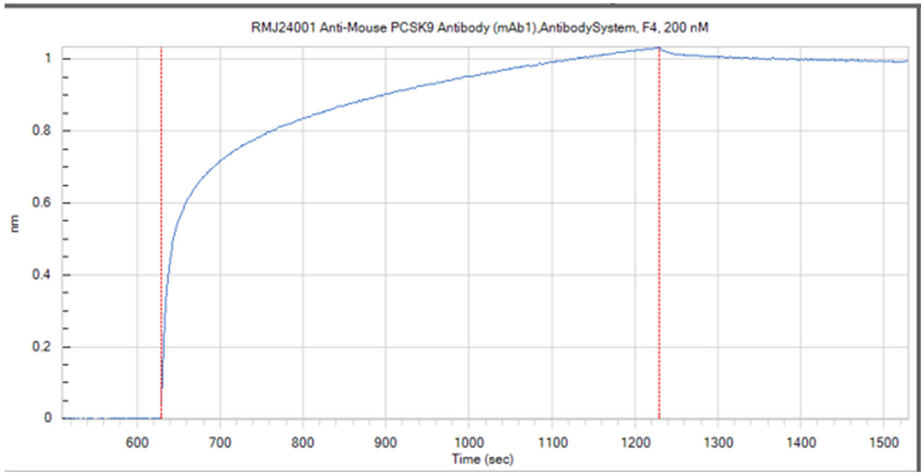
RMJ24001 Anti-Mouse PCSK9 Antibody (mAb1),AntibodySystem, F4, 200 nM
1
0.8
0.6
0.4
0.2
0
nm
600
700
800
900
1000
1100
1200
1300
1400
1500
Time (sec)

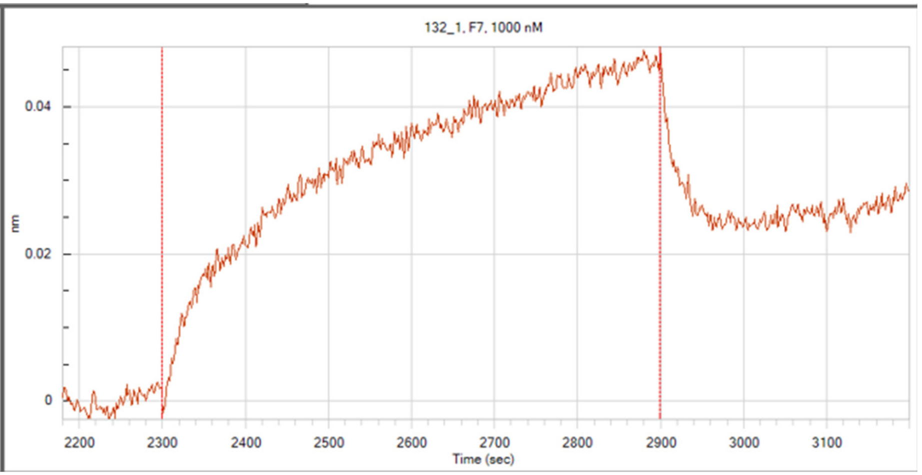
132_1, F7, 1000 nM
0.04
0.02
0
nm
2200
2300
2400
2500
2600
2700
2800
2900
3000
3100
Time (sec)

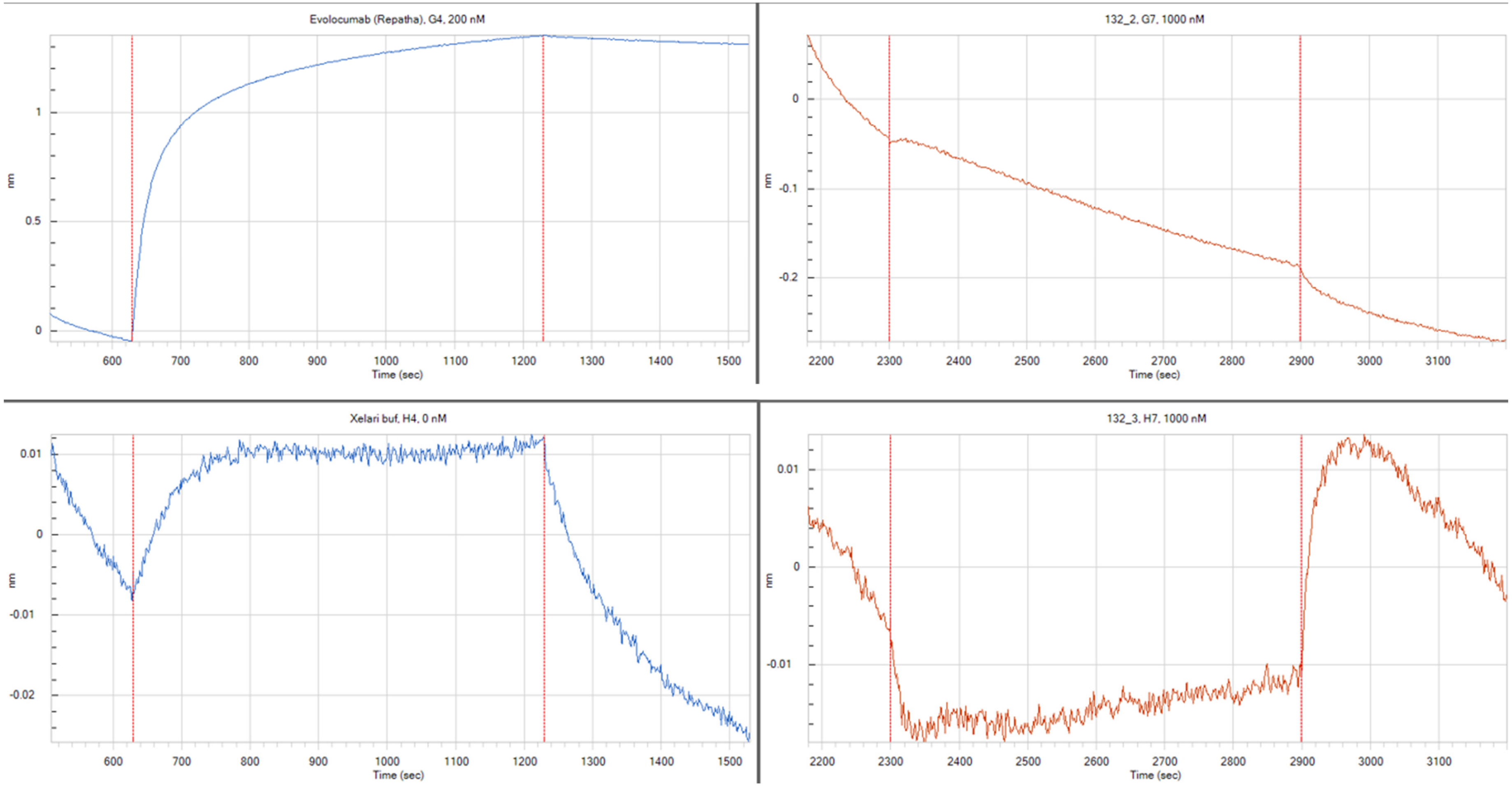

Figure S3: Sensorgrams obtained from analysis on NTA sensors, affinity determination of aptamers against PCSK9 (preparation #9, SBT)

4. Analysis on NTA sensors, affinity determination of aptamers against PCSK9 (SinoBiological).

The sensorgrams in Figure S4 are shown after subtraction of the reference-sensor signal and depict 3 stages of the experiment: baseline establishment, analyte association, and subsequent dissociation, separated by vertical dashed lines. The aptamer code or antibody name, well number, and tested analyte concentration are indicated. Sensorgrams are shown at different scales; the response in nm is indicated on the Y axis.

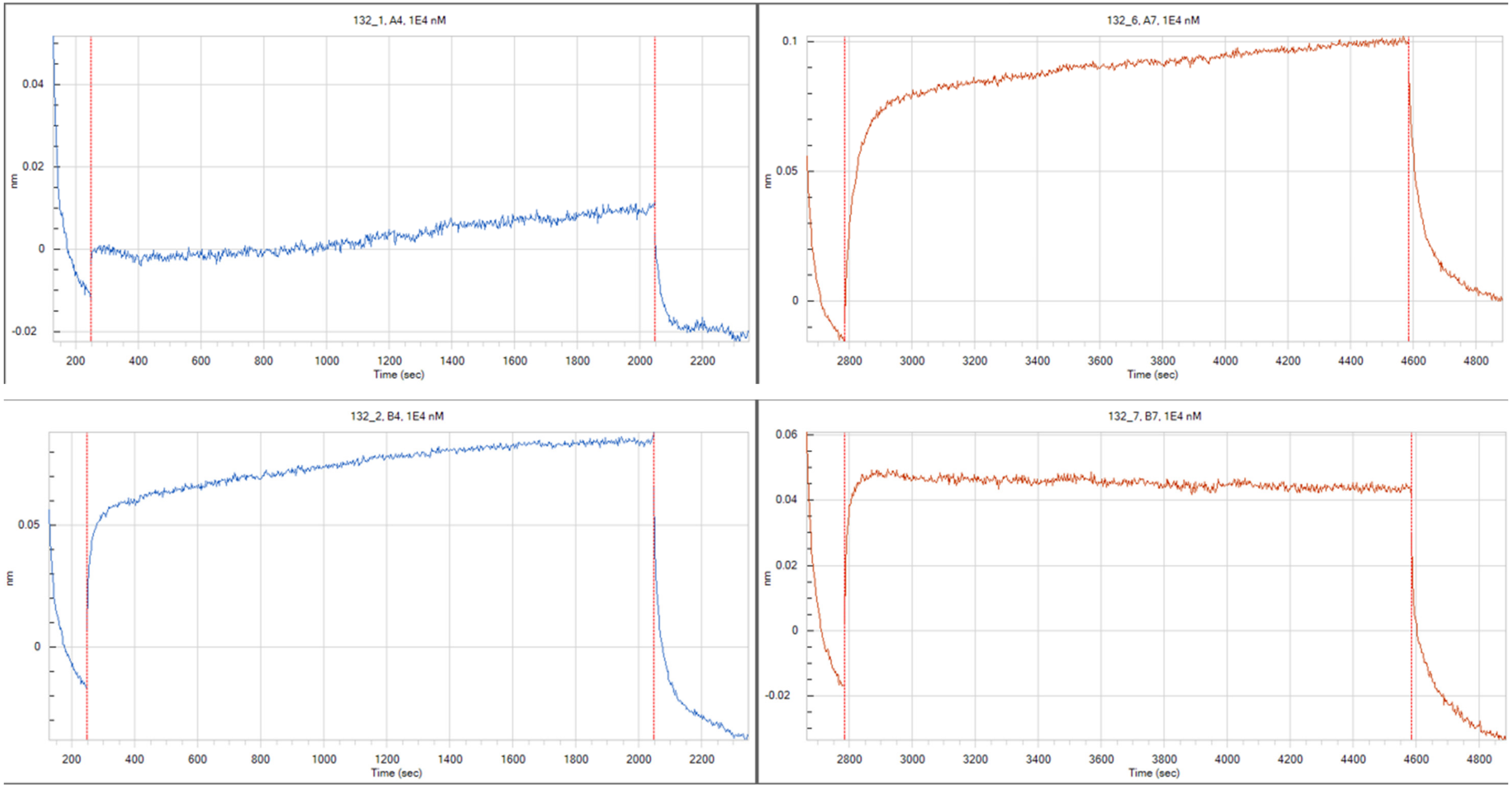

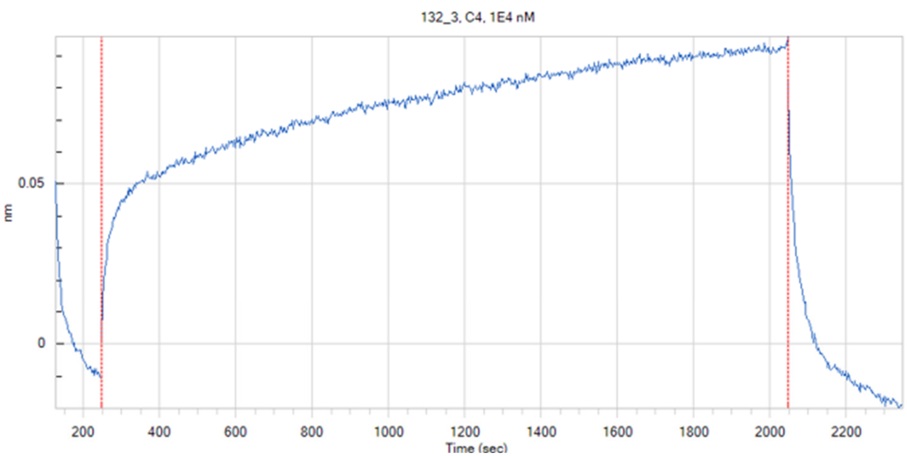
132_3, C4, 1E4 nM
nm
0.05
0
200
400
600
800
1000
1200
1400
1600
1800
2000
2200
Time (sec)

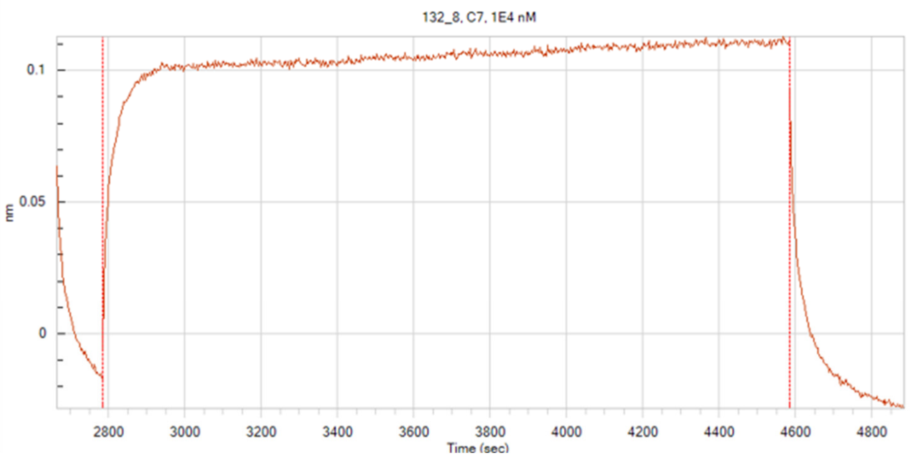
132_8, C7, 1E4 nM
nm
0.1
0.05
0
2800
3000
3200
3400
3600
3800
4000
4200
4400
4600
4800
Time (sec)

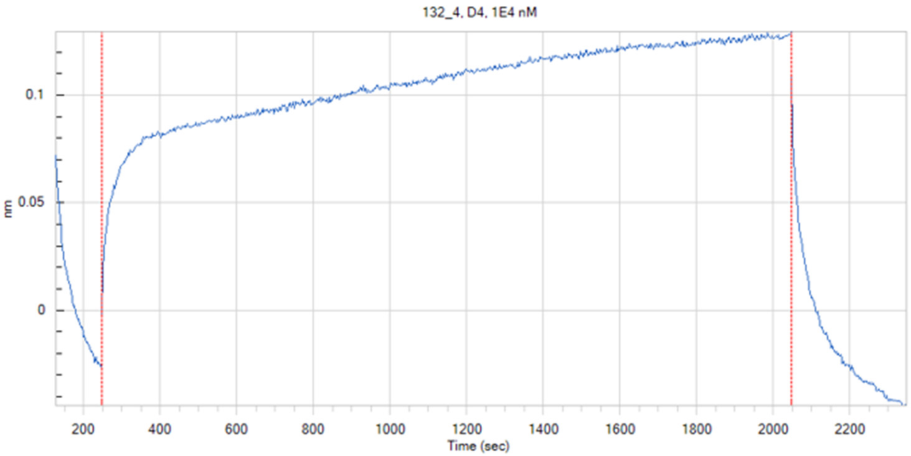
132_4, D4, 1E4 nM
nm
0.1
0.05
0
200
400
600
800
1000
1200
1400
1600
1800
2000
2200
Time (sec)

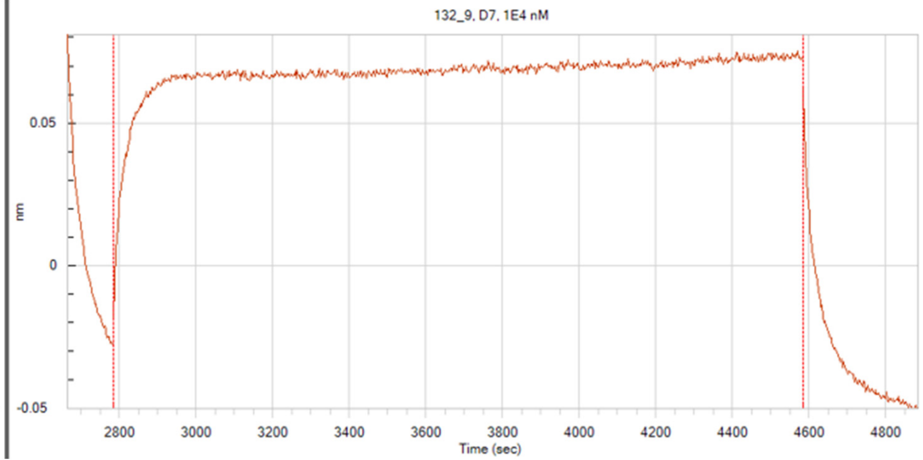
132_9, D7, 1E4 nM
nm
0.05
0
-0.05
2800
3000
3200
3400
3600
3800
4000
4200
4400
4600
4800
Time (sec)

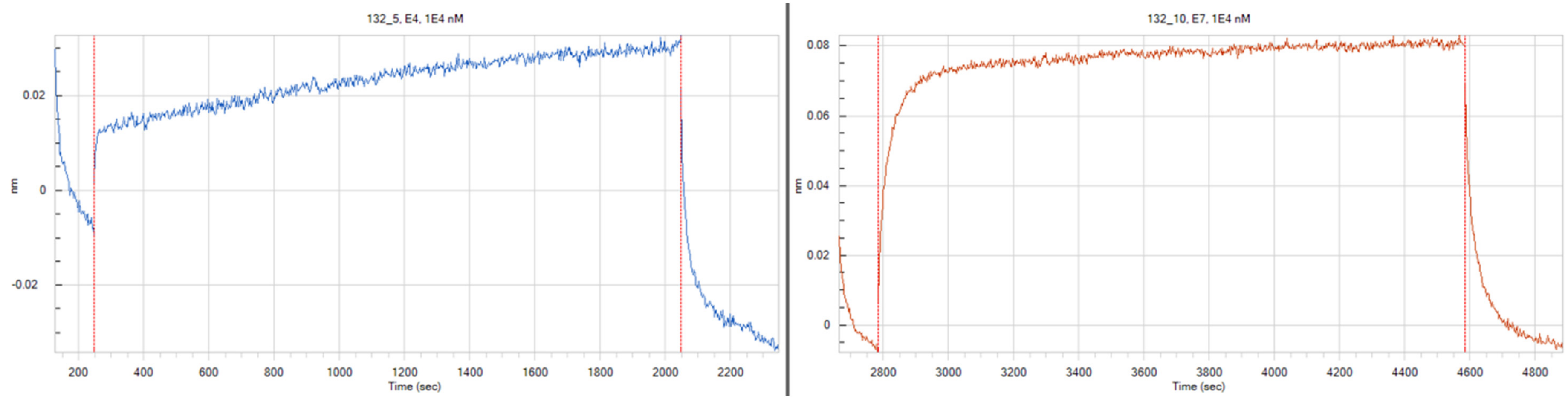

Figure S4: Sensorgrams obtained from analysis on NTA sensors, affinity determination of aptamers against PCSK9 (SinoBiological)

5. Analysis on NTA sensors, affinity determination of aptamers against MMP9.

The sensorgrams in Figure S5 are shown after subtraction of the reference-sensor signal and depict 3 stages of the experiment: baseline establishment, analyte association, and subsequent dissociation, separated by vertical dashed lines. The aptamer code or antibody name, well number, and tested analyte concentration are indicated. Sensorgrams are shown at different scales; the response in nm is indicated on the Y axis. For comparison, sensorgrams obtained for the control antibody are shown.

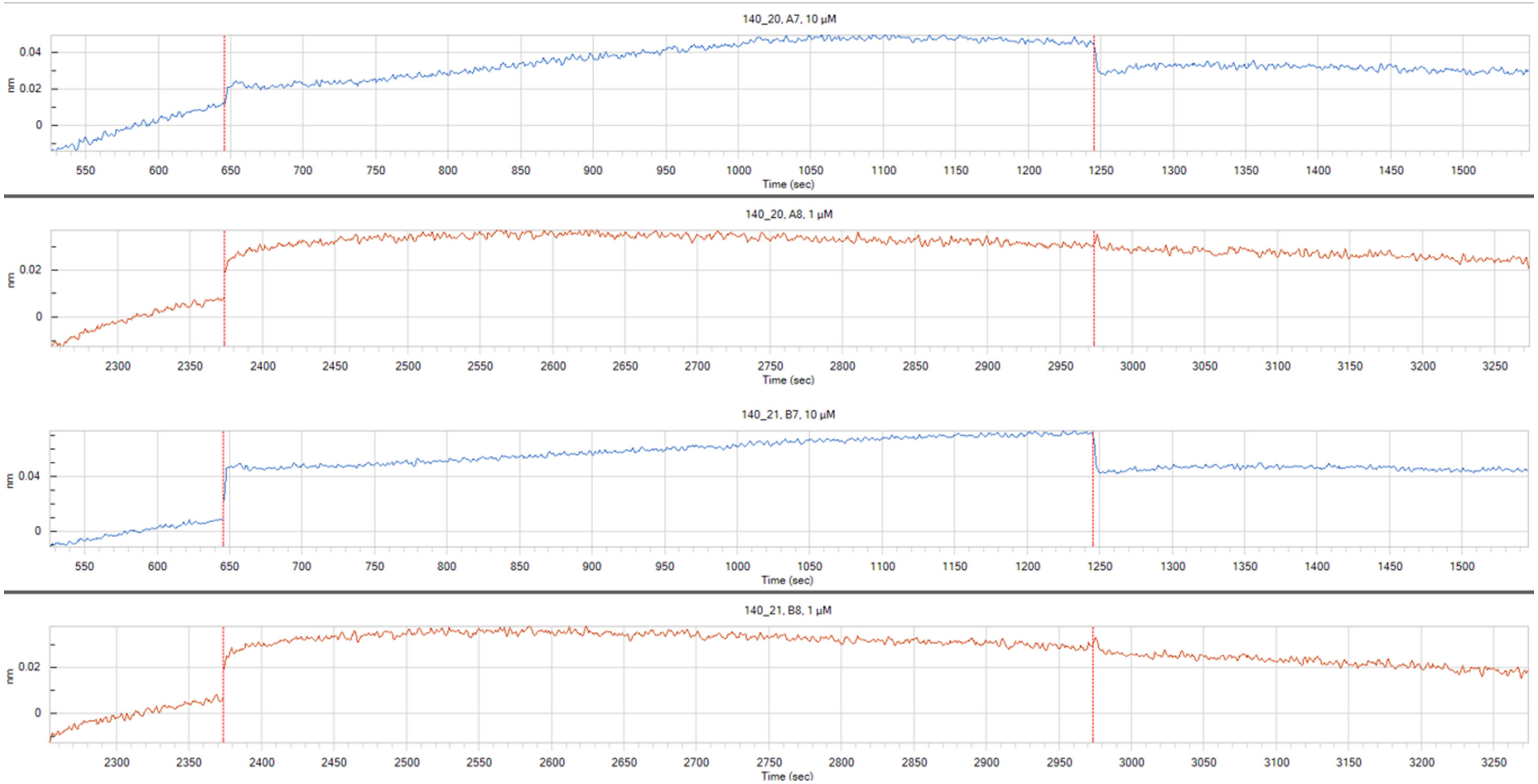

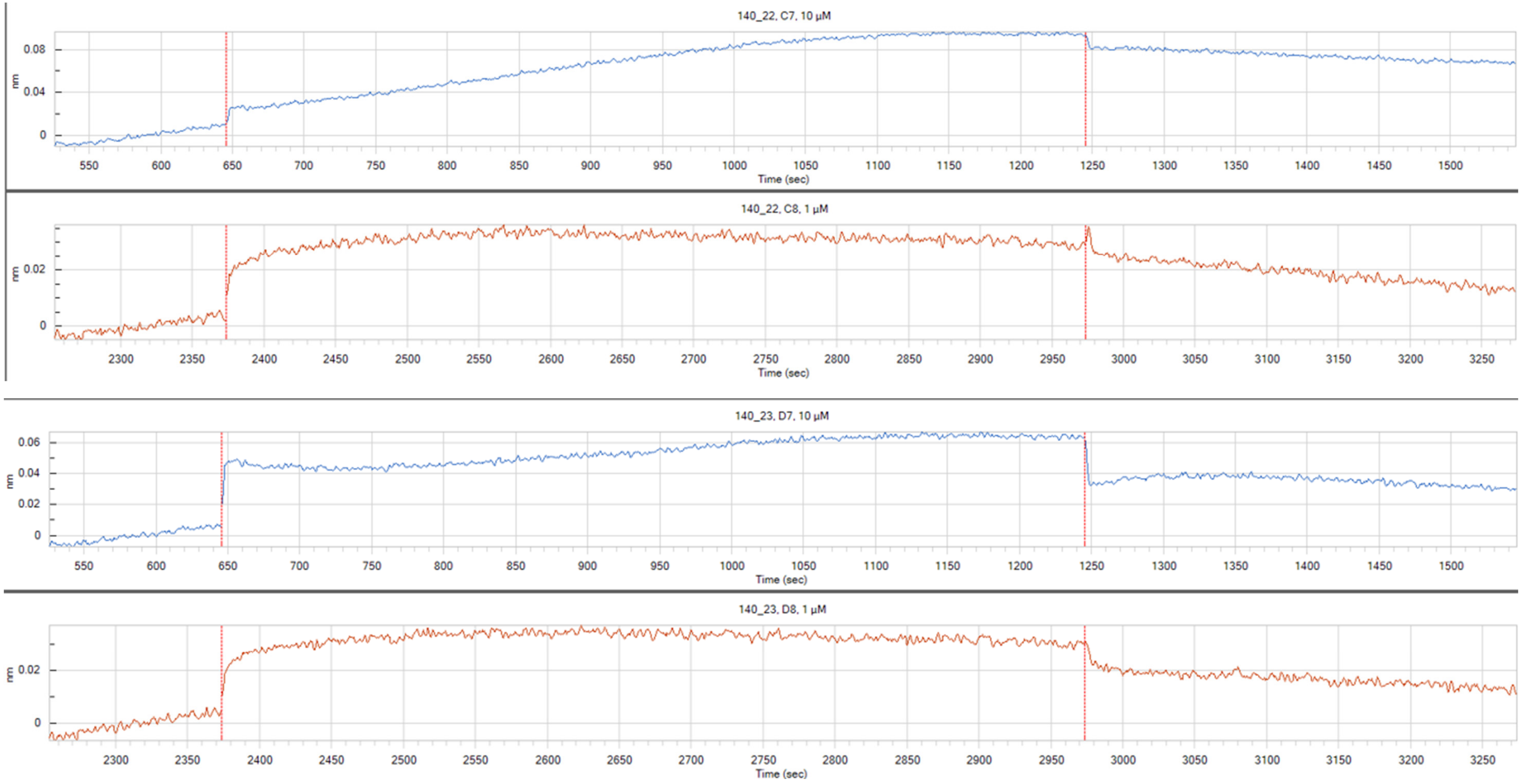
140_22, C7, 10 µM
nm
0.08
0.04
0
550 600 650 700 750 800 850 900 950 1000 1050 1100 1150 1200 1250 1300 1350 1400 1450 1500
Time (sec)
140_22, C8, 1 µM
nm
0.02
0
2300 2350 2400 2450 2500 2550 2600 2650 2700 2750 2800 2850 2900 2950 3000 3050 3100 3150 3200 3250
Time (sec)
140_23, D7, 10 µM
nm
0.06
0.04
0.02
0
550 600 650 700 750 800 850 900 950 1000 1050 1100 1150 1200 1250 1300 1350 1400 1450 1500
Time (sec)
140_23, D8, 1 µM
nm
0.02
0
2300 2350 2400 2450 2500 2550 2600 2650 2700 2750 2800 2850 2900 2950 3000 3050 3100 3150 3200 3250
Time (sec)

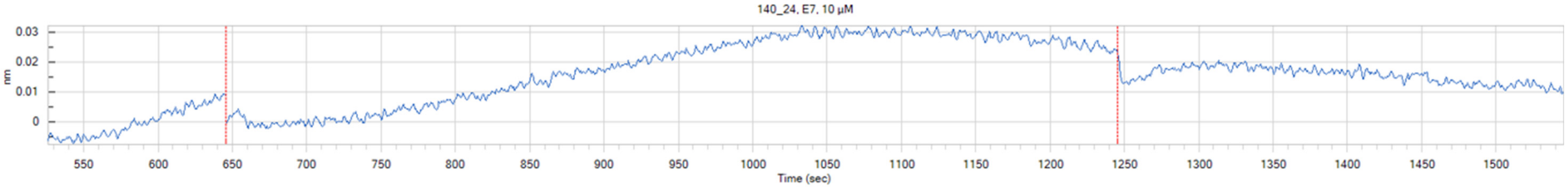
140_24, E7, 10 µM
nm
0.03
0.02
0.01
0
550 600 650 700 750 800 850 900 950 1000 1050 1100 1150 1200 1250 1300 1350 1400 1450 1500
Time (sec)

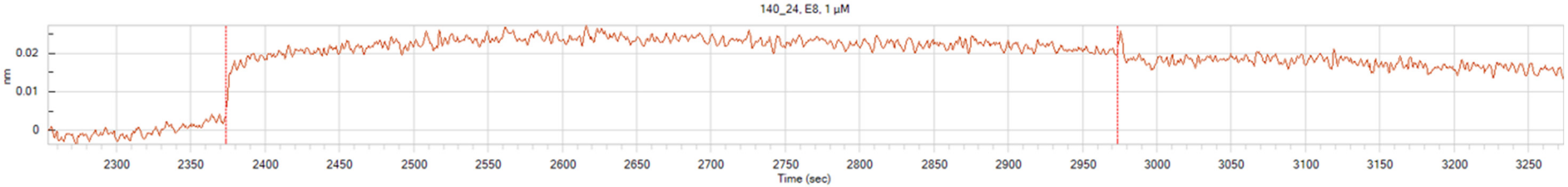
140_24, E8, 1 µM
nm
0.02
0.01
0
2300 2350 2400 2450 2500 2550 2600 2650 2700 2750 2800 2850 2900 2950 3000 3050 3100 3150 3200 3250
Time (sec)

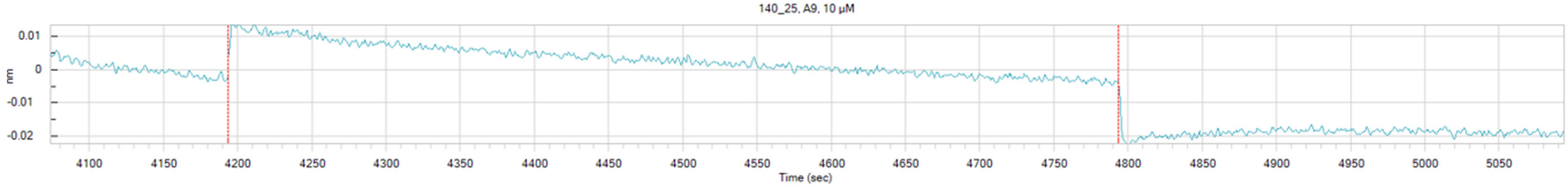
140_25, A9, 10 µM
nm
0.01
0
-0.01
-0.02
4100 4150 4200 4250 4300 4350 4400 4450 4500 4550 4600 4650 4700 4750 4800 4850 4900 4950 5000 5050
Time (sec)

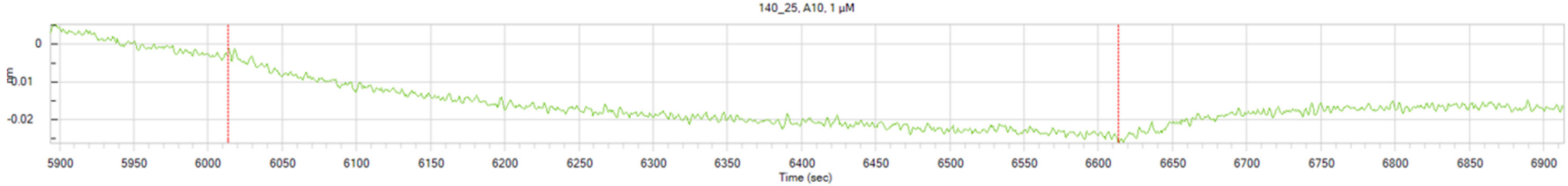
140_25, A10, 1 µM
nm
0
-0.01
-0.02
5900 5950 6000 6050 6100 6150 6200 6250 6300 6350 6400 6450 6500 6550 6600 6650 6700 6750 6800 6850 6900
Time (sec)

140_26, B9, 10 µM

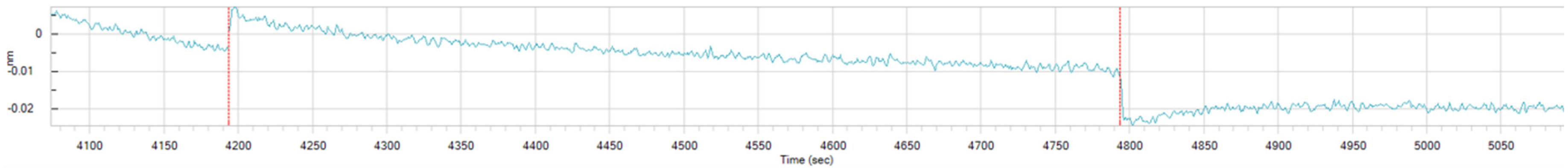

140_26, B10, 1 µM

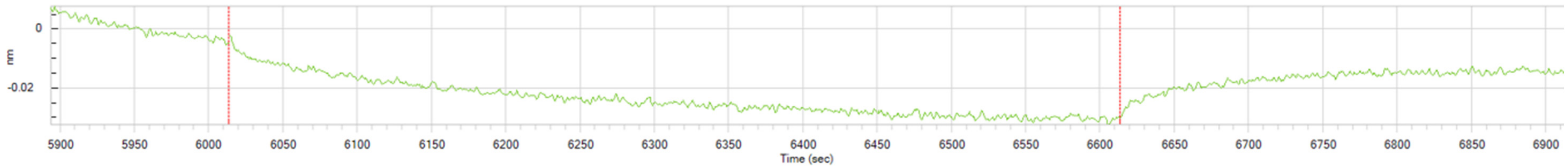

140_27, C9, 10 µM

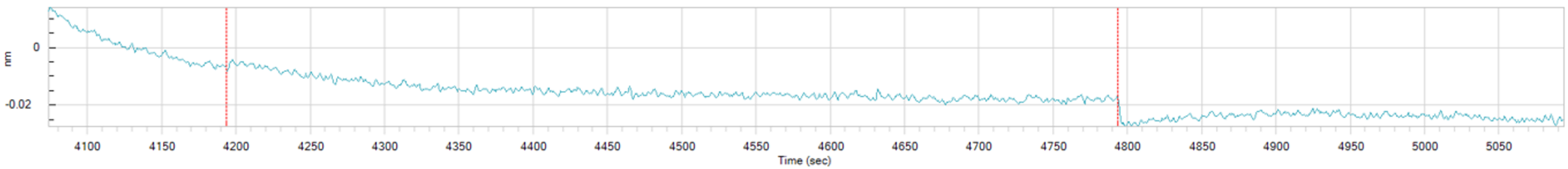

140_27, C10, 1 µM

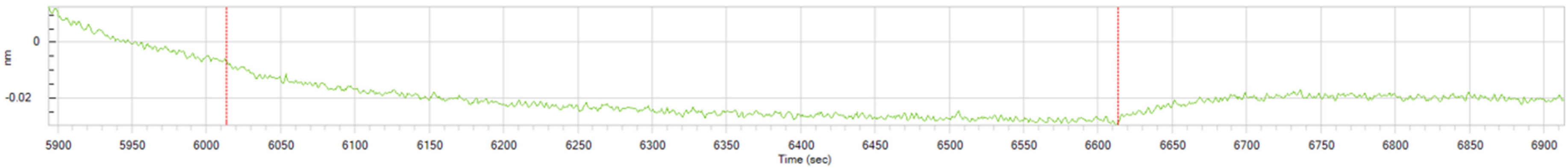

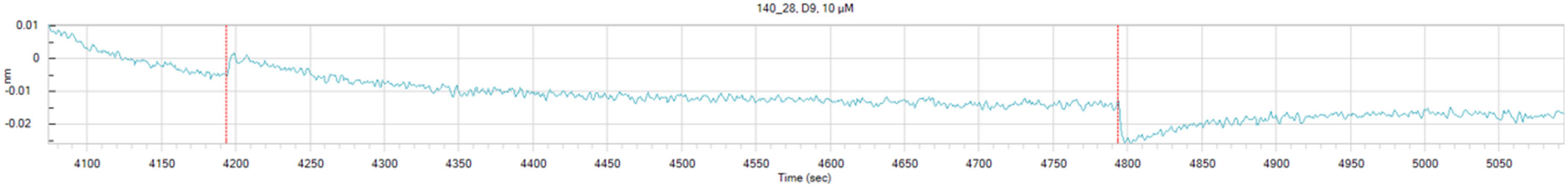
140_28, D9, 10 µM
nm
0.01
0
-0.01
-0.02
4100 4150 4200 4250 4300 4350 4400 4450 4500 4550 4600 4650 4700 4750 4800 4850 4900 4950 5000 5050
Time (sec)

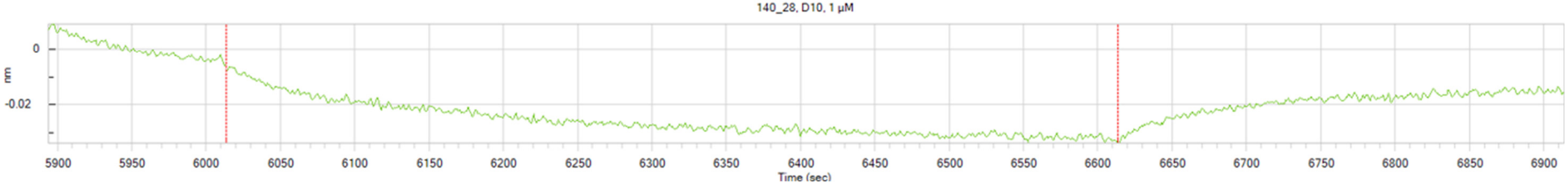
140_28, D10, 1 µM
nm
0
-0.02
5900 5950 6000 6050 6100 6150 6200 6250 6300 6350 6400 6450 6500 6550 6600 6650 6700 6750 6800 6850 6900
Time (sec)

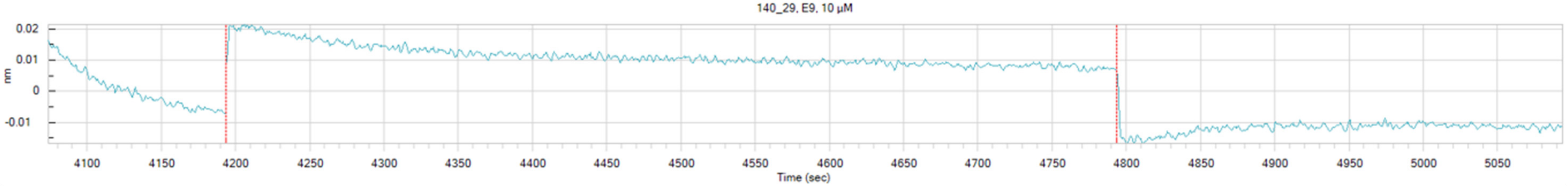
140_29, E9, 10 µM
nm
0.02
0.01
0
-0.01
4100 4150 4200 4250 4300 4350 4400 4450 4500 4550 4600 4650 4700 4750 4800 4850 4900 4950 5000 5050
Time (sec)

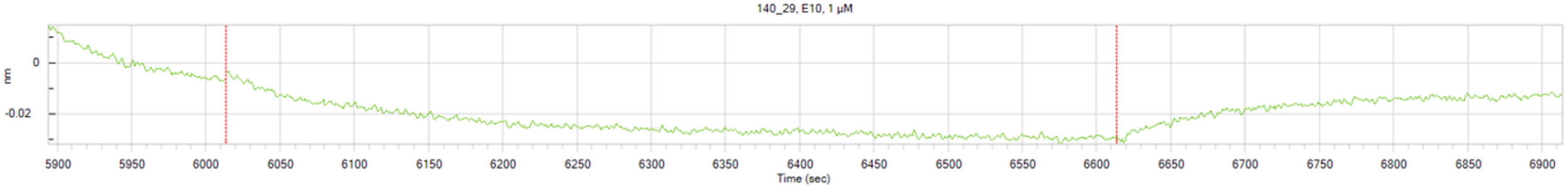
140_29, E10, 1 µM
nm
0
-0.02
5900 5950 6000 6050 6100 6150 6200 6250 6300 6350 6400 6450 6500 6550 6600 6650 6700 6750 6800 6850 6900
Time (sec)

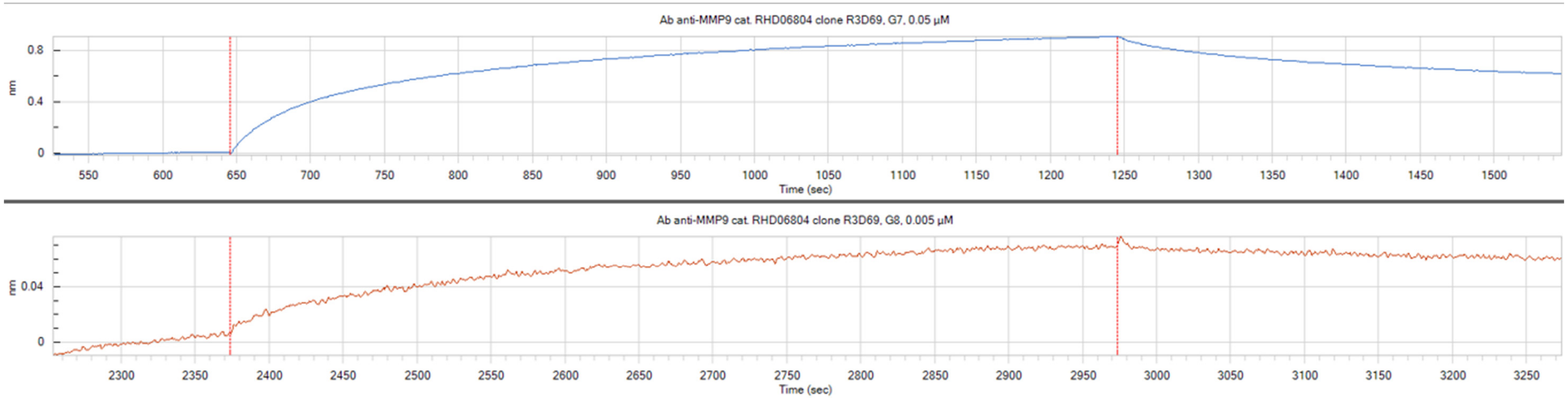

Figure S5: Sensorgrams obtained from analysis on NTA sensors, affinity determination of aptamers against MMP9

6. Analysis on NTA sensors, affinity determination of aptamers against MMP10.

The sensorgrams in Figure S6 are shown after subtraction of the reference-sensor signal and depict 3 stages of the experiment: baseline establishment, analyte association, and subsequent dissociation, separated by vertical dashed lines. The aptamer code or antibody name, well number, and tested analyte concentration are indicated. Sensorgrams are shown at different scales; the response in nm is indicated on the Y axis. For comparison, sensorgrams are shown for the purchased antibody that had been intended for use as a control, given the absence of its detectable affinity to the target protein.

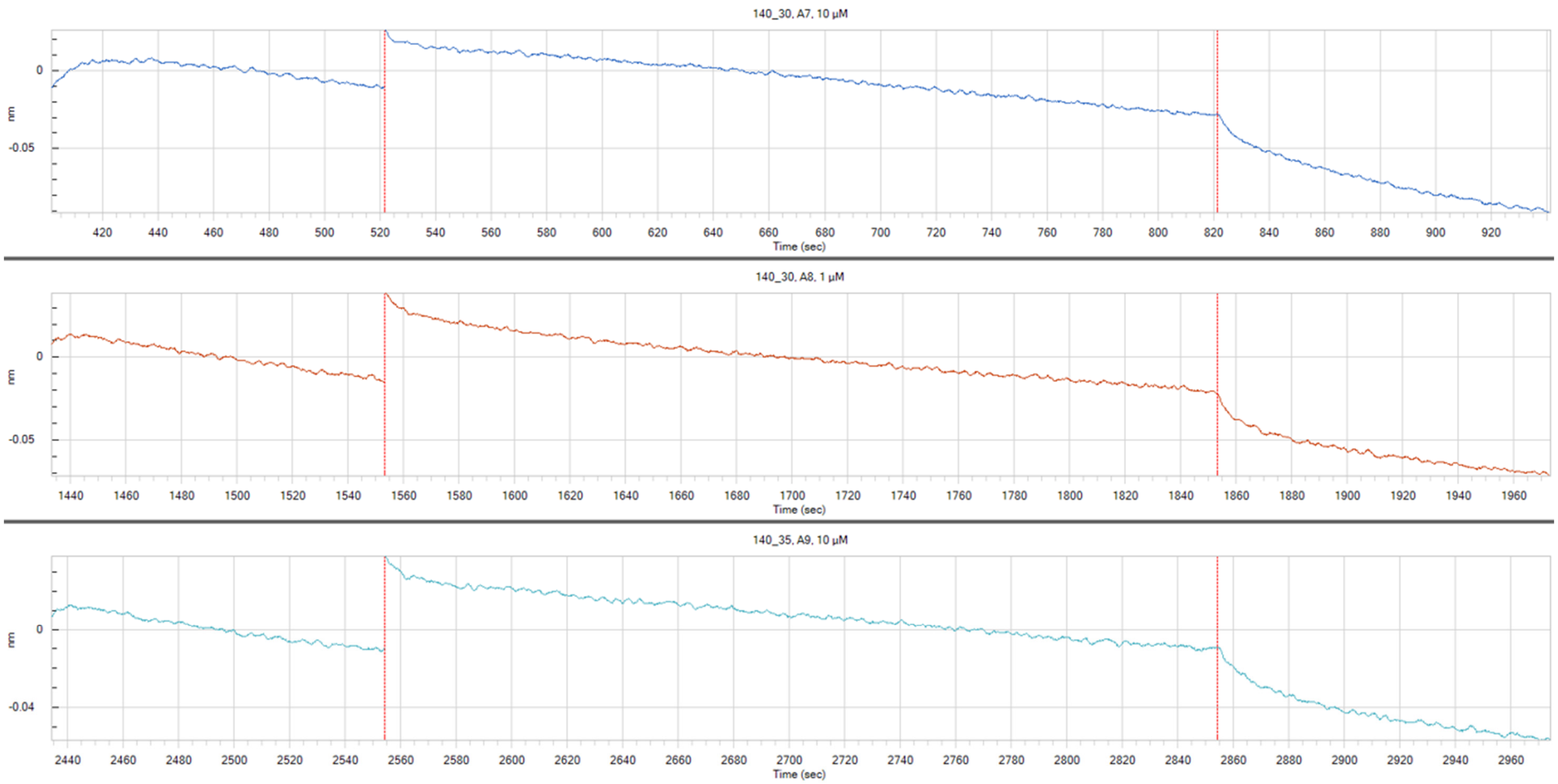

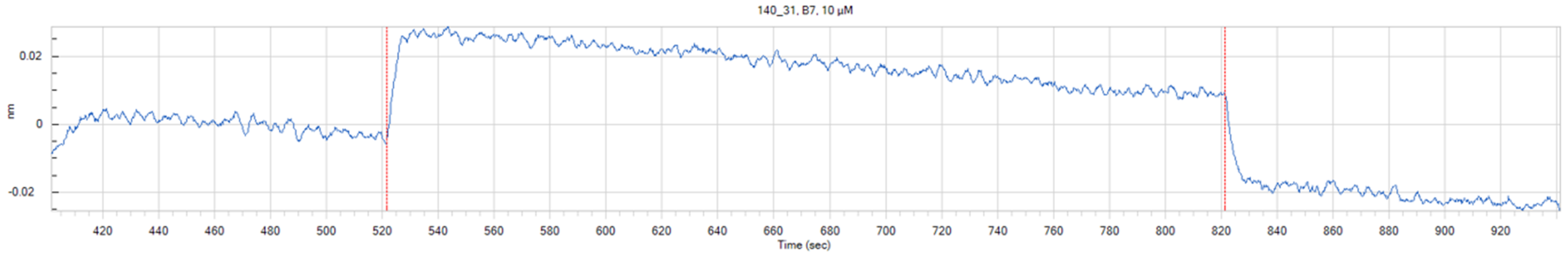
140_31, B7, 10 µM
nm
Time (sec)

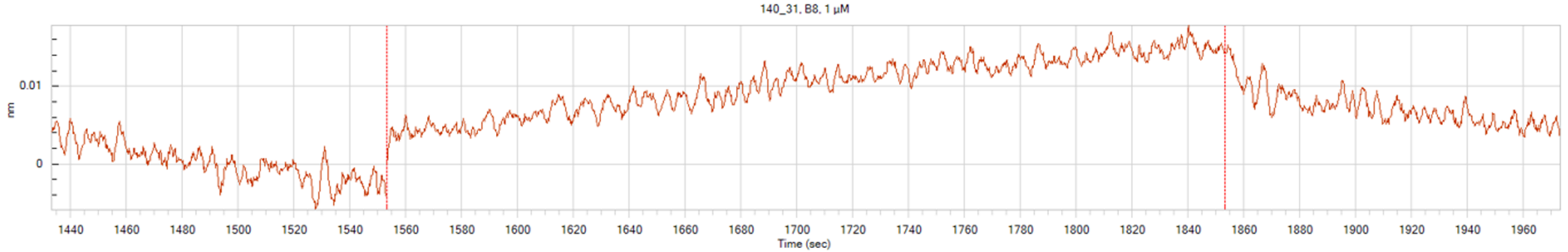
140_31, B8, 1 µM
nm
Time (sec)

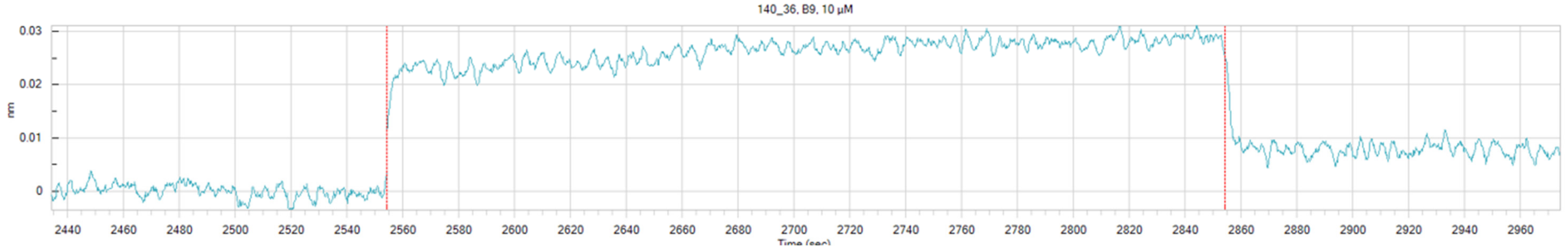
140_36, B9, 10 µM
nm

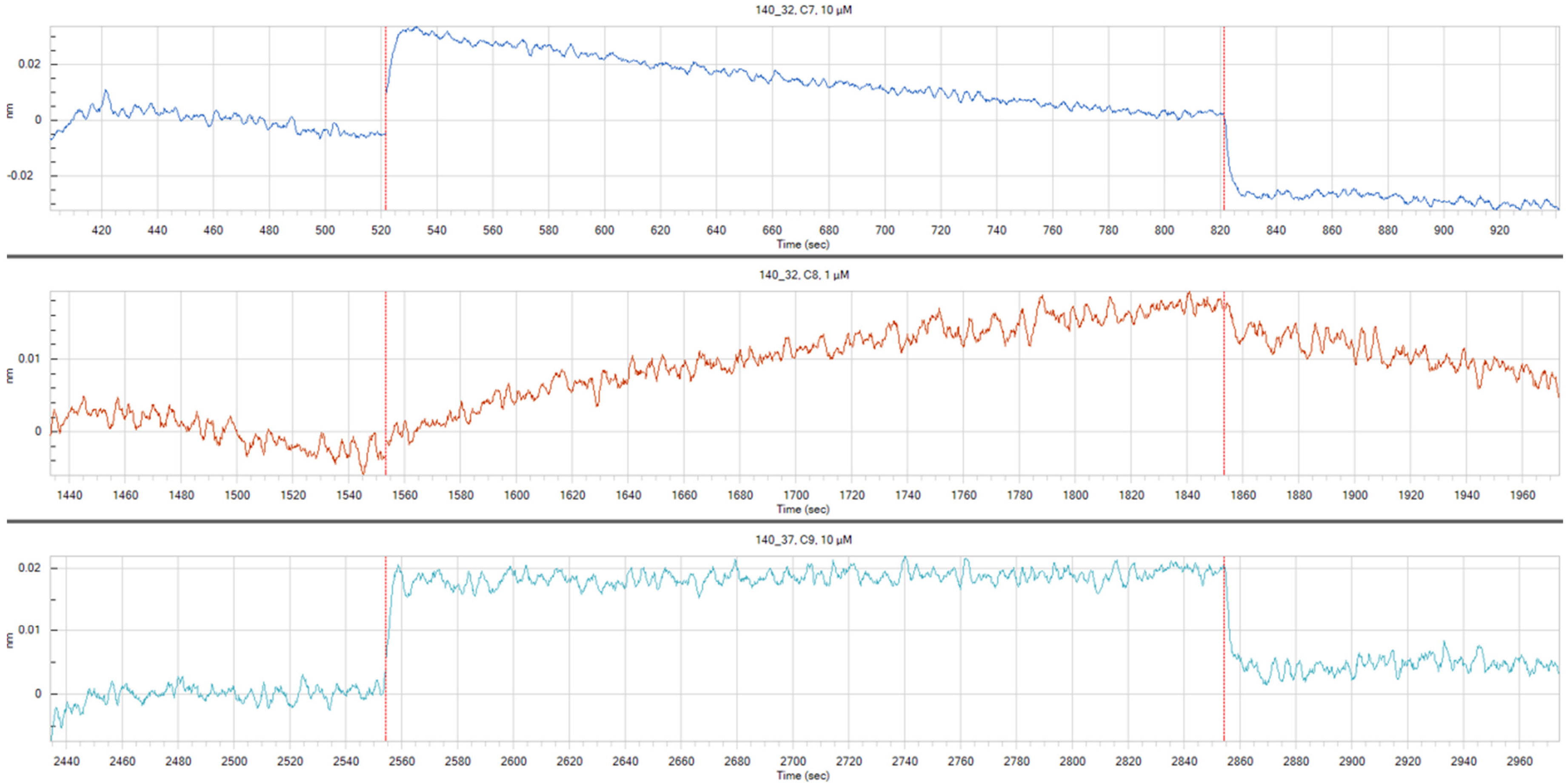
140_32, C7, 10 µM
nm
Time (sec)
140_32, C8, 1 µM
nm
Time (sec)
140_37, C9, 10 µM
nm
Time (sec)

140_33, D7, 10 µM

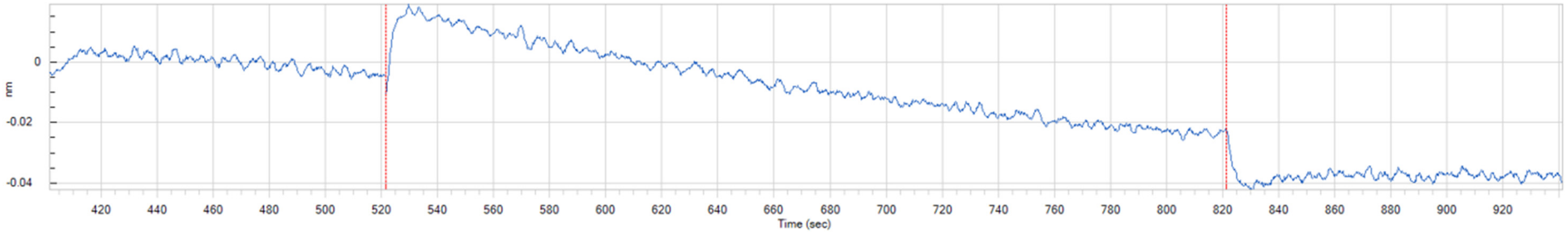

140_33, D8, 1 µM

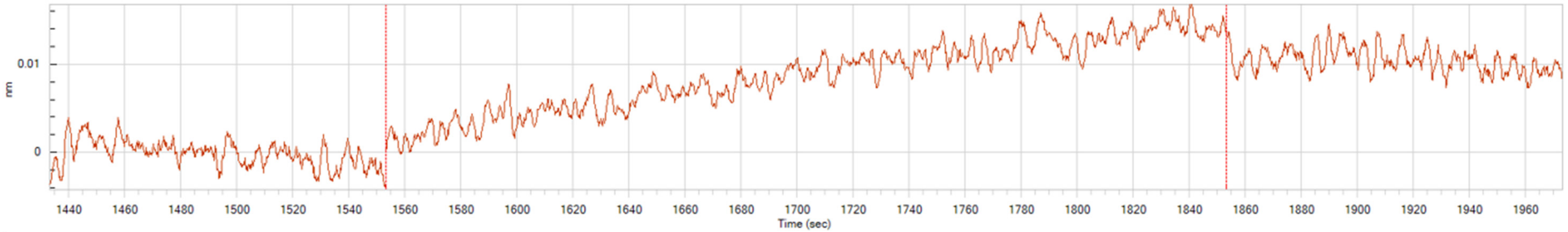

140_38, D9, 10 µM

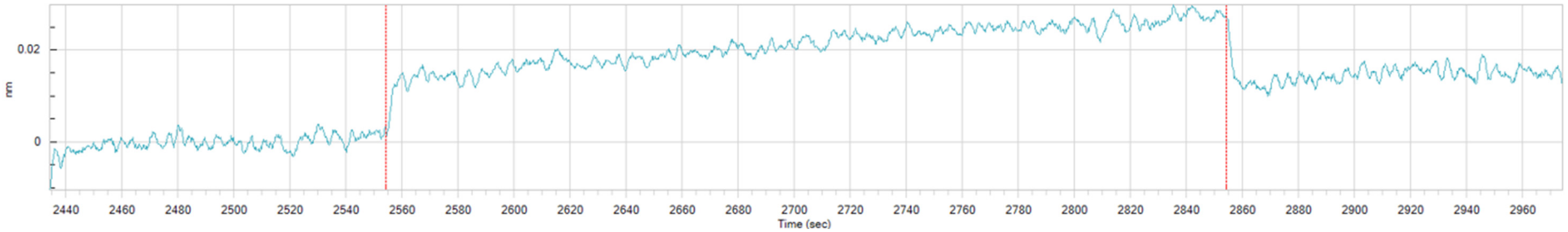

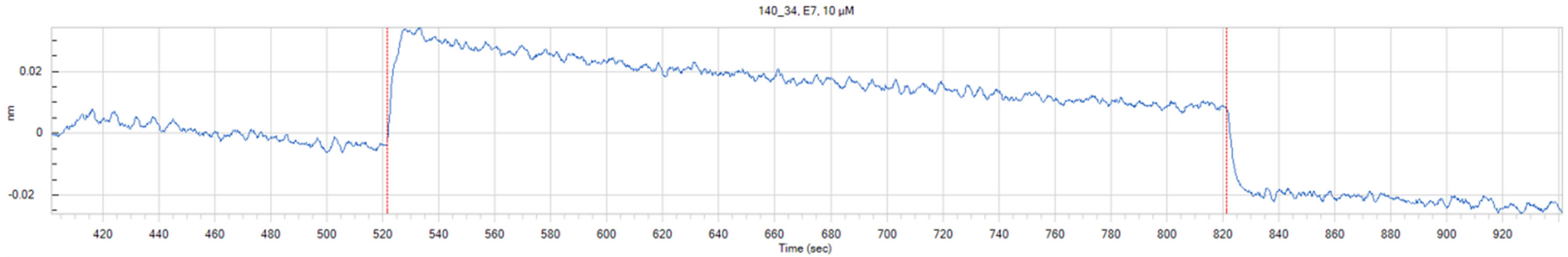
140_34, E7, 10 µM
nm
0.02
0
-0.02
420 440 460 480 500 520 540 560 580 600 620 640 660 680 700 720 740 760 780 800 820 840 860 880 900 920
Time (sec)

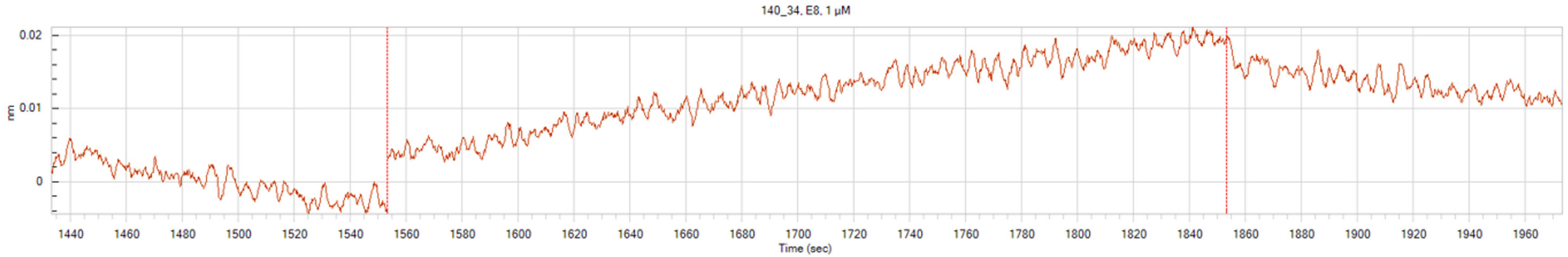
140_34, E8, 1 µM
nm
0.02
0.01
0
1440 1460 1480 1500 1520 1540 1560 1580 1600 1620 1640 1660 1680 1700 1720 1740 1760 1780 1800 1820 1840 1860 1880 1900 1920 1940 1960
Time (sec)

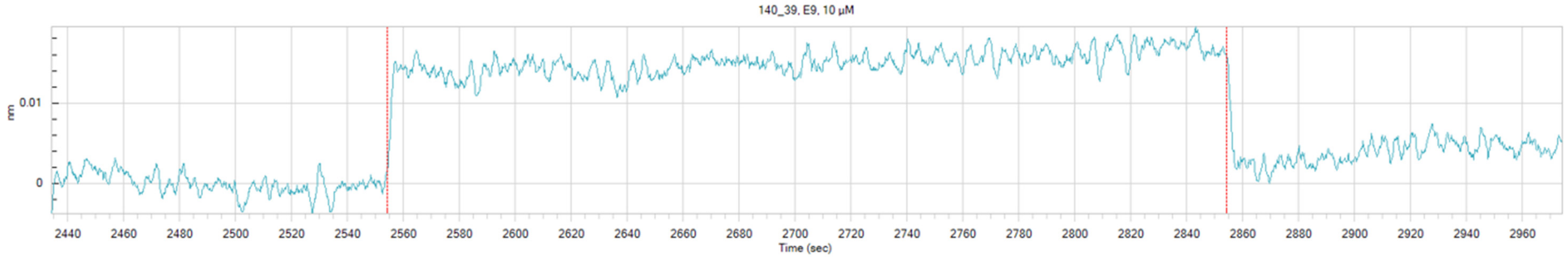
140_39, E9, 10 µM
nm
0.01
0
2440 2460 2480 2500 2520 2540 2560 2580 2600 2620 2640 2660 2680 2700 2720 2740 2760 2780 2800 2820 2840 2860 2880 2900 2920 2940 2960
Time (sec)

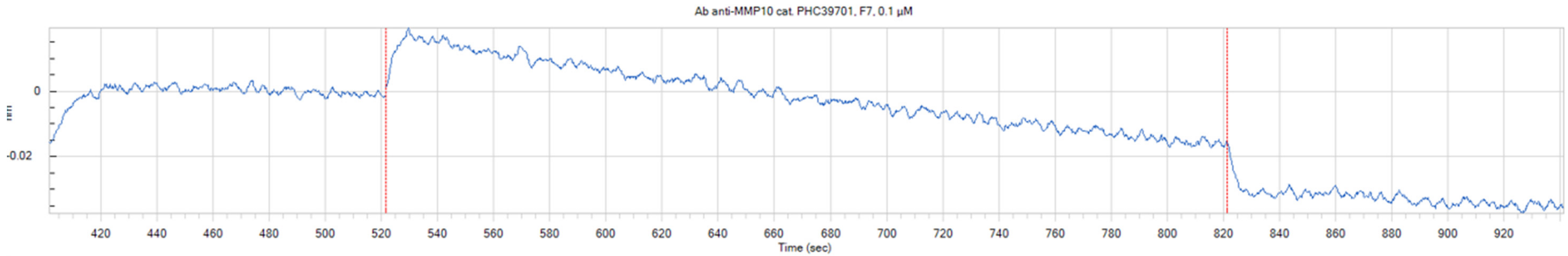
Ab anti-MMP10 cat. PHC39701, F7, 0.1 µM
0
-0.02
420 440 460 480 500 520 540 560 580 600 620 640 660 680 700 720 740 760 780 800 820 840 860 880 900 920
Time (sec)

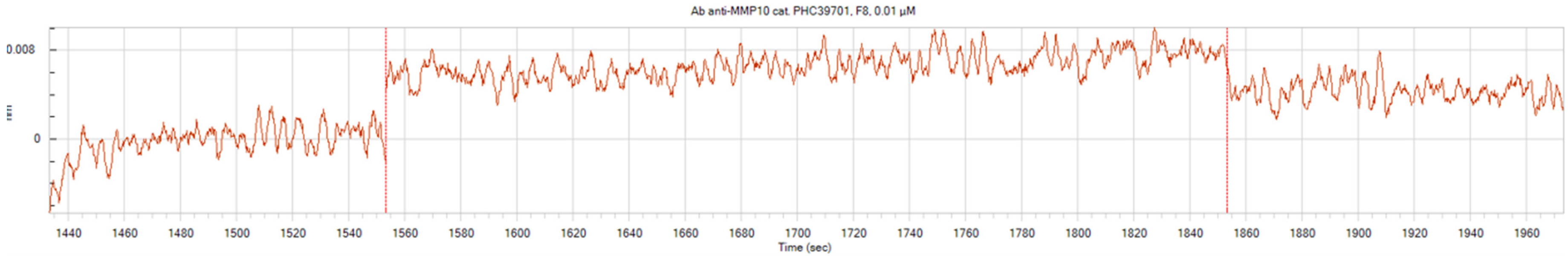
Ab anti-MMP10 cat. PHC39701, F8, 0.01 µM
0.008
0
1440 1460 1480 1500 1520 1540 1560 1580 1600 1620 1640 1660 1680 1700 1720 1740 1760 1780 1800 1820 1840 1860 1880 1900 1920 1940 1960
Time (sec)

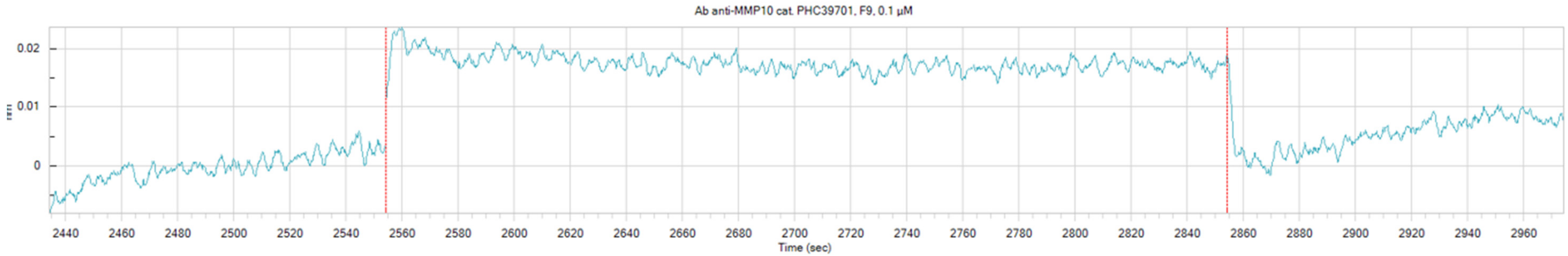
Ab anti-MMP10 cat. PHC39701, F9, 0.1 µM
0.02
0.01
0
2440 2460 2480 2500 2520 2540 2560 2580 2600 2620 2640 2660 2680 2700 2720 2740 2760 2780 2800 2820 2840 2860 2880 2900 2920 2940 2960
Time (sec)

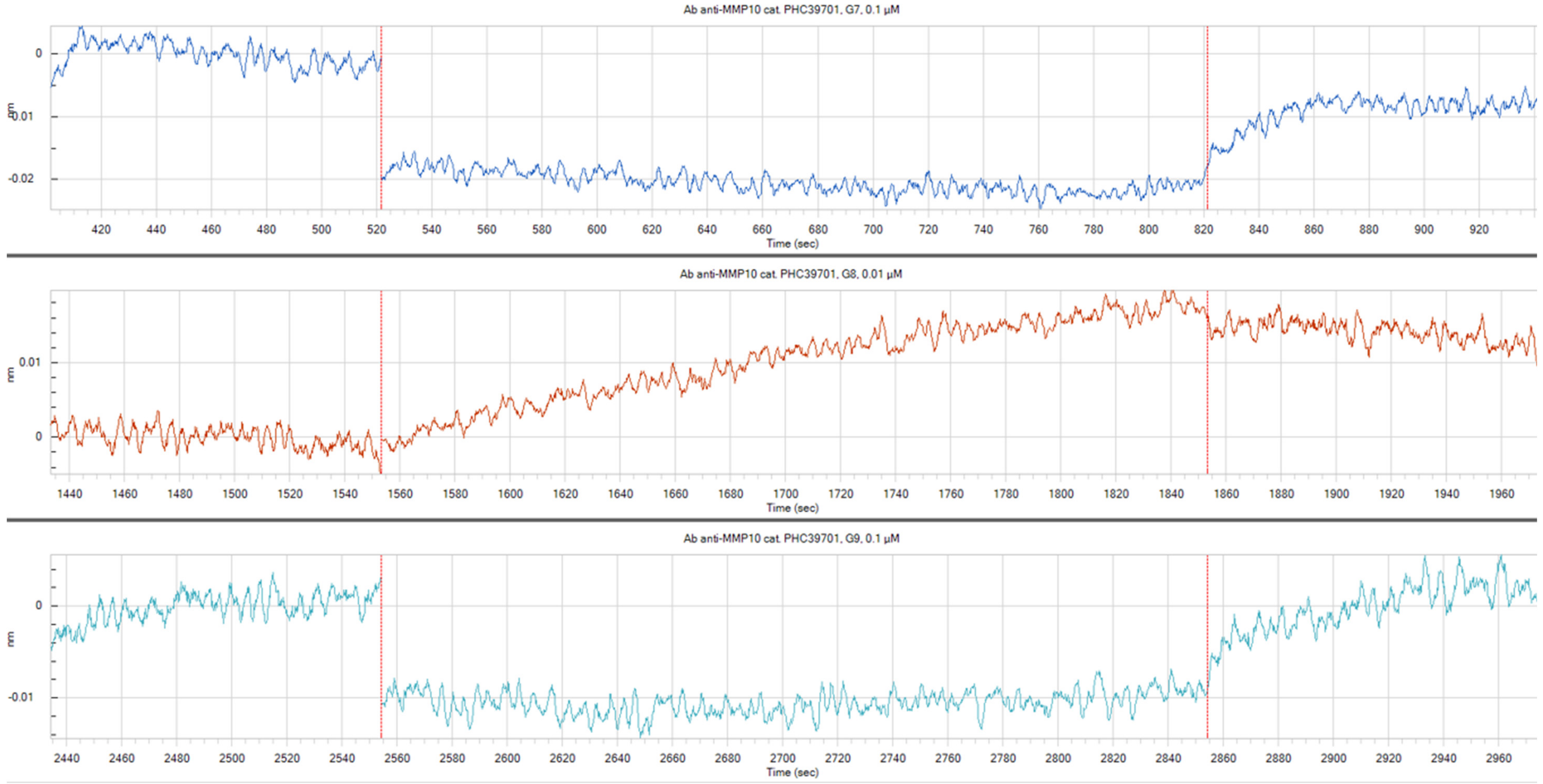

Figure S6: Sensorgrams obtained from analysis on NTA sensors, affinity determination of aptamers against MMP10

7. Analysis on NTA sensors, affinity determination of aptamers against sRAGE.

The sensorgrams in Figure S7 are shown after subtraction of the reference-sensor signal and depict 3 stages of the experiment: baseline establishment, analyte association, and subsequent dissociation, separated by vertical dashed lines. The aptamer code or antibody name, well number, and tested analyte concentration are indicated. Sensorgrams are shown at different scales; the response in nm is indicated on the Y axis. For comparison, sensorgrams obtained for the control antibody are shown, as well as for buffer without analyte.

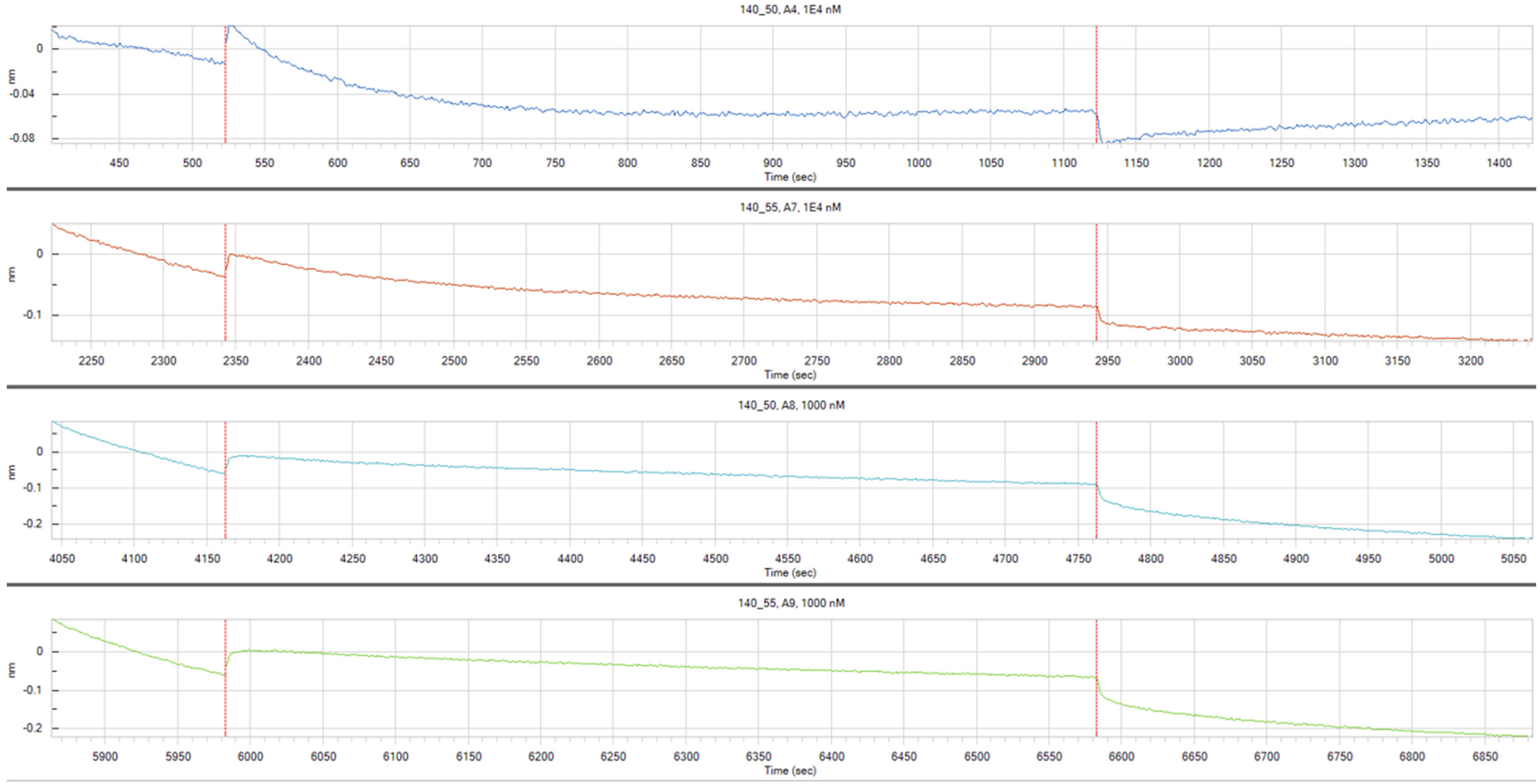

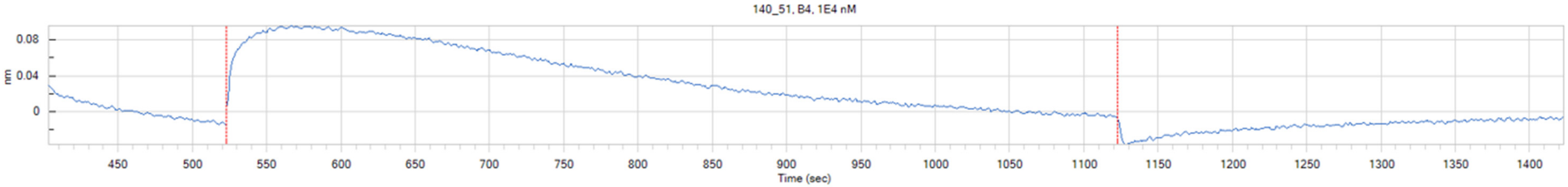
140_51, B4, 1E4 nM
nm
0.08
0.04
0
450 500 550 600 650 700 750 800 850 900 950 1000 1050 1100 1150 1200 1250 1300 1350 1400
Time (sec)

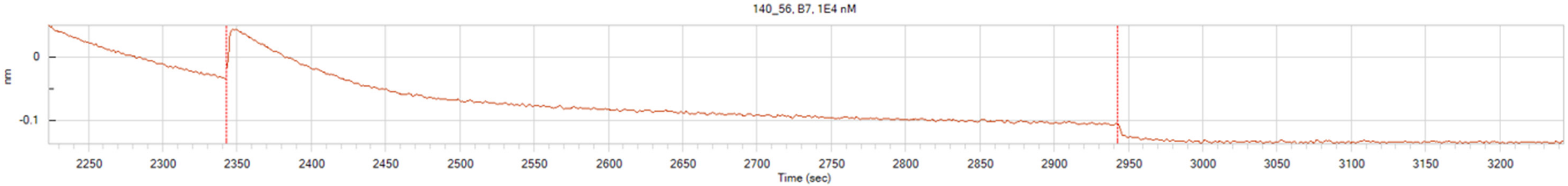
140_56, B7, 1E4 nM
nm
0
-0.1
2250 2300 2350 2400 2450 2500 2550 2600 2650 2700 2750 2800 2850 2900 2950 3000 3050 3100 3150 3200
Time (sec)

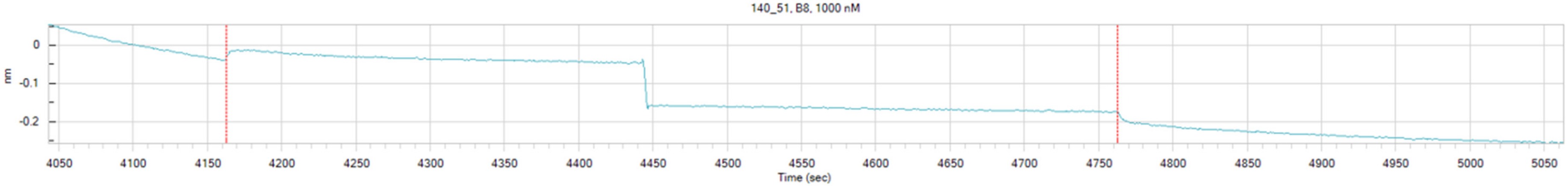
140_51, B8, 1000 nM
nm
0
-0.1
-0.2
4050 4100 4150 4200 4250 4300 4350 4400 4450 4500 4550 4600 4650 4700 4750 4800 4850 4900 4950 5000 5050
Time (sec)

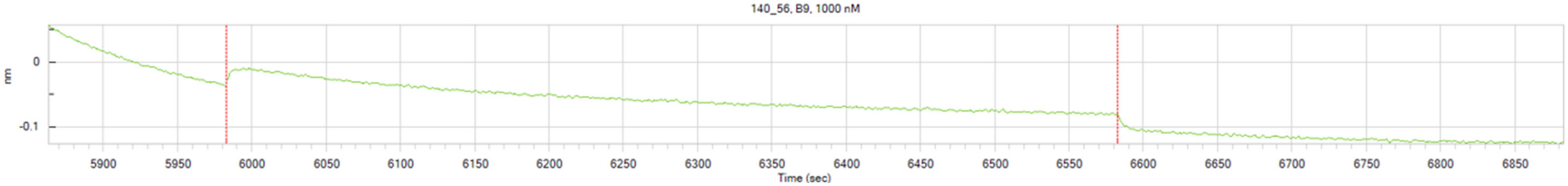
140_56, B9, 1000 nM
nm
0
-0.1
5900 5950 6000 6050 6100 6150 6200 6250 6300 6350 6400 6450 6500 6550 6600 6650 6700 6750 6800 6850
Time (sec)

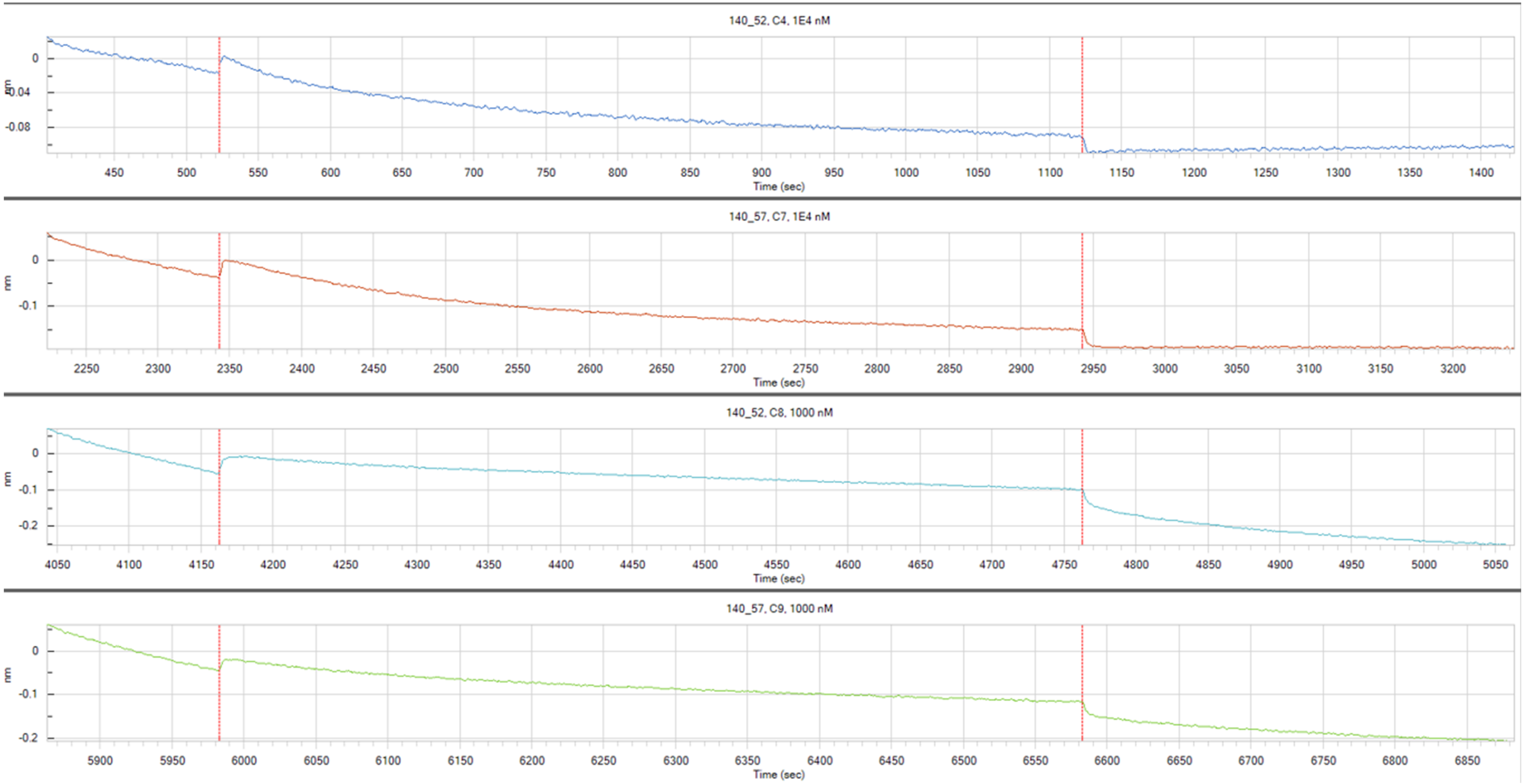
140_52, C4, 1E4 nM
0
-0.04
-0.08
nm
450 500 550 600 650 700 750 800 850 900 950 1000 1050 1100 1150 1200 1250 1300 1350 1400
Time (sec)
140_57, C7, 1E4 nM
0
-0.1
nm
2250 2300 2350 2400 2450 2500 2550 2600 2650 2700 2750 2800 2850 2900 2950 3000 3050 3100 3150 3200
Time (sec)
140_52, C8, 1000 nM
0
-0.1
-0.2
nm
4050 4100 4150 4200 4250 4300 4350 4400 4450 4500 4550 4600 4650 4700 4750 4800 4850 4900 4950 5000 5050
Time (sec)
140_57, C9, 1000 nM
0
-0.1
-0.2
nm
5900 5950 6000 6050 6100 6150 6200 6250 6300 6350 6400 6450 6500 6550 6600 6650 6700 6750 6800 6850
Time (sec)

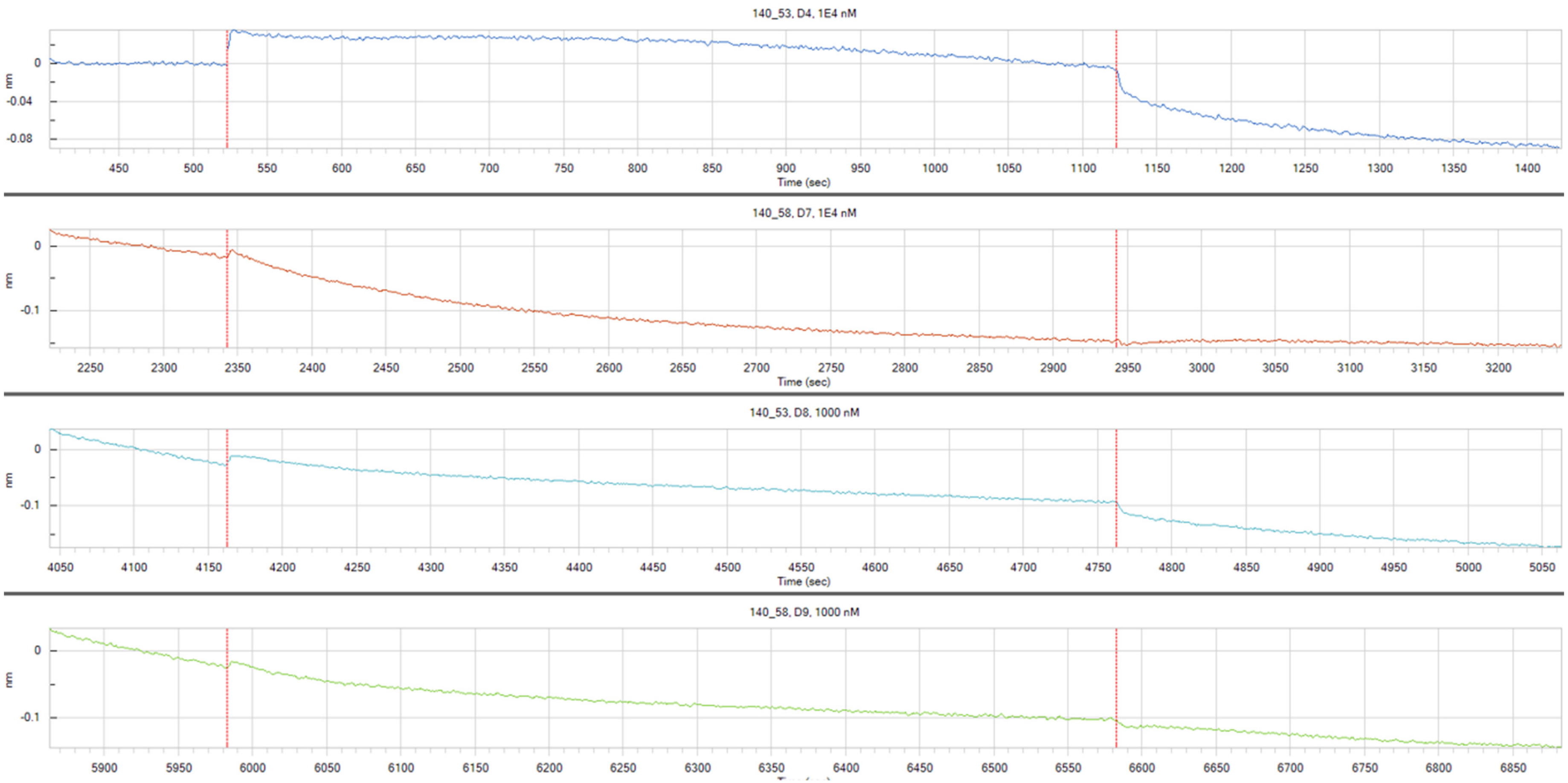
140_53, D4, 1E4 nM
nm
Time (sec)
140_58, D7, 1E4 nM
nm
Time (sec)
140_53, D8, 1000 nM
nm
Time (sec)
140_58, D9, 1000 nM
nm

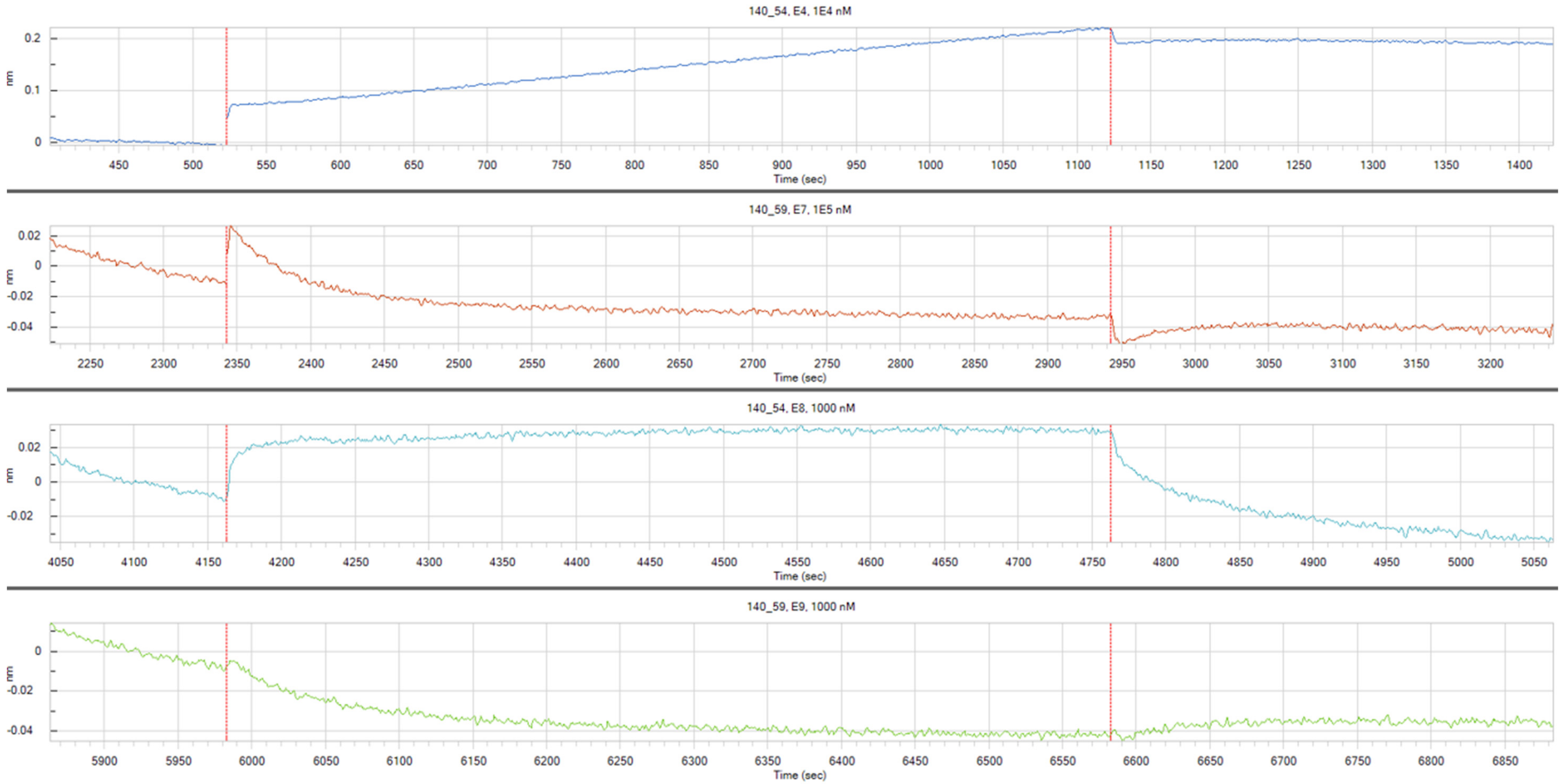
140_54, E4, 1E4 nM
nm
0.2
0.1
0
450
500
550
600
650
700
750
800
850
900
950
1000
1050
1100
1150
1200
1250
1300
1350
1400
Time (sec)
140_59, E7, 1E5 nM
nm
0.02
0
-0.02
-0.04
2250
2300
2350
2400
2450
2500
2550
2600
2650
2700
2750
2800
2850
2900
2950
3000
3050
3100
3150
3200
Time (sec)
140_54, E8, 1000 nM
nm
0.02
0
-0.02
4050
4100
4150
4200
4250
4300
4350
4400
4450
4500
4550
4600
4650
4700
4750
4800
4850
4900
4950
5000
5050
Time (sec)
140_59, E9, 1000 nM
nm
0
-0.02
-0.04
5900
5950
6000
6050
6100
6150
6200
6250
6300
6350
6400
6450
6500
6550
6600
6650
6700
6750
6800
6850
Time (sec)

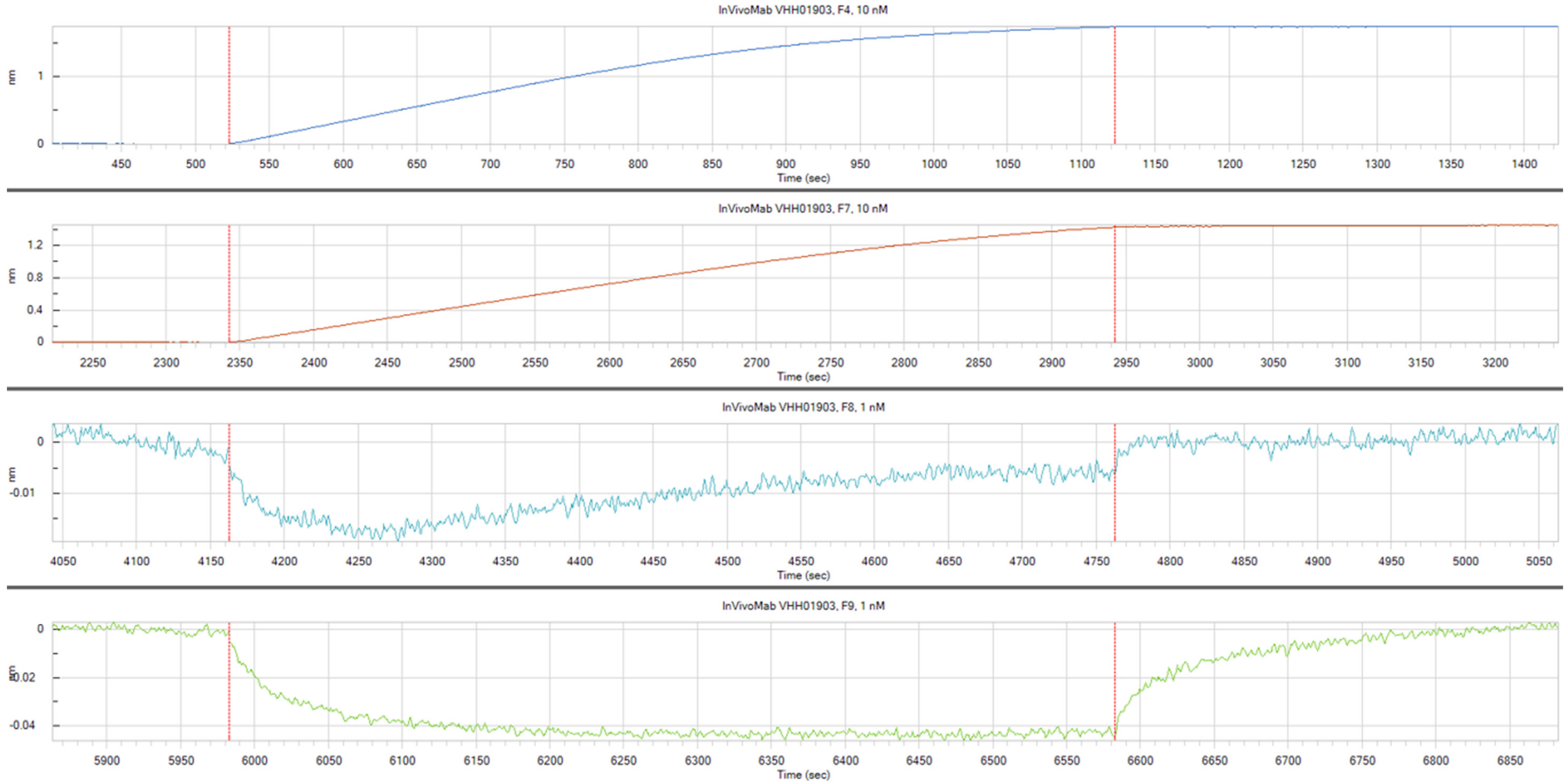
InVivoMab VHH01903, F4, 10 nM
nm
Time (sec)
InVivoMab VHH01903, F7, 10 nM
nm
Time (sec)
InVivoMab VHH01903, F8, 1 nM
nm
Time (sec)
InVivoMab VHH01903, F9, 1 nM
nm
Time (sec)

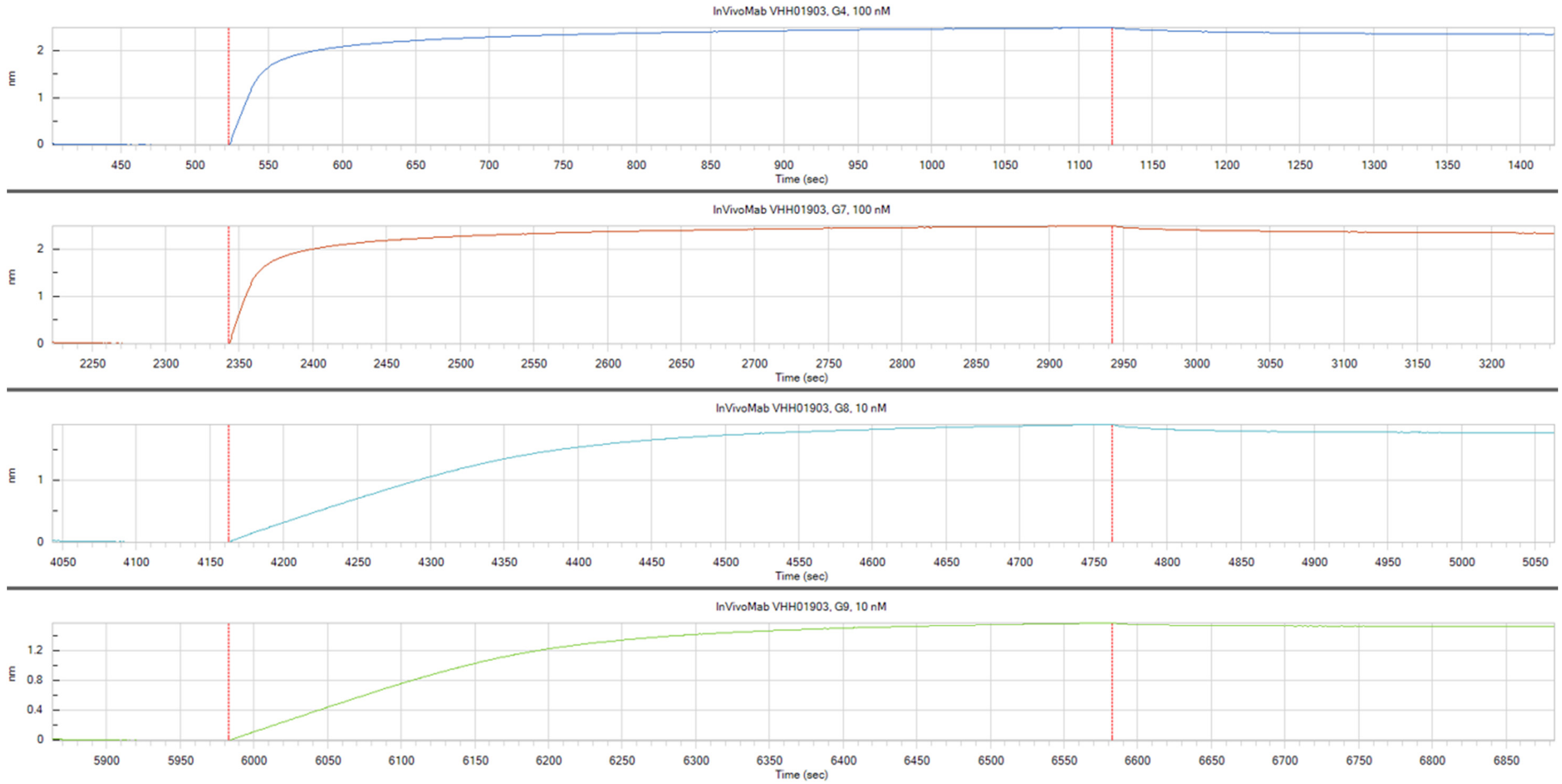
InVivoMab VHH01903, G4, 100 nM
nm
Time (sec)
InVivoMab VHH01903, G7, 100 nM
nm
Time (sec)
InVivoMab VHH01903, G8, 10 nM
nm
Time (sec)
InVivoMab VHH01903, G9, 10 nM
nm
Time (sec)

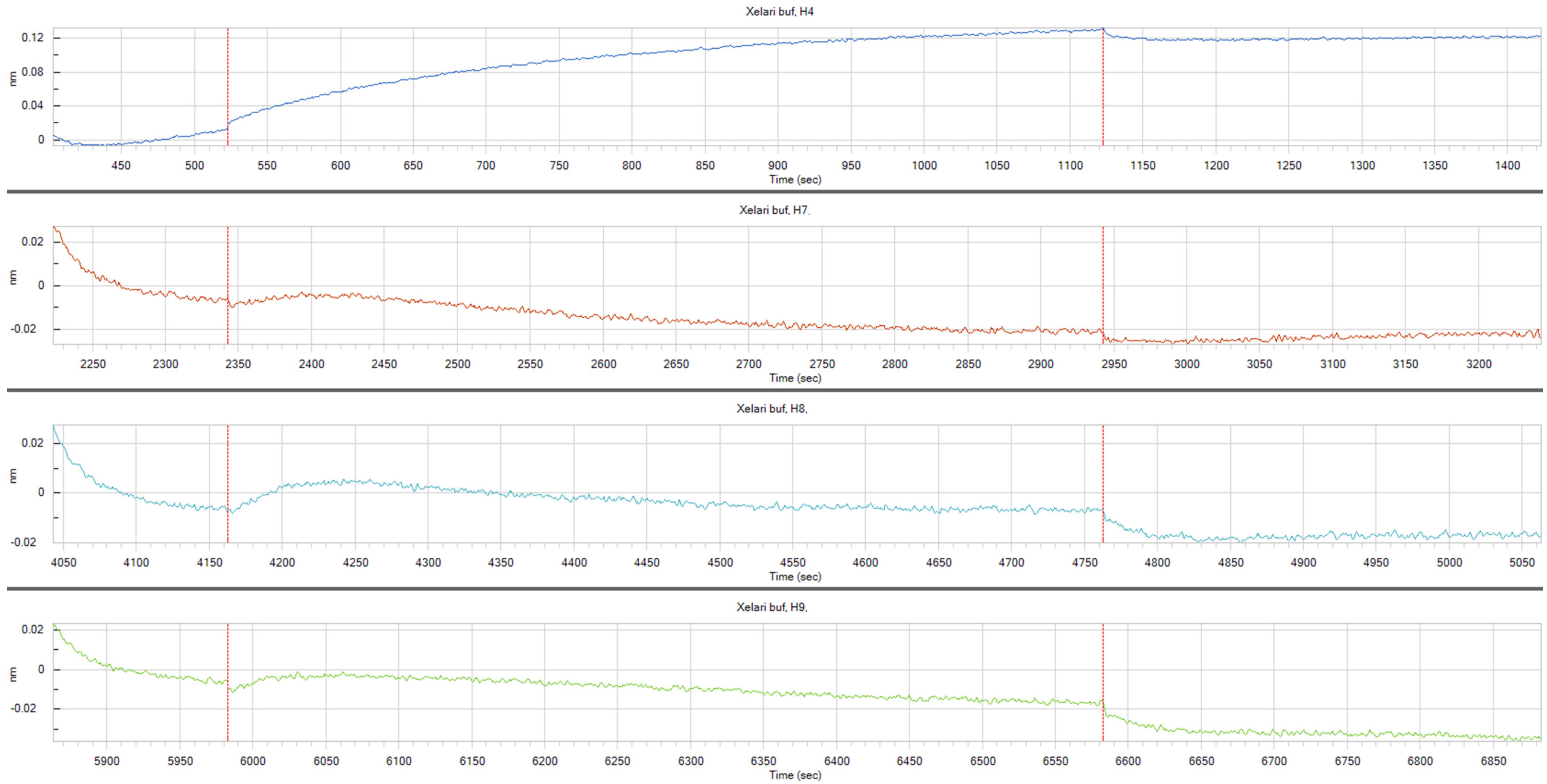

Figure S7: Sensorgrams obtained from analysis on NTA sensors, affinity determination of aptamers against sRAGE

8. Analysis on NTA sensors, affinity determination of aptamers against ESM1.

The sensorgrams in Figure S8 are shown after subtraction of the reference-sensor signal and depict 3 stages of the experiment: baseline establishment, analyte association, and subsequent dissociation, separated by vertical dashed lines. The aptamer code or antibody name, well number, and tested analyte concentration are indicated. Sensorgrams are shown at different scales; the response in nm is indicated on the Y.

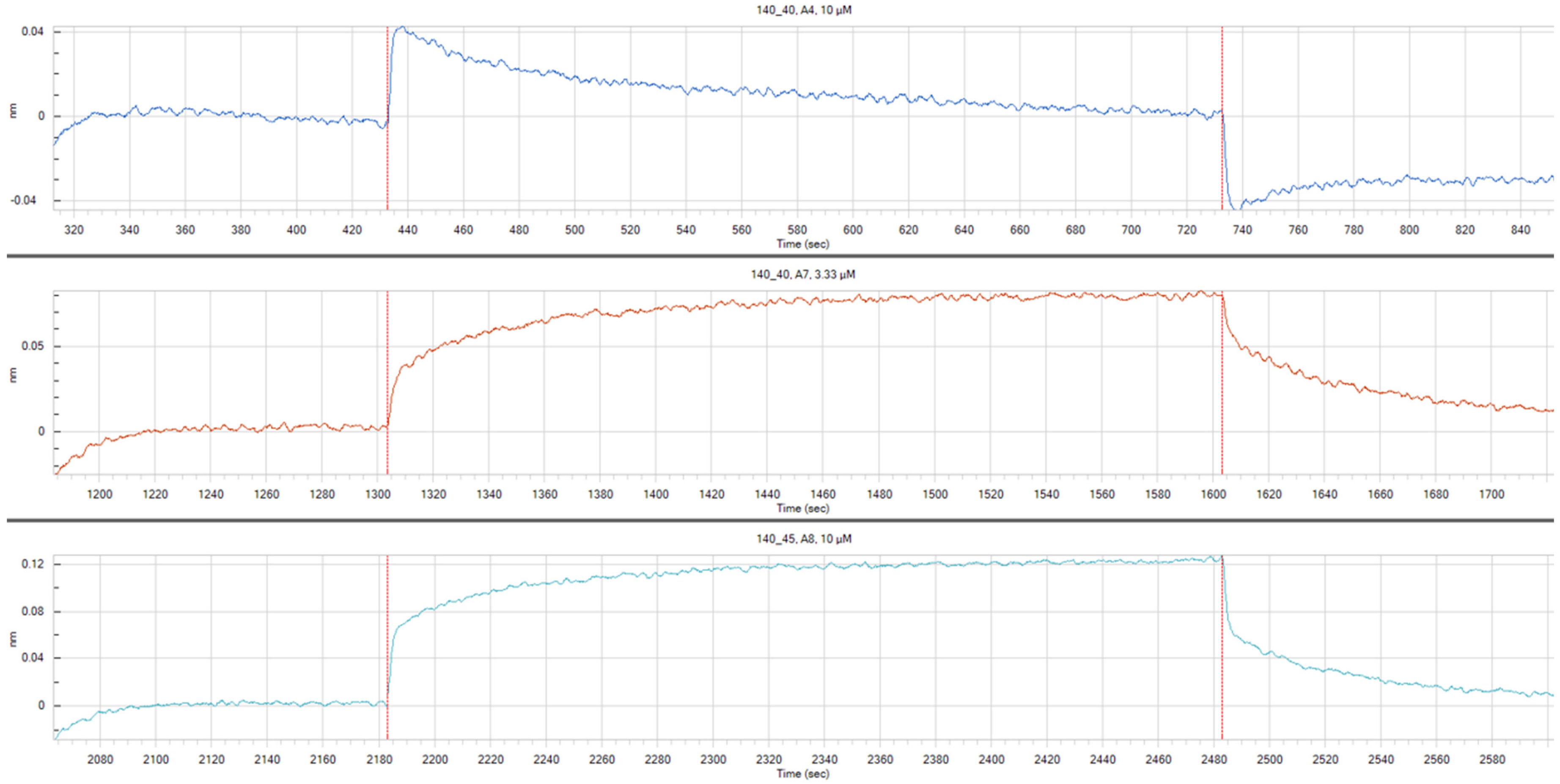

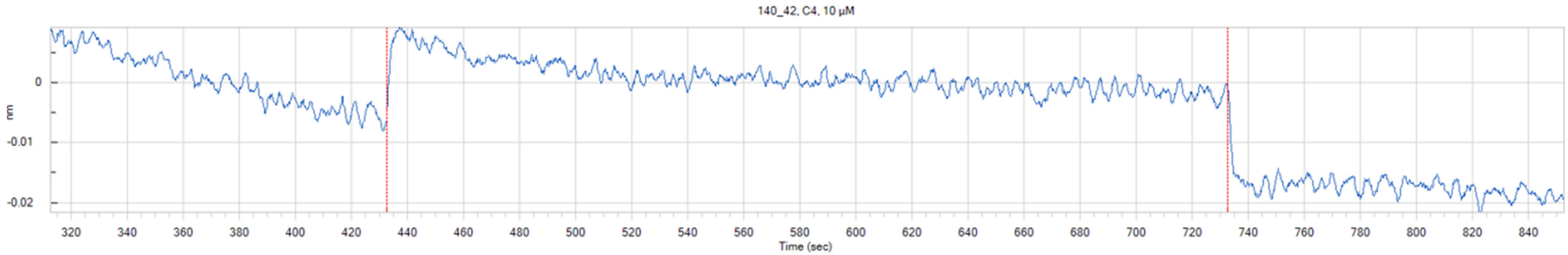
140_42, C4, 10 µM
nm
0
-0.01
-0.02
320 340 360 380 400 420 440 460 480 500 520 540 560 580 600 620 640 660 680 700 720 740 760 780 800 820 840
Time (sec)

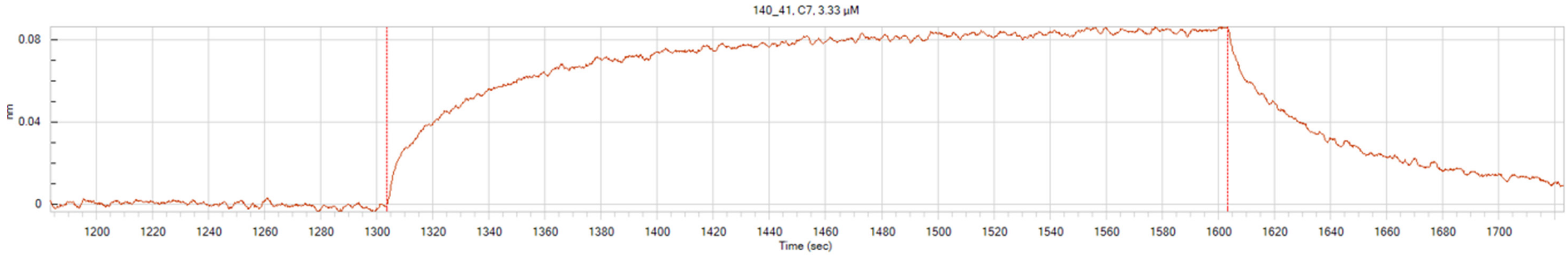
140_41, C7, 3.33 µM
nm
0.08
0.04
0
1200 1220 1240 1260 1280 1300 1320 1340 1360 1380 1400 1420 1440 1460 1480 1500 1520 1540 1560 1580 1600 1620 1640 1660 1680 1700
Time (sec)

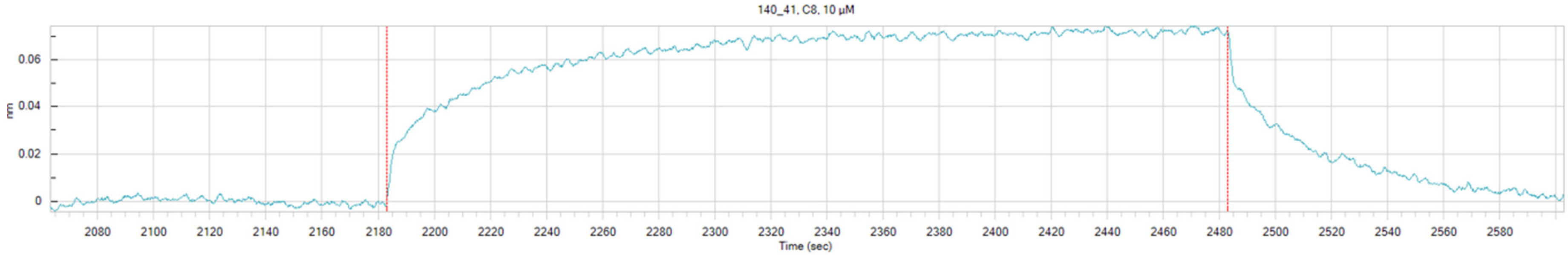
140_41, C8, 10 µM
nm
0.06
0.04
0.02
0
2080 2100 2120 2140 2160 2180 2200 2220 2240 2260 2280 2300 2320 2340 2360 2380 2400 2420 2440 2460 2480 2500 2520 2540 2560 2580
Time (sec)

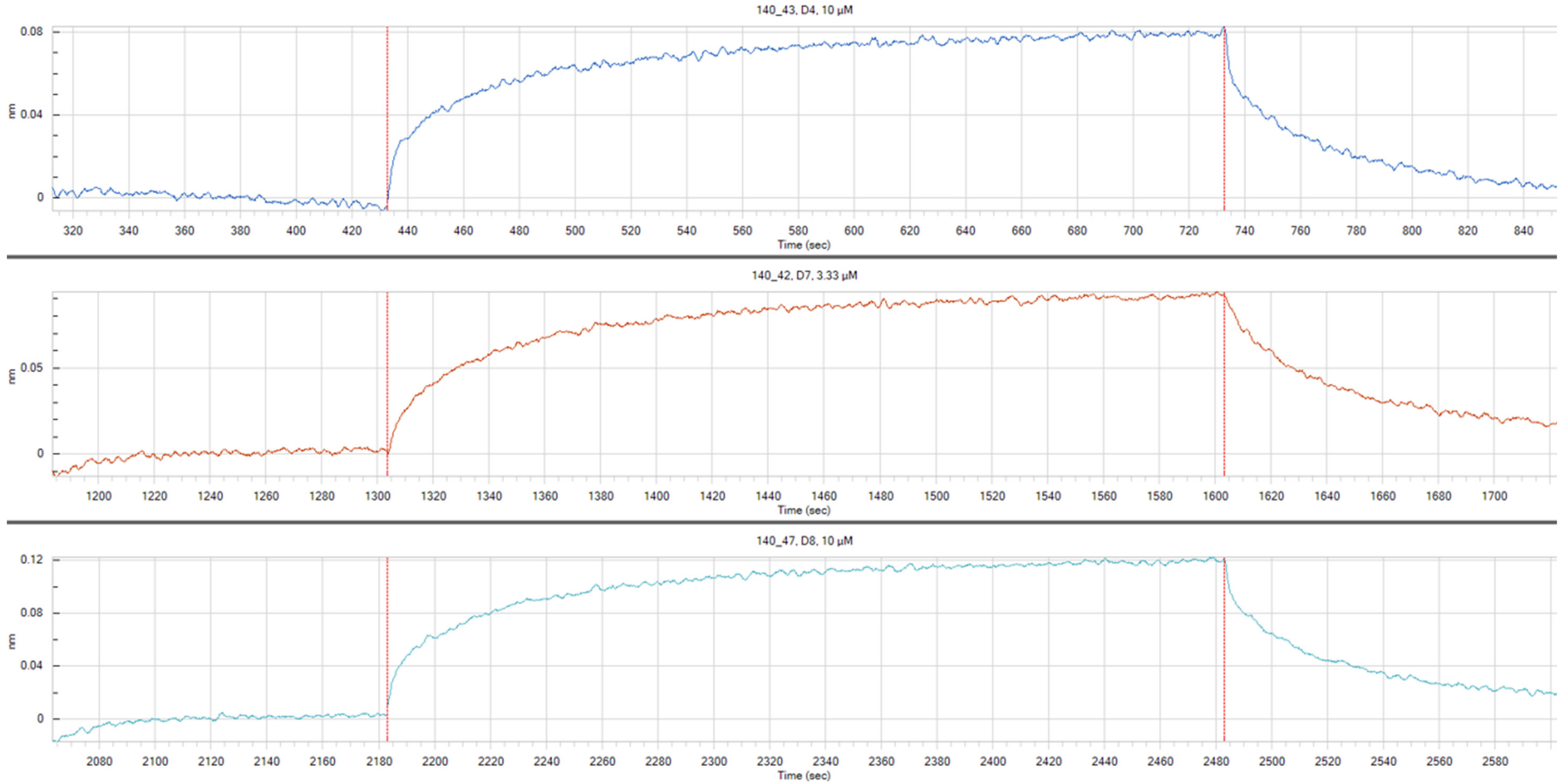
140_43, D4, 10 µM
nm
Time (sec)
140_42, D7, 3.33 µM
nm
Time (sec)
140_47, D8, 10 µM
nm
Time (sec)

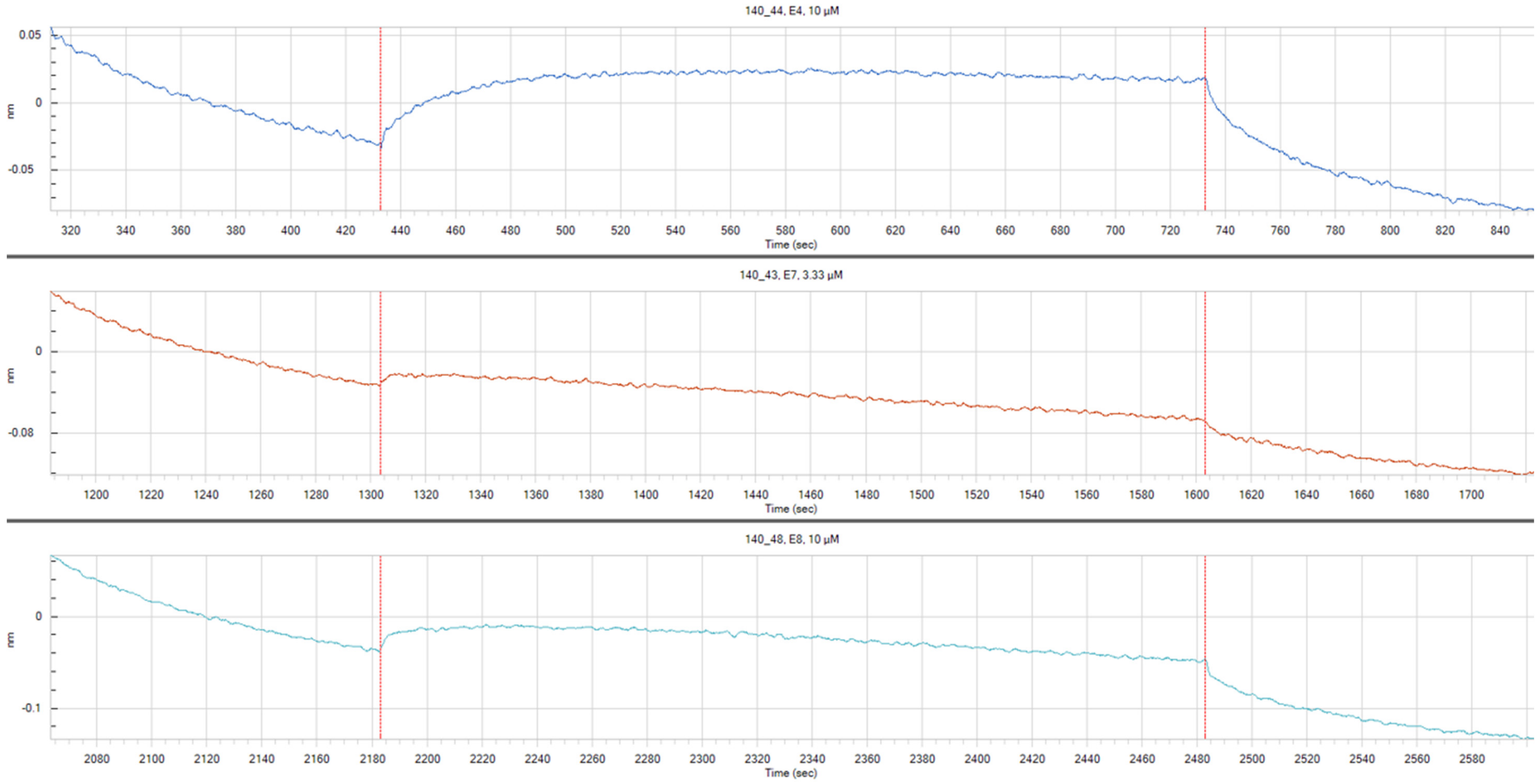
140_44, E4, 10 µM
0.05
0
-0.05
nm
320
340
360
380
400
420
440
460
480
500
520
540
560
580
600
620
640
660
680
700
720
740
760
780
800
820
840
Time (sec)
140_43, E7, 3.33 µM
0
-0.08
nm
1200
1220
1240
1260
1280
1300
1320
1340
1360
1380
1400
1420
1440
1460
1480
1500
1520
1540
1560
1580
1600
1620
1640
1660
1680
1700
Time (sec)
140_48, E8, 10 µM
0
-0.1
nm
2080
2100
2120
2140
2160
2180
2200
2220
2240
2260
2280
2300
2320
2340
2360
2380
2400
2420
2440
2460
2480
2500
2520
2540
2560
2580
Time (sec)

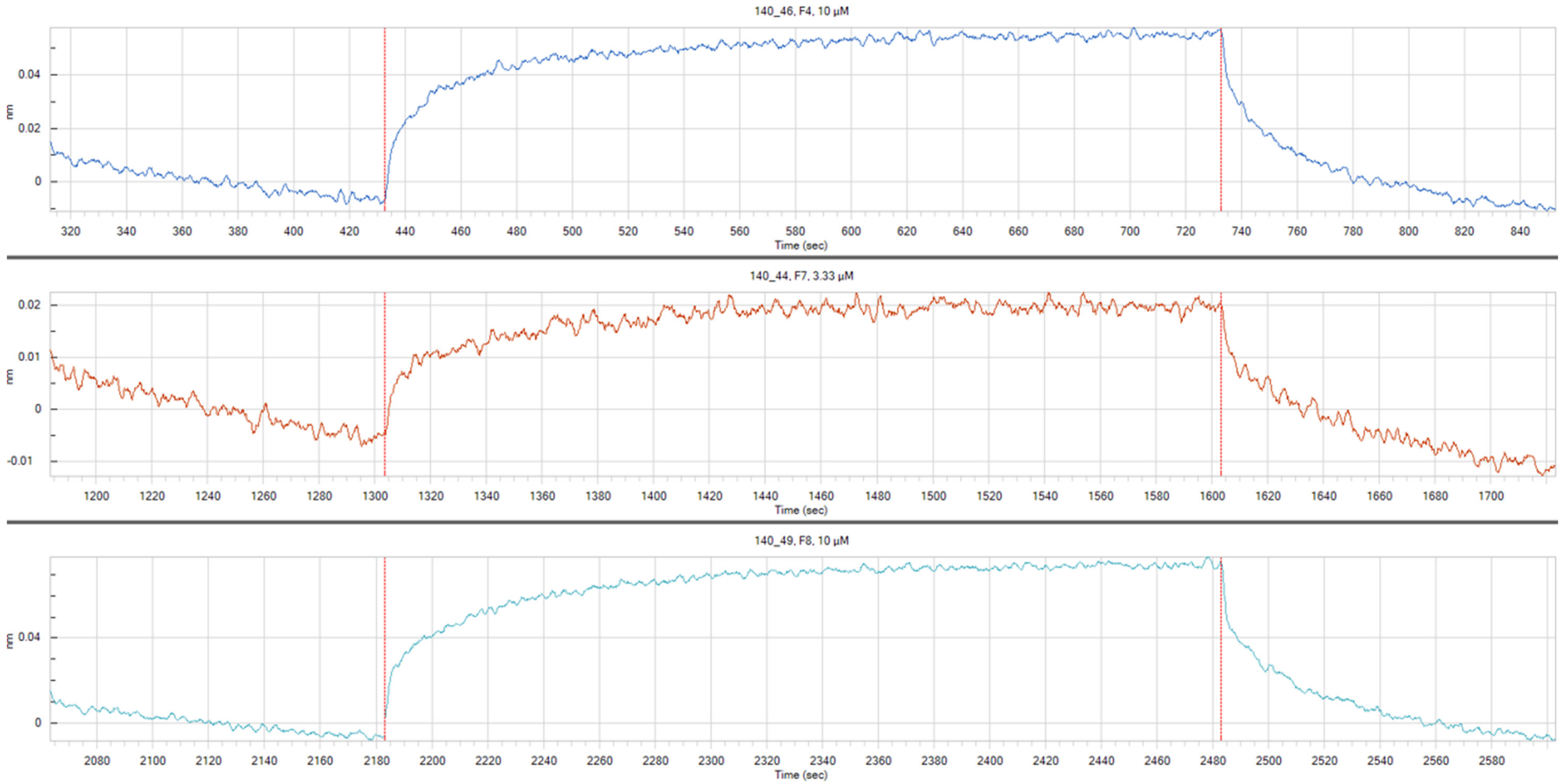

Figure S8: Sensorgrams obtained from analysis on NTA sensors, affinity determination of aptamers against ESM1

9. Analysis on SAX2 sensors, affinity determination of aptamers against MMP2.

Performing BLI analysis on SAX2 sensors

The sensorgrams in Figure S9 are shown after subtraction of the reference-sensor signal (a sensor not loaded with aptamers but exposed to the target protein at the same concentration as the aptamer-loaded sensors) and depict 2 stages of the experiment: analyte association and subsequent dissociation, separated by vertical dashed lines. The aptamer code and tested analyte concentration are indicated. Sensorgrams are shown at different scales; the response in nm is indicated on the Y axis. This test was performed at the Customer's request, beyond the scope of the original technical assignment, on the reasoning that a protein of ~60-70 kDa used as the analyte gives a more noticeable sensorgram response than an aptamer used as the analyte; a response comparable to, or at most ~2-fold weaker than, that of antibodies was expected. However, the expected response and characteristic association-dissociation curves were not obtained, and accordingly KD was not calculated; for 2 of the 3 tested concentrations the response observed was <0.01 nm, at the noise level, which makes global-fit calculation of KD using sensorgrams for 3 or more analyte concentrations unfeasible by design. For aptamer 161_16, no sensorgram was obtained when testing with 253.8 nM MMP2 due to a sensor failure at this step; however, the absence of a response exceeding the instrumental noise level, and of a curve shape characteristic of affinity binding, at a concentration of MMP2 of 84.6 nM suggests that KD for this aptamer, as for the others, exceeds 84.6 nM and, a fortiori, 70 nM.

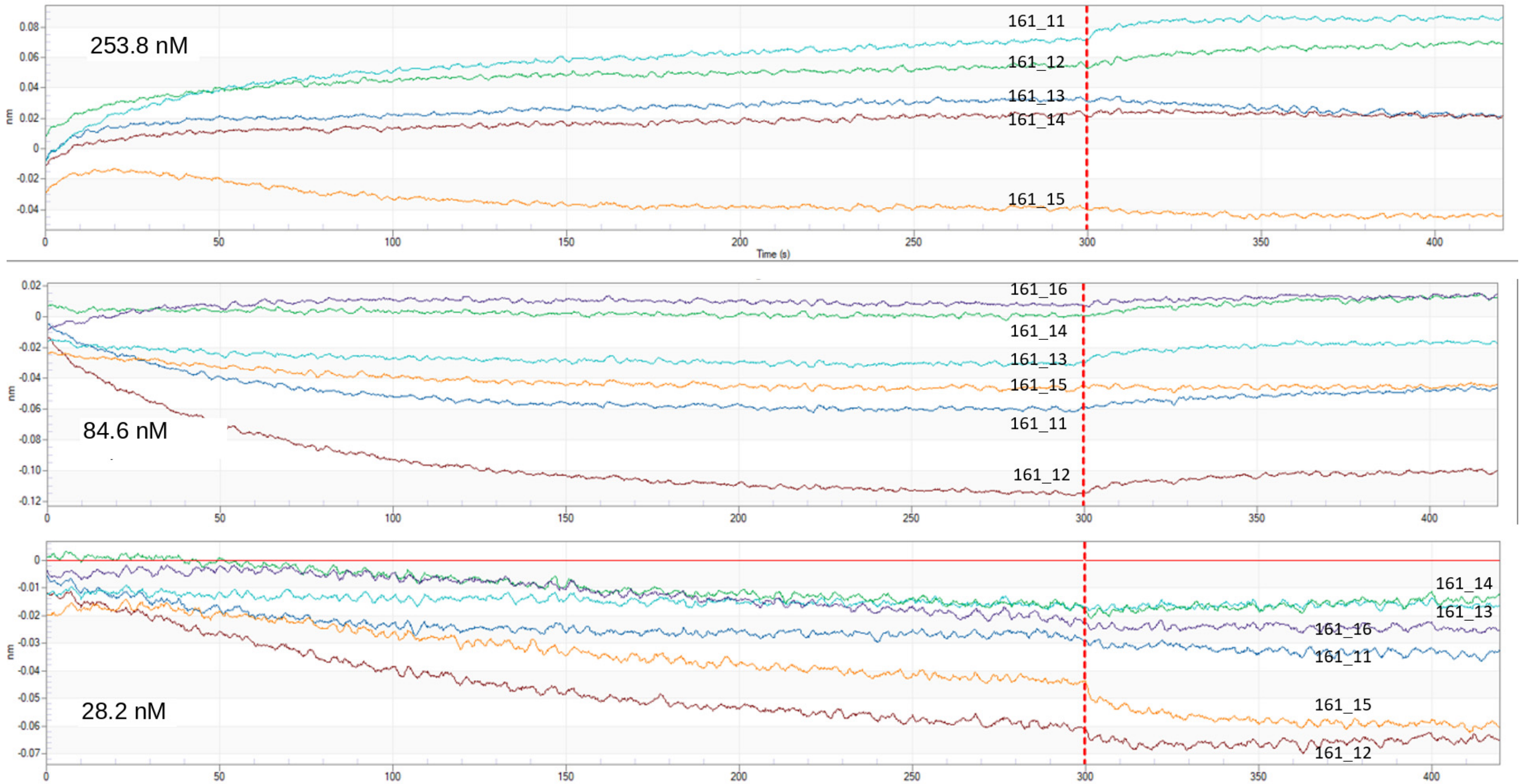

Figure S9: Sensorgrams obtained from analysis on SAX2 sensors, affinity determination of aptamers against MMP2

10. Analysis on SAX2 sensors, affinity determination of aptamers against PCSK9 (SBT).

Sensorgrams for aptamers against PCSK9 (SBT) on SAX2 sensors are shown after subtraction of the reference-sensor signal (a sensor not loaded with aptamers but exposed to the target protein at the same concentration as the aptamer-loaded sensors) and depict 2 stages of the experiment: analyte association and subsequent dissociation. An example of signal subtraction and the resulting sensorgram is shown in Figure S10. The resulting sensorgrams for the tested aptamers are shown in Figures S11 and S12. Stages are separated by vertical dashed lines. The aptamer code and tested analyte concentration are indicated. Note the extremely low response level for the testing of all aptamers at a concentration of PCSK9 of 111.1 nM, exceeding the suitability criterion for KD per the technical assignment (70 nM); accordingly, global-fit KD calculation using sensorgrams for 3 or more analyte concentrations was not performed. As in the previous experiment, for aptamer 161_6 no sensorgram was obtained due to sensor failure at the tested PCSK9 concentrations of 1000 and 333.3 nM; however, from the absence of a response exceeding the instrumental noise level for this aptamer at the tested PCSK9 concentration of 111.1 nM, it can be assumed that KD for this aptamer exceeds 111.1 nM and, a fortiori, 70 nM.

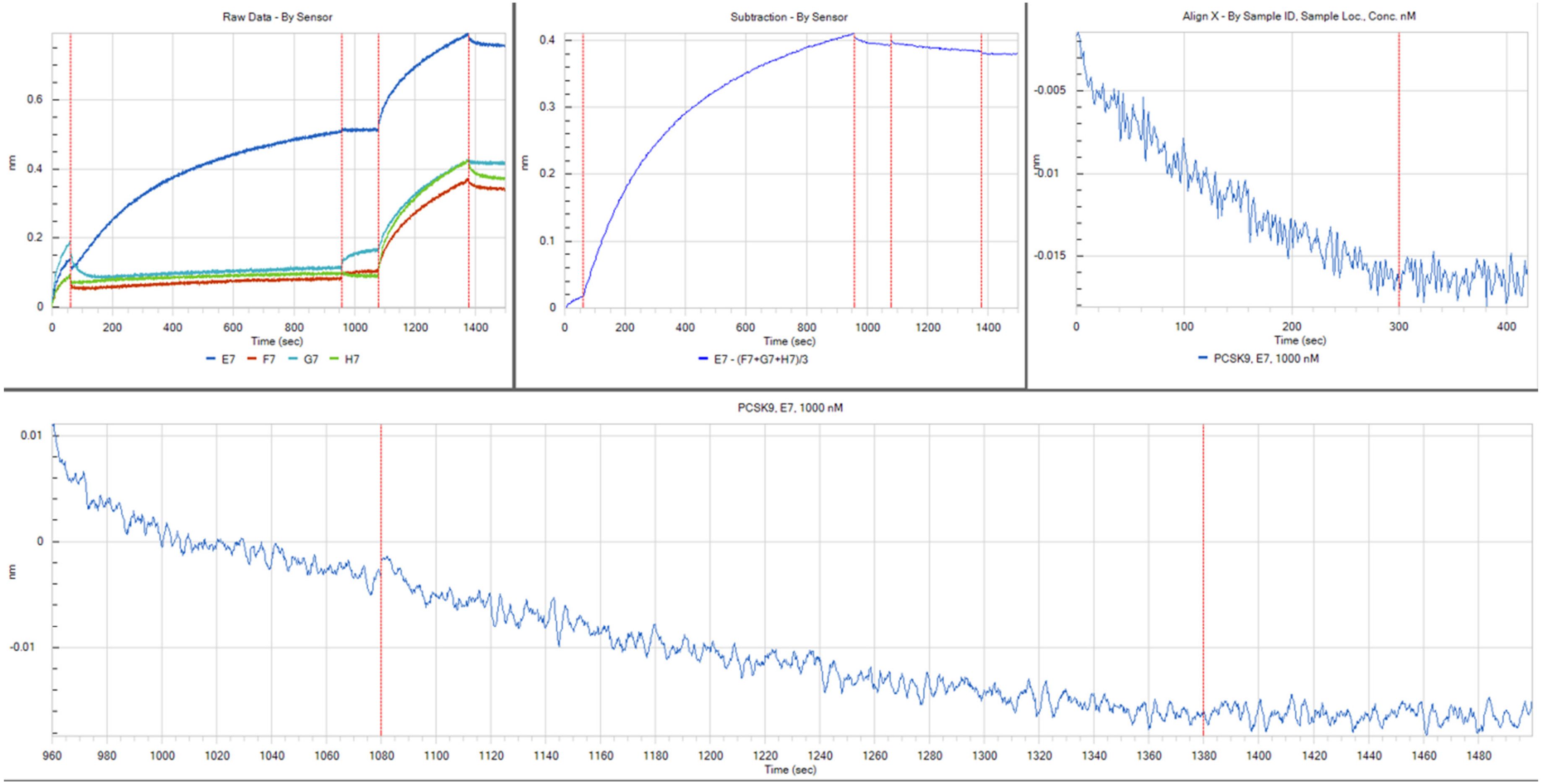

Figure S10: Example sensorgram obtained by subtracting the averaged nonspecific signal from the reference sensors (sensors F7, G7, H7) from the signal of the sensor loaded with aptamer 161_10 (sensor E7) during analysis on SAX2 sensors, affinity determination of aptamers against PCSK9 (SBT)

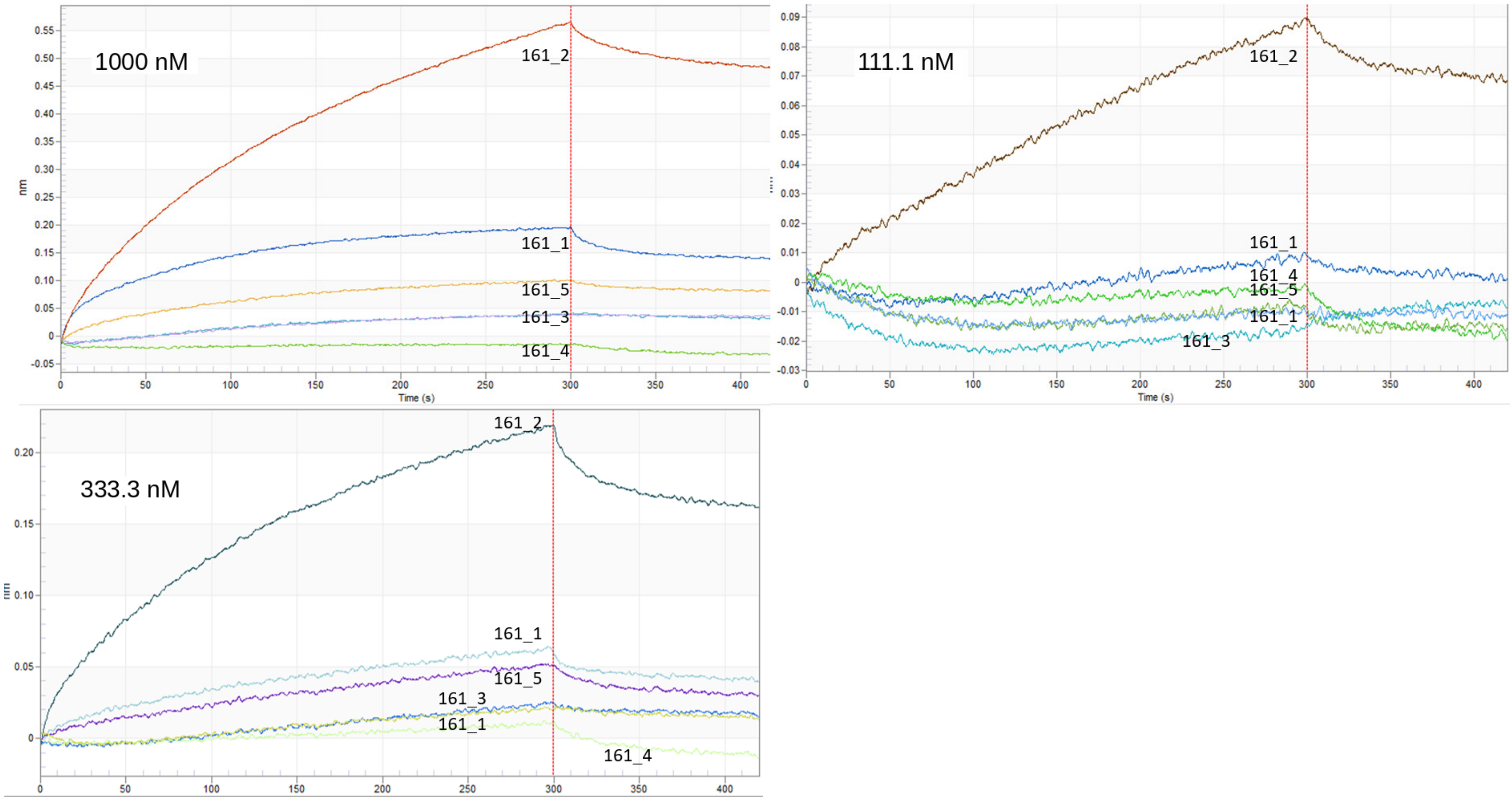

Figure S11: Sensorgrams obtained from analysis on SAX2 sensors, affinity determination of aptamers 161_1 through 161_5 against PCSK9 (SBT)

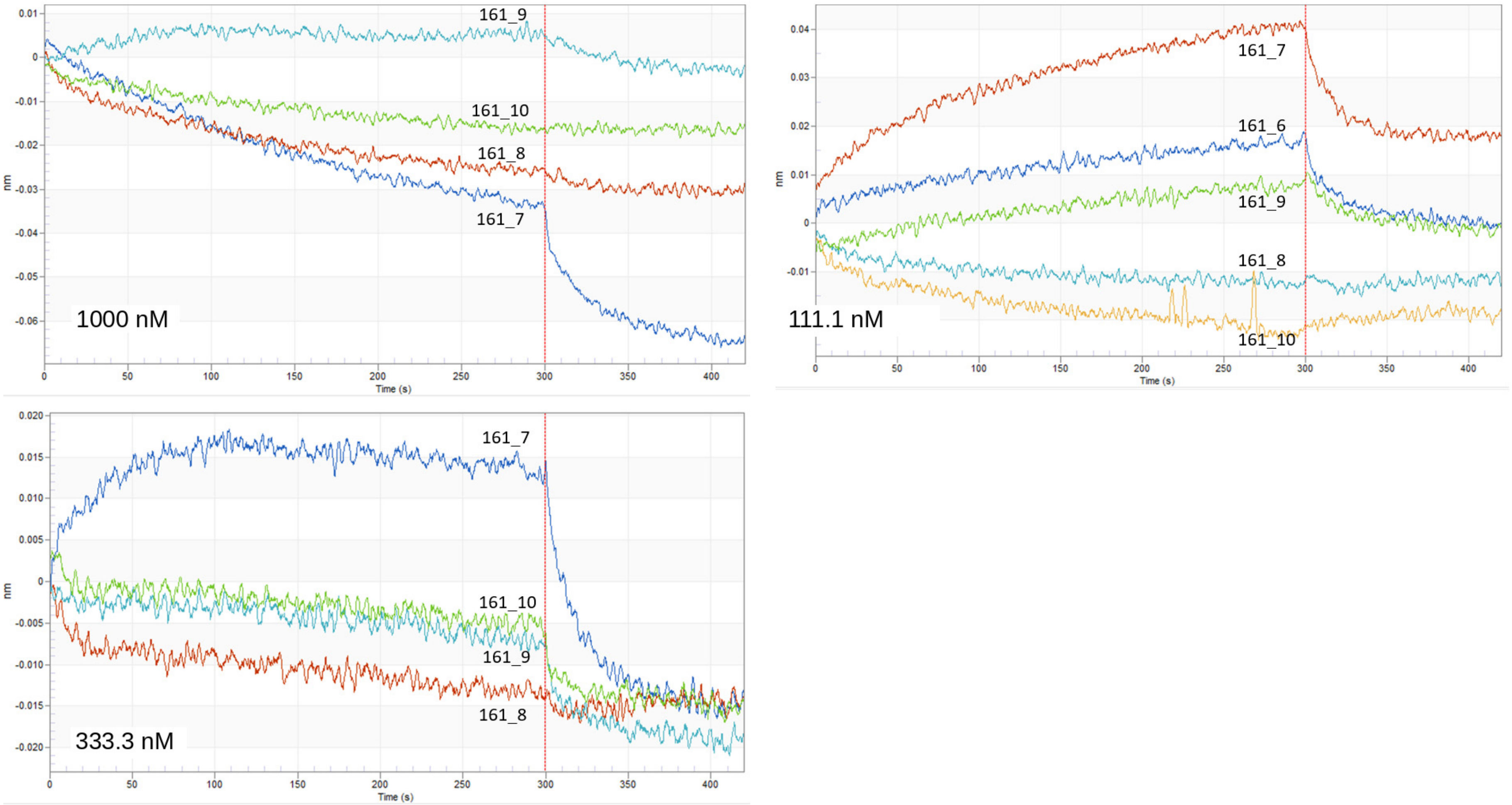

Figure S12: Sensorgrams obtained from analysis on SAX2 sensors, affinity determination of aptamers 161_6 through 161_10 against PCSK9 (SBT)

Supplementary Information S3

Quantitative characteristics of aptamer–target protein interactions from BLI analysis

Quantitative characteristics of aptamer–target protein interactions from BLI analysis on NTA sensors (Tables S3–S10)

This section presents the data obtained from BLI analysis on NTA sensors, sorted by the response in nm reached on the sensorgram during the analyte association step (after subtraction of the reference-sensor signal). Because determining affinity by the BLI method is recommended only for a response of at least 0.1–0.2 nm, well above the instrumental noise level, data meeting this criterion are highlighted. This supplement also reports the calculation of K_D under relaxed requirements, for cases where the response exceeds 0.05 nm. Data with a response <0.05 nm cannot reliably be used to calculate K_D because instrumental noise can reach 0.01 nm or more (see Figures S1–S8 in Supplementary Information S2); the corresponding columns are therefore marked ND. Binding constants are given in exponential format, 1E-12 M corresponds to 1 pM, etc.

Table S3: Analysis on NTA sensors, affinity determination of aptamers against IL6

Sample	Conc. (µM)	Response	KD (M)	KD Error	kon(1/Ms)	kon Error	kdis(1/s)	kdis Error
Ab SAA0375 FHC15810	0.1	1.0542	<1.0E-12	7.24E-11	3.19E+05	7.08E+03	<1.0E-07	
Ab SAA0375 FHC15810	0.1	1.0081	<1.0E-12	7.66E-11	2.90E+05	5.93E+03	<1.0E-07	
Ab SAA0375 FHC15810	0.01	0.7915	3.12E-11	4.14E-12	6.76E+05	1.26E+03	2.11E-05	2.80E-06
Ab SAA0375 FHC15810	0.01	0.5603	<1.0E-12	5.20E-12	7.73E+05	2.03E+03	<1.0E-07	
140_1	1	0.0561	5.97E-07	2.36E-08	2.08E+03	7.03E+01	1.24E-03	2.56E-05
140_4	1	0.0479	ND	ND	ND	ND	ND	ND
140_8	10	0.0428	ND	ND	ND	ND	ND	ND
140_3	1	0.0339	ND	ND	ND	ND	ND	ND
140_6	10	0.0321	ND	ND	ND	ND	ND	ND
140_9	10	0.0312	ND	ND	ND	ND	ND	ND
140_3	10	0.0287	ND	ND	ND	ND	ND	ND
140_2	1	0.0263	ND	ND	ND	ND	ND	ND
140_5	1	0.02	ND	ND	ND	ND	ND	ND
140_7	10	0.0194	ND	ND	ND	ND	ND	ND
140_2	10	0.0164	ND	ND	ND	ND	ND	ND
140_1	10	0.0148	ND	ND	ND	ND	ND	ND
140_10	1	0.0136	ND	ND	ND	ND	ND	ND
140_10	10	0.0098	ND	ND	ND	ND	ND	ND
140_6	1	0.0086	ND	ND	ND	ND	ND	ND
140_4	10	0.0053	ND	ND	ND	ND	ND	ND
140_8	1	0.0001	ND	ND	ND	ND	ND	ND
140_7	1	-0.0135	ND	ND	ND	ND	ND	ND
140_9	1	-0.0156	ND	ND	ND	ND	ND	ND
140_5	10	-0.0197	ND	ND	ND	ND	ND	ND

Table S4: Analysis on NTA sensors, affinity determination of aptamers against MMP2

Sample ID	Conc. (nM)	Response	KD (M)	KD Error	kon(1/Ms)	kon Error	kdis(1/s)	kdis Error
RHC32301 Anti-Human MMP2 Antibody	100	2.1883	2.78E-09	5.42E-11	4.13E+04	1.55E+02	1.15E-04	2.20E-06
RHC32301 Anti-Human MMP2 Antibody	100	1.8206	4.21E-09	4.91E-11	3.73E+04	1.18E+02	1.57E-04	1.76E-06
140_15	1000	0.019	ND	ND	ND	ND	ND	ND
140_13	1000	0.0185	ND	ND	ND	ND	ND	ND
140_16	1000	0.018	ND	ND	ND	ND	ND	ND
140_19	1000	0.0147	ND	ND	ND	ND	ND	ND
140_11	1000	0.0132	ND	ND	ND	ND	ND	ND
140_12	100	0.0114	ND	ND	ND	ND	ND	ND
140_17	1000	0.0105	ND	ND	ND	ND	ND	ND
140_12	1000	0.0099	ND	ND	ND	ND	ND	ND
140_13	100	0.0096	ND	ND	ND	ND	ND	ND
140_18	1000	0.0087	ND	ND	ND	ND	ND	ND

Table S5: Analysis on NTA sensors, affinity determination of aptamers against PCSK9 (preparation #9, SBT)

Sample ID	Conc. (nM)	Response	KD (M)	KD Error	kon(1/Ms)	kon Error	kdis(1/s)	kdis Error
Evolocumab (Repatha)	200	1.35	<1.0E-12	2.14E-10	8.74E+04	1.23E+03	<1.0E-07	
RMJ24001 Anti-Mouse PCSK9 Antibody (mAb1),AntibodySystem	200	1.0302	<1.0E-12	2.70E-10	1.04E+05	2.33E+03	<1.0E-07	
132_1	1.00E+04	0.0865	5.98E-06	1.37E-07	1.20E+02	2.31E+00	7.14E-04	8.79E-06
132_8	1.00E+04	0.0632	6.91E-04	1.39E-03	1.27E+01	2.55E+01	8.78E-03	1.41E-04
132_9	1.00E+04	0.0597	2.17E-03	2.44E-02	4.89E+00	5.50E+01	1.06E-02	3.19E-04
132_3	1.00E+04	0.0581	6.18E-05	9.39E-06	4.21E+01	6.38E+00	2.60E-03	2.76E-05
132_2	1.00E+04	0.051	3.24E-06	1.67E-07	2.59E+02	9.12E+00	8.38E-04	3.14E-05
132_1	1000	0.046	ND	ND	ND	ND	ND	ND
132_7	1.00E+04	0.0452	ND	ND	ND	ND	ND	ND
132_10	1.00E+04	0.0432	ND	ND	ND	ND	ND	ND
132_6	1.00E+04	0.0323	ND	ND	ND	ND	ND	ND
132_5	1.00E+04	0.0227	ND	ND	ND	ND	ND	ND
132_4	1.00E+04	0.0105	ND	ND	ND	ND	ND	ND
132_2	1000	-0.1866	ND	ND	ND	ND	ND	ND

Table S6: Analysis on NTA sensors, affinity determination of aptamers against PCSK9 (SinoBiological)

Sample ID	Conc. (nM)	Response	KD (M)	KD Error	kon(1/Ms)	kon Error	kdis(1/s)	kdis Error
RMJ24001 Anti-Mouse PCSK9 Antibody (mAb1),AntibodySystem	200	0.6966	1.20E-08	4.21E-10	9.50E+04	2.08E+03	1.14E-03	3.14E-05
RMJ24001 Anti-Mouse PCSK9 Antibody (mAb1),AntibodySystem	20	0.5891	1.30E-09	3.67E-11	5.52E+05	6.22E+03	7.20E-04	1.85E-05
Evolocumab (Repatha)	200	0.4443	4.55E-09	2.81E-10	6.74E+04	8.43E+02	3.07E-04	1.85E-05
Evolocumab (Repatha)	20	0.3168	5.74E-09	1.32E-10	2.01E+05	2.56E+03	1.15E-03	2.22E-05
132_4	1.00E+04	0.128	2.35E-03	4.59E-02	1.35E+01	2.62E+02	3.16E-02	1.82E-03
132_8	1.00E+04	0.1106	3.57E-04	6.54E-04	1.40E+02	2.57E+02	5.00E-02	1.76E-03
132_6	1.00E+04	0.0994	3.80E-04	3.66E-04	4.72E+01	4.54E+01	1.79E-02	3.06E-04
132_3	1.00E+04	0.0927	2.97E-03	6.24E-02	8.42E+00	1.77E+02	2.50E-02	1.23E-03
132_2	1.00E+04	0.0839	1.26E-03	2.20E-02	6.09E+01	1.06E+03	7.66E-02	7.43E-03
132_10	1.00E+04	0.0809	2.96E-04	2.00E-04	1.09E+02	7.38E+01	3.23E-02	5.01E-04
132_9	1.00E+04	0.0725	2.08E-03	6.32E-02	2.61E+01	7.94E+02	5.43E-02	5.54E-03
132_7	1.00E+04	0.0436	ND	ND	ND	ND	ND	ND
132_5	1.00E+04	0.031	ND	ND	ND	ND	ND	ND
132_1	1.00E+04	0.0105	ND	ND	ND	ND	ND	ND

Table S7: Analysis on NTA sensors, affinity determination of aptamers against MMP9

Sample ID	Conc. (µM)	Response	KD (M)	KD Error	kon(1/Ms)	kon Error	kdis(1/s)	kdis Error
Ab anti-MMP9 cat. RHD06804 clone R3D69	0.05	0.9029	8.00E-09	1.62E-10	1.55E+05	1.92E+03	1.24E-03	1.98E-05
Ab anti-MMP9 cat. RHD06804 clone R3D69	0.05	0.4582	2.15E-08	4.34E-10	9.24E+04	1.54E+03	1.99E-03	2.26E-05
Ab anti-MMP9 cat. RHD06804 clone R3D69	0.05	0.3537	1.97E-08	4.00E-10	8.92E+04	1.45E+03	1.76E-03	2.15E-05
Ab anti-MMP9 cat. RHD06804 clone R3D69	0.05	0.2844	3.18E-08	7.26E-10	7.41E+04	1.51E+03	2.36E-03	2.46E-05
140_22	10	0.0937	3.61E-06	1.48E-07	3.63E+02	1.12E+01	1.31E-03	3.55E-05
140_21	10	0.0714	2.35E-08	4.79E-09	5.32E+04	1.05E+04	1.25E-03	6.52E-05
Ab anti-MMP9 cat. RHD06804 clone R3D69	0.005	0.0705	1.24E-09	3.23E-11	1.29E+06	2.43E+04	1.60E-03	2.91E-05
Ab anti-MMP9 cat. RHD06804 clone R3D69	0.005	0.0691	2.04E-10	1.67E-11	1.40E+06	2.12E+04	2.85E-04	2.30E-05
140_23	10	0.0635	2.71E-08	7.61E-09	8.70E+04	2.42E+04	2.36E-03	8.76E-05
140_20	10	0.0455	ND	ND	ND	ND	ND	ND
140_20	1	0.0304	ND	ND	ND	ND	ND	ND
140_23	1	0.0298	ND	ND	ND	ND	ND	ND
140_21	1	0.0288	ND	ND	ND	ND	ND	ND
140_22	1	0.0282	ND	ND	ND	ND	ND	ND
140_24	10	0.0242	ND	ND	ND	ND	ND	ND
140_24	1	0.0204	ND	ND	ND	ND	ND	ND
140_29	10	0.0074	ND	ND	ND	ND	ND	ND
140_25	10	-0.0035	ND	ND	ND	ND	ND	ND
140_26	10	-0.0097	ND	ND	ND	ND	ND	ND
140_28	10	-0.0142	ND	ND	ND	ND	ND	ND
140_27	10	-0.0175	ND	ND	ND	ND	ND	ND
140_25	1	-0.0243	ND	ND	ND	ND	ND	ND
140_27	1	-0.0277	ND	ND	ND	ND	ND	ND
140_29	1	-0.0297	ND	ND	ND	ND	ND	ND
140_26	1	-0.0314	ND	ND	ND	ND	ND	ND
140_28	1	-0.0326	ND	ND	ND	ND	ND	ND

Table S8: Analysis on NTA sensors, affinity determination of aptamers against MMP10

Sample ID	Conc. (µM)	Response	KD (M)	KD Error	kon(1/Ms)	kon Error	kdis(1/s)	kdis Error
140_36	10	0.0289	ND	ND	ND	ND	ND	ND
140_38	10	0.0269	ND	ND	ND	ND	ND	ND
140_34	1	0.0197	ND	ND	ND	ND	ND	ND
140_37	10	0.0193	ND	ND	ND	ND	ND	ND
Ab anti-MMP10 cat. PHC39701	0.01	0.0176	ND	ND	ND	ND	ND	ND
140_32	1	0.0176	ND	ND	ND	ND	ND	ND
Ab anti-MMP10 cat. PHC39701	0.1	0.0165	ND	ND	ND	ND	ND	ND
140_39	10	0.0164	ND	ND	ND	ND	ND	ND
140_31	1	0.0147	ND	ND	ND	ND	ND	ND
140_33	1	0.0135	ND	ND	ND	ND	ND	ND
140_34	10	0.0096	ND	ND	ND	ND	ND	ND
140_31	10	0.0095	ND	ND	ND	ND	ND	ND
Ab anti-MMP10 cat. PHC39701	0.01	0.0078	ND	ND	ND	ND	ND	ND
140_32	10	0.0029	ND	ND	ND	ND	ND	ND
Ab anti-MMP10 cat. PHC39701	0.1	-0.0091	ND	ND	ND	ND	ND	ND
Ab anti-MMP10 cat. PHC39701	0.1	-0.0154	ND	ND	ND	ND	ND	ND
Ab anti-MMP10 cat. PHC39701	0.1	-0.0209	ND	ND	ND	ND	ND	ND
140_33	10	-0.0232	ND	ND	ND	ND	ND	ND

Table S9: Analysis on NTA sensors, affinity determination of aptamers against sRAGE

Sample ID	Conc. (nM)	Response	KD (M)	KD Error	kon(1/Ms)	kon Error	kdis(1/s)	kdis Error
InVivoMab VHH01903	100	2.4858	1.74E-10	3.52E-11	3.52E+05	4.36E+03	6.12E-05	1.24E-05
InVivoMab VHH01903	100	2.4818	1.92E-10	3.39E-11	3.78E+05	4.97E+03	7.27E-05	1.28E-05
InVivoMab VHH01903	10	1.9105	6.12E-10	1.94E-11	5.62E+05	4.18E+03	3.44E-04	1.06E-05
InVivoMab VHH01903	10	1.7337	5.68E-10	4.79E-11	2.31E+05	3.17E+03	1.31E-04	1.09E-05
InVivoMab VHH01903	10	1.577	3.31E-10	1.87E-11	5.66E+05	4.17E+03	1.88E-04	1.05E-05
InVivoMab VHH01903	10	1.4185	<1.0E-12	7.46E-11	1.45E+05	2.97E+03	<1.0E-07	
140_54	1.00E+04	0.2194	3.86E-07	9.24E-08	4.24E+02	1.33E+01	1.64E-04	3.88E-05
140_54	1000	0.0295	ND	ND	ND	ND	ND	ND
140_53	1.00E+04	-0.0036	ND	ND	ND	ND	ND	ND
140_51	1.00E+04	-0.0047	ND	ND	ND	ND	ND	ND
InVivoMab VHH01903	1	-0.006	ND	ND	ND	ND	ND	ND
140_59	1.00E+05	-0.0325	ND	ND	ND	ND	ND	ND
140_59	1000	-0.0423	ND	ND	ND	ND	ND	ND
InVivoMab VHH01903	1	-0.0432	ND	ND	ND	ND	ND	ND
140_50	1.00E+04	-0.0552	ND	ND	ND	ND	ND	ND
140_55	1000	-0.0656	ND	ND	ND	ND	ND	ND
140_56	1000	-0.0807	ND	ND	ND	ND	ND	ND
140_55	1.00E+04	-0.0844	ND	ND	ND	ND	ND	ND
140_50	1000	-0.089	ND	ND	ND	ND	ND	ND
140_52	1.00E+04	-0.0903	ND	ND	ND	ND	ND	ND
140_53	1000	-0.0931	ND	ND	ND	ND	ND	ND
140_52	1000	-0.0969	ND	ND	ND	ND	ND	ND
140_58	1000	-0.1026	ND	ND	ND	ND	ND	ND
140_56	1.00E+04	-0.1046	ND	ND	ND	ND	ND	ND
140_57	1000	-0.1156	ND	ND	ND	ND	ND	ND
140_58	1.00E+04	-0.1454	ND	ND	ND	ND	ND	ND
140_57	1.00E+04	-0.1484	ND	ND	ND	ND	ND	ND
140_51	1000	-0.1748	ND	ND	ND	ND	ND	ND

Table S10: Analysis on NTA sensors, affinity determination of aptamers against ESM1

Sample ID	Conc. (µM)	Response	KD (M)	KD Error	kon(1/Ms)	kon Error	kdis(1/s)
140_45	10	0.1236	1.03E-05	1.19E-06	3.91E+03	4.34E+02	4.01E-02
140_47	10	0.1201	1.43E-05	1.17E-06	1.50E+03	1.18E+02	2.14E-02
140_42	3.33	0.0924	5.00E-06	2.94E-07	3.49E+03	1.98E+02	1.75E-02
140_41	3.33	0.0846	8.44E-06	7.67E-07	2.69E+03	2.40E+02	2.27E-02
140_40	3.33	0.0809	2.56E-06	1.84E-07	8.53E+03	5.79E+02	2.19E-02
140_43	10	0.0797	5.01E-05	1.02E-05	5.85E+02	1.18E+02	2.93E-02
140_49	10	0.0746	5.72E-04	1.48E-03	8.61E+01	2.23E+02	4.93E-02
140_41	10	0.0718	1.83E-04	1.23E-04	2.02E+02	1.36E+02	3.69E-02
140_46	10	0.0547	1.99E-03	2.15E-02	2.95E+01	3.19E+02	5.85E-02
140_44	3.33	0.0206	ND	ND	ND	ND	ND
140_44	10	0.0162	ND	ND	ND	ND	ND
140_40	10	0.0012	ND	ND	ND	ND	ND
140_42	10	-0.0013	ND	ND	ND	ND	ND
140_48	10	-0.0475	ND	ND	ND	ND	ND
140_43	3.33	-0.0653	ND	ND	ND	ND	ND

Quantitative characteristics of aptamer–target protein interactions from BLI analysis on SAX2 sensors (Tables S11–S12)

This section presents the data obtained from BLI analysis on SAX2 sensors, sorted by the response in nm reached on the sensorgram during the analyte association step (after subtraction of the reference-sensors signal). Binding constants are given in exponential format, 1E-12 M corresponds to 1 pM, etc.

Table S11: Analysis on SAX2 sensors, affinity determination of aptamers against MMP2

Loading Sample ID	Conc. (nM)	Response
161_13	253.8	0.0716
161_14	253.8	0.054
161_11	253.8	0.0324
161_12	253.8	0.0225
161_19	253.8	0.0082
161_16	84.6	0.0073
161_14	84.6	0.0006
161_14	28.2	-0.0158
161_13	28.2	-0.0163
161_16	28.2	-0.0212
161_11	28.2	-0.0274
161_13	84.6	-0.0307
161_15	253.8	-0.0394
161_18	253.8	-0.0418
161_15	28.2	-0.0438
161_17	253.8	-0.0457
161_15	84.6	-0.0464
161_12	28.2	-0.0597
161_11	84.6	-0.0601
161_12	84.6	-0.1147
161_19	84.6	-0.1483
161_17	84.6	-0.1777
161_18	84.6	-0.2278
161_19	28.2	-0.2373
161_17	28.2	-0.321
161_18	28.2	-0.3266

Table S12: Analysis on SAX2 sensors, affinity determination of aptamers against PCSK9 (SBT)

Loading Sample ID	Conc. (nM)	Response	KD (M)	KD Error	kon(1/Ms)	kon Error	kdis(1/s)	kdis Error
161_2	1000	0.5601	2.02E-07	6.24E-09	6.02E+03	8.35E+01	1.22E-03	3.36E-05
161_2	333.3	0.2165	1.46E-07	5.51E-09	1.83E+04	5.08E+02	2.66E-03	6.84E-05
161_1	1000	0.1947	2.35E-07	8.69E-09	1.35E+04	3.15E+02	3.16E-03	9.08E-05
161_5	1000	0.0996	2.41E-07	6.81E-09	7.68E+03	1.17E+02	1.85E-03	4.41E-05
161_2	111.1	0.0871	3.16E-07	4.31E-08	7.62E+03	1.03E+03	2.41E-03	5.45E-05
161_1	333.3	0.0618	2.73E-07	1.76E-08	1.34E+04	7.65E+02	3.68E-03	1.10E-04
161_7	333.3	0.0582	4.10E-07	2.86E-08	2.35E+04	1.56E+03	9.63E-03	2.08E-04
161_5	333.3	0.0515	6.66E-06	6.09E-06	7.28E+02	6.65E+02	4.85E-03	1.11E-04
161_10	333.3	0.0409	ND	ND	ND	ND	ND	ND
161_1	1000	0.0392	ND	ND	ND	ND	ND	ND
161_3	1000	0.0386	ND	ND	ND	ND	ND	ND
161_9	333.3	0.0381	ND	ND	ND	ND	ND	ND
161_8	333.3	0.0323	ND	ND	ND	ND	ND	ND
161_3	333.3	0.024	ND	ND	ND	ND	ND	ND
161_1	333.3	0.0207	ND	ND	ND	ND	ND	ND
161_4	333.3	0.0105	ND	ND	ND	ND	ND	ND
161_1	111.1	0.0084	ND	ND	ND	ND	ND	ND
161_9	1000	0.0053	ND	ND	ND	ND	ND	ND
161_4	111.1	-0.0016	ND	ND	ND	ND	ND	ND
161_7	111.1	-0.0048	ND	ND	ND	ND	ND	ND
161_5	111.1	-0.0075	ND	ND	ND	ND	ND	ND
161_1	111.1	-0.01	ND	ND	ND	ND	ND	ND
161_4	1000	-0.0152	ND	ND	ND	ND	ND	ND
161_3	111.1	-0.0157	ND	ND	ND	ND	ND	ND
161_10	1000	-0.0162	ND	ND	ND	ND	ND	ND
161_8	1000	-0.026	ND	ND	ND	ND	ND	ND
161_6	111.1	-0.0294	ND	ND	ND	ND	ND	ND
161_7	1000	-0.0337	ND	ND	ND	ND	ND	ND
161_9	111.1	-0.0378	ND	ND	ND	ND	ND	ND
161_8	111.1	-0.0581	ND	ND	ND	ND	ND	ND
161_10	111.1	-0.0683	ND	ND	ND	ND	ND	ND
161_6	333.3	-0.1773	ND	ND	ND	ND	ND	ND

Supplementary Information S4

Raw (pre-reference-subtraction) and reference-subtracted sensorgrams

Raw and reference-subtracted sensorgrams are compared for three representative aptamer–target pairs (IL6: 140_1/140_6; PCSK9: 161_1–161_5; MMP2: 161_11–161_16), illustrating the magnitude of the reference-sensor correction discussed in Section 2.3 of the main text.

IL6: 140_1 / 140_6 (1 and 10 µM) — left: raw (with reference sensor); right: reference-subtracted

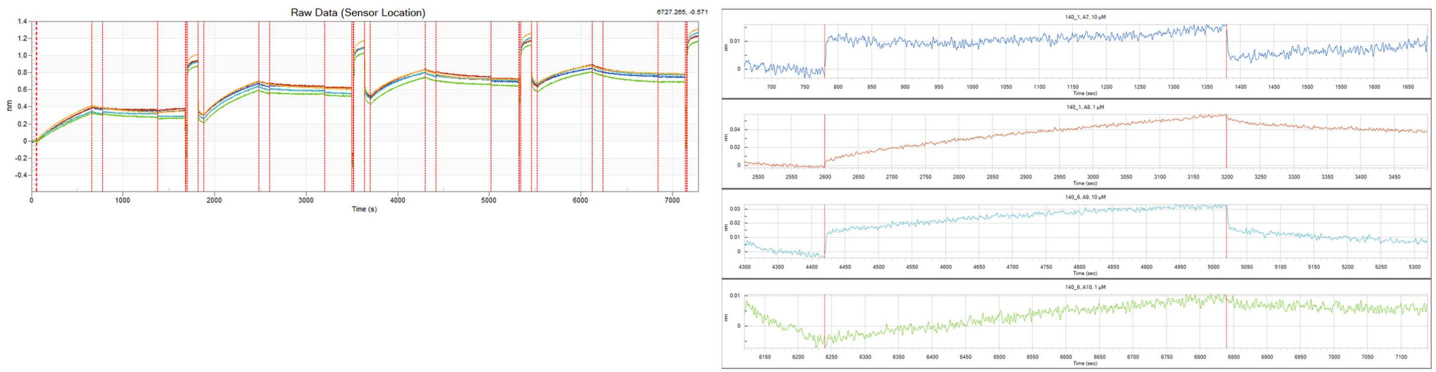

PCSK9: 161_1–161_5 (1000 nM) — left: raw (with reference sensor); right: reference-subtracted

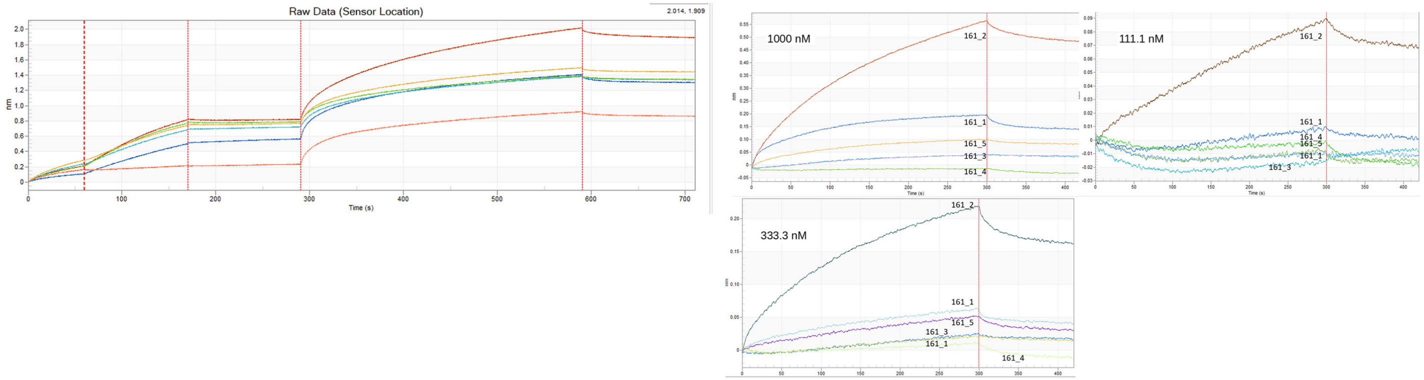

MMP2: 161_11–161_16 (253.8 nM) — left: raw (with reference sensor); right: reference-subtracted

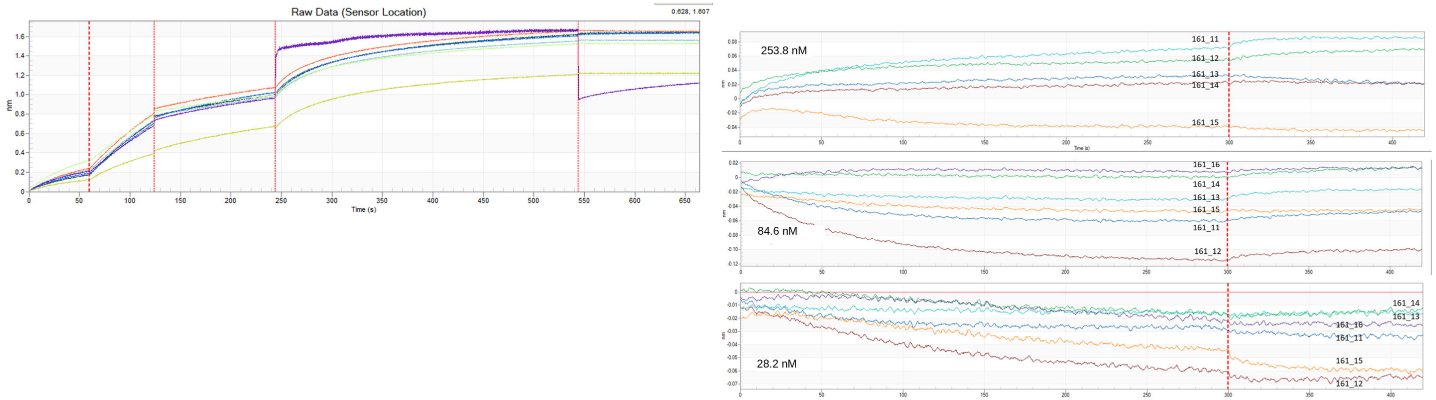

Figure S13: Raw (pre-reference-subtraction) and reference-subtracted sensorgrams. IL6: 140_1/140_6 (1 and 10 µM) – left: raw (with reference sensor); right: reference-subtracted. PCSK9: 161_1–161_5 (1000 nM) – left: raw (with reference sensor); right: reference-subtracted. MMP2: 161_11–161_16 (253.8 nM) – left: raw (with reference sensor); right: reference-subtracted. Note: the sensorgram for sRAGE (140_54) is not included in this composite figure — the raw (pre-subtraction) data for this pair were not retained and are unavailable; the

reference-subtracted sensorgram is shown in Figure S7 (Supplementary Information S2, this document), and its maximum response value (0.219 nm at 10 µM) is reported in Table 3 of the main text.
